# Loss of peptidase D binding restores the tumor suppressor functions of oncogenic p53 mutants

**DOI:** 10.1038/s42003-021-02880-x

**Published:** 2021-12-08

**Authors:** Lu Yang, Yun Li, Arup Bhattacharya, Yuesheng Zhang

**Affiliations:** 1 Department of Pharmacology and Therapeutics, Roswell Park Comprehensive Cancer Center, Buffalo, NY 14263 USA; 2 Department of Urology, Roswell Park Comprehensive Cancer Center, Buffalo, NY 14263 USA

**Keywords:** Oncogenes, Apoptosis

## Abstract

Tumor suppressor p53, a critical regulator of cell fate, is frequently mutated in cancer. Mutation of p53 abolishes its tumor-suppressing functions or endows oncogenic functions. We recently found that p53 binds via its proline-rich domain to peptidase D (PEPD) and is activated when the binding is disrupted. The proline-rich domain in p53 is rarely mutated. Here, we show that oncogenic p53 mutants closely resemble p53 in PEPD binding but are transformed into tumor suppressors, rather than activated as oncoproteins, when their binding to PEPD is disrupted by PEPD knockdown. Once freed from PEPD, p53 mutants undergo multiple posttranslational modifications, especially lysine 373 acetylation, which cause them to refold and regain tumor suppressor activities that are typically displayed by p53. The reactivated p53 mutants strongly inhibit cancer cell growth in vitro and in vivo. Our study identifies a cellular mechanism for reactivation of the tumor suppressor functions of oncogenic p53 mutants.

## Abstract (web summary)

Yang et al. report a mechanism of restoration of tumor suppressor activity of mutant p53 by disrupting its binding to peptidase D, leading to posttranslational modification, refolding and reactivation of the protein and its inhibition of cancer cell growth in vivo and in vitro. This finding presents a new possibility of “reactivating” mutant p53 and reveals a new therapeutic approach for a large number of human tumors.

## Introduction

The p53 tumor suppressor is a major regulator of cell fate and is critical for tumor suppression. It functions by regulating the expression of various genes via sequence-specific DNA binding^1^ and by transcription-independent mechanisms^2^. However, the p53 gene is frequently mutated in human cancers. It occurs, for example, in about 75% and 85% of HER2-positive and triple negative breast cancers^3^. Mutated p53 may lose its tumor suppressor functions or gain oncogenic functions^4^. The majority of p53 mutations are missense mutations, causing one amino acid change in each mutant^5^, but a single amino acid change in p53 may alter its folding state, binding affinity to DNA target sequence, and/or protein–protein interaction^6^.

We recently found that peptidase D (PEPD), also known as prolidase, binds to the proline-rich domain (PRD) in p53 and PEPD knockdown (KD) activates p53 by disrupting its binding to PEPD^7^. Stress signals also activate p53 in part by causing p53 to separate from PEPD^7^. PEPD is a dipeptidase, important for collagen metabolism^8^, but its enzymatic activity is not involved in p53 regulation^7^. p53^F113C^ also binds to PEPD and appears to resemble p53 in response to PEPD KD^7^. While it is difficult to interpret our finding on p53^F113C^, a rare mutant in cancer, because its biology is poorly characterized, it raises the question whether PEPD binds to and regulates other p53 mutants. The PRD in p53 is rarely mutated.

In the present study, we evaluated several hotspot p53 mutants in cancer whose oncogenic activities have been well documented^9^, including p53^R175H^, p53^R248Q^, p53^R273H^, and p53^R280K^. They are contact mutants (R248Q, R273H, R280K) or conformation mutant (R175H)^5,10^. We included p53 for comparison. We hypothesized that PEPD binds to all the p53 mutants and that PEPD KD stimulates their oncogenic activities by disrupting their binding to PEPD. Indeed, our study shows that each p53 mutant binds directly to PEPD. However, to our surprise, all p53 mutants regain tumor-suppressing activities when their binding to PEPD is disrupted by PEPD KD. Our study shows that once freed from PEPD, the p53 mutants undergo posttranslational modifications, especially K373 acetylation, which drive their refolding and reactivation. These findings advance our understanding of the function and regulation of p53 mutants and may pave the way for developing novel cancer treatment strategies.

## Results

### p53 mutants resemble p53 in PEPD binding

**Fig. 1:**
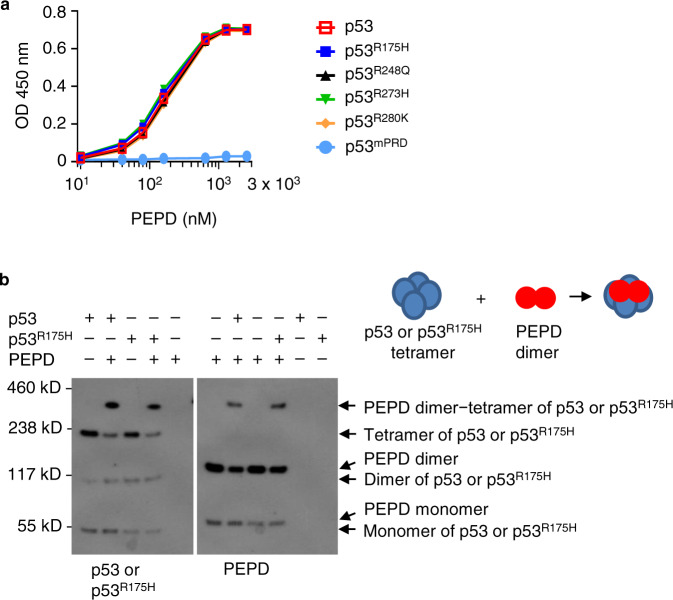
PEPD binds directly to p53 mutants. **a** Recombinant human PEPD (10–2560 nM) was incubated with recombinant human p53 or p53 mutants (250 nM) overnight at 4 °C. Their binding was measured by ELISA. Each value is mean ± SD (*n* = 3). **b** Recombinant human PEPD (801 nM) and p53 (114 nM) or p53^R175H^ (114 nM) were incubated alone or with each other for 15 min at room temperature, then incubated with BS3 (a cross-linker) for 30 min at room temperature, and analyzed by WB for PEPD, p53 and p53^R175H^. p53 was included in each experiment for comparison.

We measured PEPD binding to p53 mutants by enzyme-linked immunosorbent assay (ELISA). p53 was included for comparison. p53^mPRD^, in which 11 of the 12 prolines in the PRD are replaced by alanines, does not bind to PEPD^7^ and was used as a negative control to confirm the specificity of the ELISA. The recombinant proteins were generated in bacteria and purified by affinity chromatography, showing high purity (Supplementary Fig. 1a). PEPD binds to p53, p53^R175H^, p53^R248Q^, p53^R273H^ and p53^R280K^ with almost identical affinity (Kd = 145–185 nM) (Fig. 1a). PEPD exists as a homodimer^11^. By incubating p53 or p53^R175H^ with PEPD, stabilizing their binding by a cross-linker, and analyzing their binding by western blotting (WB), we showed that one PEPD dimer binds to one tetramer of p53 or p53^R175H^ (Fig. 1b). Some PEPD monomers were detected (Fig. 1b), suggesting incomplete cross-linking. Collectively, our results show that p53 mutants bind to PEPD directly and closely resemble p53 in binding affinity and complex formation.

### p53 mutants bind to PEPD in significant quantities in cells

**Fig. 2:**
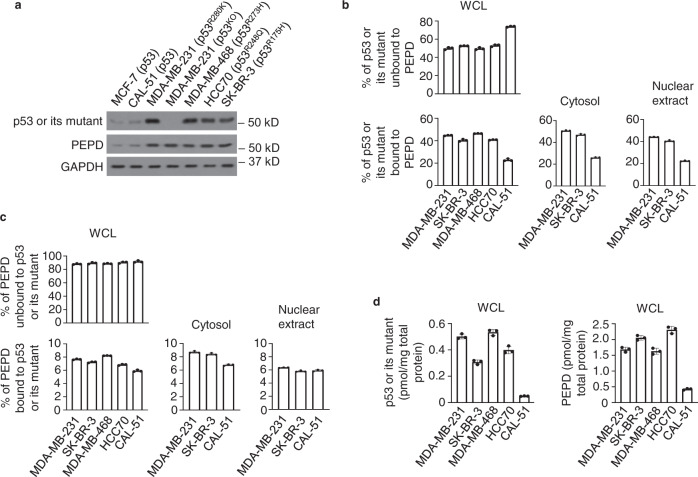
PEPD binds to p53 mutants in cells. **a** WB analysis of p53, p53 mutants and PEPD in WCL of untreated cells. Glyceraldehyde 3-phosphate dehydrogenase (GAPDH) is a loading control. **b**, **c** PEPD, p53 or p53 mutant in a sample (WCL, cytosol or nuclear extract prepared from untreated cells) was completely pulled down by IP. An isotype-matched IgG was used as a control. Percentages of p53 or p53 mutant unbound to PEPD or vice versa were determined by measuring them in the supernatants using ELISA. The precipitates and an input were analyzed by WB for p53, p53 mutant and PEPD (Supplementary Figs. 2 and 3), and the percentages of p53 or p53 mutant bound to PEPD or vice versa were determined by comparing their band intensities with that of the inputs using ImageJ. **d** Levels of p53, p53 mutant and PEPD in WCL of untreated cells, measured by ELISA. The bar-dot plots in **b**–**d** show individual values and mean ± SD (*n* = 3). p53 was included in each experiment for comparison.

We next compared p53 mutants with p53 for PEPD binding in cells, using SK-BR-3 (p53^R175H^), HCC70 (p53^R248Q^), MDA-MB-468 (p53^R273H^), MDA-MB-231 (p53^R280K^), and CAL-51 (p53). The cell lines were first ascertained for suitability, using MDA-MB-231 (p53^KO^) cells and MCF-7 (p53) cells as controls. They are all human breast cancer cells, either HER2-positive (SK-BR-3), estrogen receptor-positive (MCF-7) or triple negative (other cell lines)^12^. MDA-MB-231 (p53^KO^) cells were generated from MDA-MB-231 by CRISPR-Cas9^13^, constituting an isogenic pair. All p53 mutants are homozygous in the cells (Supplementary Fig. 1b). All p53 mutants were overexpressed, as expected, and were accompanied by PEPD overexpression, but PEPD overexpression was not caused by p53 mutants, because it also occurred in MDA-MB-231 (p53^KO^) cells (Fig. 2a). PEPD overexpression was not due to increased gene expression (Supplementary Fig. 1c).

We measured PEPD binding to p53 or its mutants in whole cell lysates (WCL). Each sample was subjected to immunoprecipitation (IP) of PEPD, p53 or p53 mutants. The supernatant was analyzed by WB to confirm complete pull-down of the target protein, and the precipitate was analyzed by WB for its binding partner (Supplementary Fig. 2a, b). The percentages of p53 or its mutants binding to PEPD and vice versa were calculated by comparing their band intensities with that of the inputs. We also measured the proteins in the supernatants by ELISA to determine the percentages of p53 or its mutants unbound to PEPD and vice versa. The combined amount of each protein detected in the precipitates and the corresponding supernatants accounted for 95–97% of PEPD and 93–96% of p53 and its mutants in cells (Fig. 2b, c), validating our experimental approach. Because PEPD is present in both cytosol and nucleus^7^, we also measured PEPD binding to p53 and its mutants in the cytosol and nuclear fraction of several cell lines by IP-WB (Supplementary Fig. 3a, b). Cross contamination between the cytosol and nuclear extract was ruled out (Supplementary Fig. 3c). Approximately 23% of p53 and 40–47% of p53 mutants bind to PEPD, whether it is measured in WCL, cytosol or nuclear extract (Fig. 2b). Only 6–9% of PEPD bind to p53 and its mutants (Fig. 2c), although cellular levels of PEPD are 3.1–8.7-fold higher than that of p53 and its mutants (Fig. 2d). Collectively, our results show that a small percentage of PEPD binds to nearly half of each p53 mutant in cells.

### p53 mutants separated from PEPD are reactivated

**Fig. 3:**
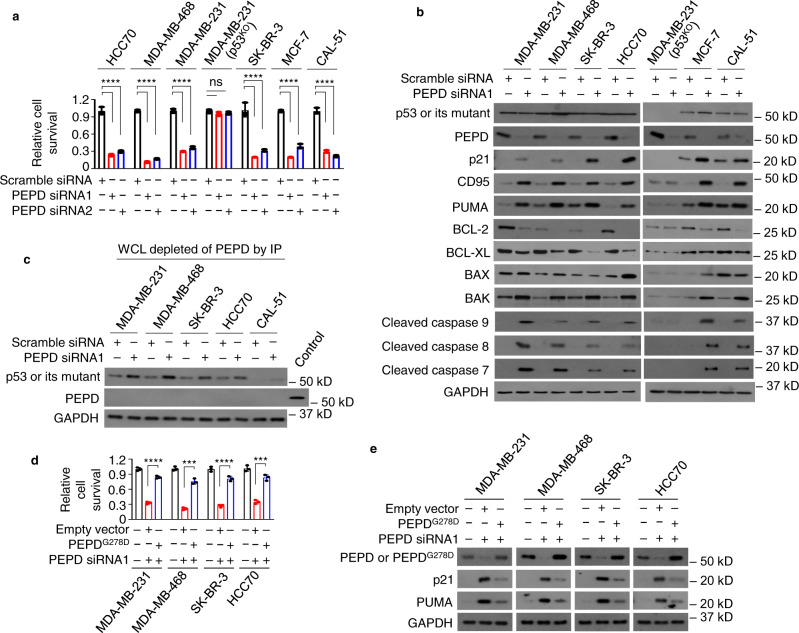
PEPD KD reactivates p53 mutants in cells by disrupting their binding to PEPD. **a** Cells were treated by siRNA (10 nM) for 72 h. Cell viability were measured by trypan blue assay. **b** Cells were treated by siRNA (10 nM) for 48 h. p53 and other proteins in WCL were analyzed by WB. **c** Cells were treated by siRNA (10 nM) for 48 h. PEPD, PEPD-p53 complexes, and PEPD-p53 mutant complexes in WCL were pulled down by PEPD IP, and the supernatant was analyzed for p53, p53 mutants and PEPD by WB. WCL of untreated MDA-MB-231 cells was used as a PEPD control in WB. **d**, **e** Cells were transfected with or without PEPD^G278D^, and 24 h later treated by vehicle or PEPD siRNA (10 nM) for 72 h. Cell viability was measured by trypan blue assay in **d**. PEPD and other proteins in WCL were analyzed by WB in **e**. Cells carrying p53 (MCF-7 cells and CAL-51 cells) or MDA-MB-231 (p53^KO^) cells were included in **a**–**c** for comparison. GAPDH is a loading control in **b**, **c**, and **e**. The bar-dot plots in **a** and **d** show individual values and mean ± SD (*n* = 3). ***P < 0.001, ****P < 0.0001, ns—not significant, by analysis of variance followed by Tukey test in **a** or by paired two-tailed *t*-test in **d**.

We next investigated the consequence of disrupting PEPD binding to p53 mutants by PEPD KD, including p53 for comparison. MDA-MB-231 (p53^KO^) cells were also included to rule out effects independent of p53 mutants. We used two siRNAs targeting the 3′-untranslated region (siRNA1) or exon 12 (siRNA2) of *PEPD*, and scramble siRNA as a control. Each PEPD siRNA inhibited the survival of cells carrying a p53 mutant or p53 by 64–89% at 72 h after transfection, including SK-BR-3, MDA-MB-231, MDA-MB-468, HCC70, CAL-51, and MCF-7, but had little effect on MDA-MB-231 (p53^KO^) cells (Fig. 3a). PEPD KD was pronounced in all the cell lines, while neither p53 mutants nor p53 changed their expression levels (Fig. 3b; Supplementary Fig. 4a). We showed previously that PEPD KD activates p53 without changing its expression^7^. We measured the expression of several p53 target proteins. PEPD siRNA induced p21, CD95, PUMA, and BAK, and reduced BCL-2 in all the cell lines except MDA-MB-231 (p53^KO^) cells (Fig. 3b; Supplementary Fig. 4a). PEPD siRNA also induced BAX and/or reduced BCL-XL in these cells except MDA-MB-231 (p53^KO^) cells (Fig. 3b). Because the two PEPD siRNAs showed no difference in activity, we used PEPD siRNA1 in subsequent experiments. C911 siRNA was reported to be a better control than scramble siRNA for off target effects^14^. However, PEPD siRNA1 caused the same changes in cells with C911 siRNA as a control (Supplementary Fig. 4b, c). We also showed that PEPD siRNA1 strongly inhibited cell survival, whether it was measured by trypan blue exclusion assay (Fig. 3a) or the CellTiter-Glo assay (Supplementary Fig. 4c). p21 is known to inhibit cell cycle at G1/S phase. Indeed, PEPD siRNA1 had no effect on cell cycle in MDA-MB-231 (p53^KO^) cells but caused S phase arrest in other cells (Supplementary Fig. 4d). PEPD siRNA also activated multiple caspases (caspase 9, 8, 7) in all the cell lines except MDA-MB-231 (p53^KO^) cells (Fig. 3b; Supplementary Fig. 4a, b). These results suggest that PEPD KD reactivates p53 mutants. However, PEPD KD activated caspase 3 in cells carrying p53 but not in cells carrying any p53 mutant (Supplementary Fig. 4e).

Because only a small amount of cellular PEPD bind to p53 mutants, it was important to determine whether PEPD KD disrupts PEPD binding to p53 mutants. We included p53 for comparison. Cells were treated with scramble or PEPD siRNA for 48 h, and WCL from such cells were subjected to PEPD IP to remove all PEPD, including free PEPD and those bound to p53 mutants and p53. Notably, PEPD siRNA does not change the overall cellular levels of p53 mutants and p53, and over 50% of a p53 mutant and p53 in cells do not bind to PEPD, as shown before. No PEPD remained in the supernatant after PEPD IP, but PEPD siRNA increased supernatant levels of p53 mutants and p53, indicating that PEPD KD frees p53 mutants from PEPD (Fig. 3c).

We asked whether PEPD^G278D^, an enzymatically inactive PEPD mutant that binds to PRD in p53^7^, could neutralize the effects of PEPD siRNA on p53 mutants. Cells were transfected with PEPD^G278D^ and 24 h later treated with PEPD siRNA1 for 72 h. PEPD siRNA1 targets the 3′-UTR of PEPD mRNA as mentioned before, but PEPD^G278D^ mRNA lacks 3′-UTR. PEPD siRNA1 killed 65-79% of cells without PEPD^G278D^ but only 16–25% of cells with PEPD^G278D^ (Fig. 3d). The PEPD^G278D^ rescue is highly effective, since gene transfection efficiency is unlikely 100%. In cells transfected with PEPD^G278D^, PEPD^G278D^ level was high, and there was little induction of p21 and PUMA, despite PEPD siRNA1 treatment (Fig. 3e). These results further show that reactivation of p53 mutants by PEPD siRNA results from their separation from PEPD and suggest that the enzymatic activity of PEPD plays no role in such reactivation.

### The oncogenic functions of p53 mutants do not require PEPD

**Fig. 4:**
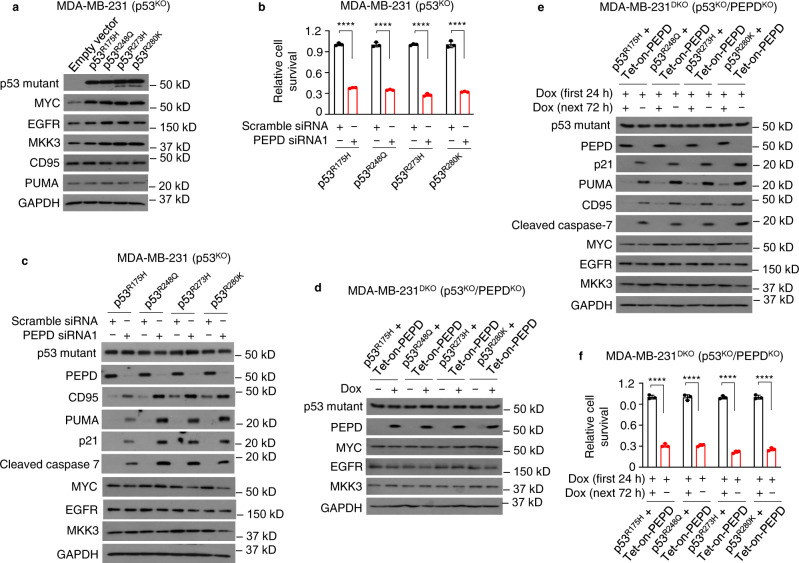
PEPD is not required for the oncogenic functions of p53 mutants, and reactivated p53 mutants dominate their unreactivated counterparts. **a** MDA-MB-231 (p53^KO^) cells were transfected with or without a p53 mutant for 48 h. p53 mutant and other proteins in WCL were analyzed by WB. **b**, **c** MDA-MB-231 (p53^KO^) cells were transfected with a p53 mutant and 24 h later treated with siRNA (10 nM) for 96 h. The relatively long siRNA treatment time was for harvesting enough cells for analysis. Cell viability was measured by trypan blue assay in **b**. p53 mutant and other proteins in WCL were analyzed by WB in **c**. **d** MDA-MB-231^DKO^ cells (p53^KO^ and PEPD^KO^) were cotransfected with a p53 mutant and Tet-on-PEPD and treated with or without Dox (50 ng/ml) for 48 h. p53 mutant and other proteins in WCL were analyzed by WB. **e**, **f** MDA-MB-231^DKO^ cells were transfected with a p53 mutant and Tet-on-PEPD, treated with Dox (50 ng/ml) for 24 h, washed, and cultured with or without Dox (50 ng/ml) for 72 h. p53 mutant and other proteins in WCL were analyzed by WB in **e**. Cell viability was measured by trypan blue assay in **f**. GAPDH is a loading control in **a** and **c–e**. The bar-dot plots in **b** and **f** show individual values and mean ± SD (*n* = 3). ****P < 0.0001, by paired two-tailed *t*-test.

c-MYC (MYC), epidermal growth factor receptor (EGFR), and mitogen-activated protein kinase kinase 3 (MKK3) are among the oncoproteins that are typically induced by various oncogenic p53 mutants^5^. Transfection of each of the four p53 mutants into MDA-MB-231 (p53^KO^) cells induced MYC, three of which also induced EGFR and MKK3, but no mutant induced CD95 and PUMA (Fig. 4a). If MDA-MB-231 (p53^KO^) cells transfected with a p53 mutant were treated with PEPD siRNA, cell survival was markedly inhibited (Fig. 4b), which was accompanied by no change in p53 mutant expression, marked PEPD KD, induction of CD95, PUMA and p21, and activation of caspase 7, but no increase or slight decrease in MYC, EGFR, and MKK3 (Fig. 4c). Because 40–47% of each p53 mutant in cells bind to PEPD as described before, we asked whether PEPD is required for the oncogenic functions of p53 mutants. We generated MDA-MB-231^DKO^ cells (p53^KO^ and PEPD^KO^) from MDA-MB-231 (p53^KO^) cells using CRISPR-Cas9 and cotransfected into them a p53 mutant and tetracycline-inducible PEPD (Tet-on-PEPD). PEPD was strongly induced by doxycycline (Dox), but PEPD induction did not change the expression levels of MYC, EGFR, and MKK3 (Fig. 4d). PEPD KD by withdrawing Dox did not alter the expression of p53 mutants but strongly induced p21, PUMA and CD95, activated caspase 7, without inducing MYC, EGFR, and MKK3 (Fig. 4e), and inhibited cell survival by 69–78% (Fig. 4f). MYC was somewhat reduced by PEPD KD. Overall, these results suggest that PEPD is not required for the oncogenic functions of p53 mutants, that reactivation of p53 mutants by PEPD KD is not siRNA-specific, and that reactivated p53 mutants dominate their unreactivated counterparts in determining cell fate.

### Non-transcriptional activities of reactivated p53 mutants

**Fig. 5:**
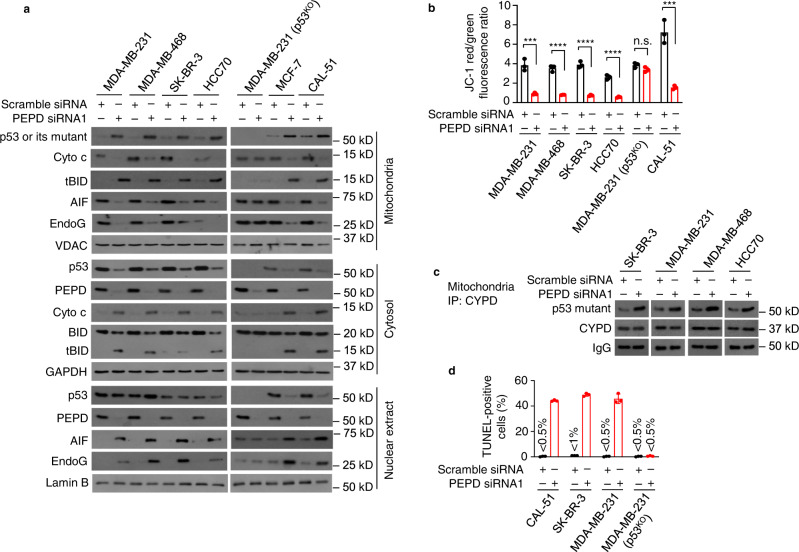
PEPD KD reactivates the transcription-independent tumor suppressor activities of p53 mutants. **a** Cells were treated by siRNA (10 nM) for 48 h. p53, p53 mutants and other proteins in subcellular fractions were analyzed by WB, using voltage-dependent anion channel (VDAC), GAPDH and lamin B as loading controls. **b** Cells were treated by siRNA (10 nM) for 48 h. MMP was measured by JC-1 fluorescence. **c** Cells were treated by siRNA (10 nM) for 48 h, from which mitochondria were isolated and analyzed by IP-WB for CYPD binding to p53 mutants. **d** Cells were treated by siRNA (10 nM) for 72 h. Apoptosis was measured by TUNEL assay, counting 500 cells per sample (see also Supplementary Fig. 5). The bar-dot plots in **b** and **d** show individual values and mean ± SD (*n* = 3). ****P < 0.0001, by paired two-tailed *t*-test in **b**. Cells carrying p53 (MCF-7 cells and CAL-51 cells) or MDA-MB-231 (p53^KO^) cells were included in **a, b**, and **d** for comparison.

We previously showed that PEPD KD causes p53 to enrich in mitochondria^7^. We investigated subcellular distribution of p53 mutants upon PEPD KD, including p53 for comparison. PEPD KD increased mitochondrial levels of each p53 mutant and p53 with reciprocal decrease in the cytosol (Fig. 5a). PEPD KD also decreased nuclear level of p53, but nuclear levels of p53 mutants varied from no change (p53^R280K^ in MDA-MB-231 cells, and p53^R175H^ in SK-BR-3 cells), increase (p53^R273H^ in MDA-MB-468 cells), to decrease (p53^R248Q^ in HCC70 cells), although PEPD KD was pronounced in both cytosol and nucleus of each cell line (Fig. 5a). Enrichment of p53 mutants and p53 in mitochondria was accompanied by increase in mitochondrial tBID, decrease in mitochondrial cytochrome c (Cyto c), apoptosis-inducing factor (AIF) and endonuclease G (EndoG), increase in cytosolic Cyto c and tBID, decrease in cytosolic BID, and increase in nuclear AIF and EndoG (Fig. 5a). Caspase 8 converts BID to proapoptotic tBID which migrates to mitochondria to promote Cyto c release^15^, and caspase 8 is activated upon PEPD KD as described before. Nuclear translocation of AIF and EndoG and cytosolic translocation of Cyto c from mitochondria are also well-established mechanisms of mitochondria-mediated apoptosis. However, although PEPD KD was pronounced in both cytosol and nucleus of MDA-MB-231 (p53^KO^) cells, there was no subcellular redistribution of AIF, EndoG, Cyto c, BID and tBID in these cells (Fig. 5a).

Consistent with the molecular changes described above, PEPD KD caused marked loss of mitochondria membrane potential (MMP) in cells carrying a p53 mutant or p53, and the extent of MMP loss was similar across cell lines with different p53 genotype, but PEPD KD did not significantly alter MMP in MDA-MB-231 (p53^KO^) cells (Fig. 5b). Mitochondrial p53 causes MMP loss and cell death by binding to cyclophilin D (CYPD) to open mitochondrial permeability transition pore^16^ and by binding to BCL-2 and BCL-XL to neutralize their inhibitory effects on BAK and BAX^17^. Focusing on CYPD, we showed that PEPD siRNA markedly increases the binding of all p53 mutants to CYPD in mitochondria (Fig. 5c). Using TUNEL assay, we further showed that PEPD siRNA strongly induced apoptosis in cells carrying p53 or a mutant but was inactive in p53^KO^ cells (Fig. 5d; Supplementary Fig. 5). Our results show that PEPD KD reactivates the transcription-independent, mitochondria-mediated tumor suppressor functions of p53 mutants.

p53 translocation to mitochondria is driven by MDM2-mediated mono-ubiquitination^18,19^. MDM2 at high level of activity causes polyubiquitination and degradation of p53 but at low level of activity causes mono-ubiquitination of p53^19^. Focusing on p53^R175H^ in SK-BR-3 cells, we showed that MDM2 KD by siRNA blocks PEPD KD-induced mitochondrial enrichment of p53^R175H^ (Supplementary Fig. 6a) and that PEPD KD increases MDM2 binding to p53^R175H^ and enriches mono-ubiquitinated p53^R175H^ in mitochondria (Supplementary Fig. 6b, c). MDM2 binds to amino acids #18–26 of p53^20^, not far from the PRD (amino acids #64–94) to which PEPD binds. Thus, PEPD and MDM2 are likely mutually exclusive for binding to p53 mutants. However, PEPD KD did not induce mono-ubiquitination of nuclear p53^R175H^ (Supplementary Fig. 6c). This may explain why nuclear p53^R175H^ level does not decrease upon PEPD KD.

### Reactivation of transcriptional activities of p53 mutants

**Fig. 6:**
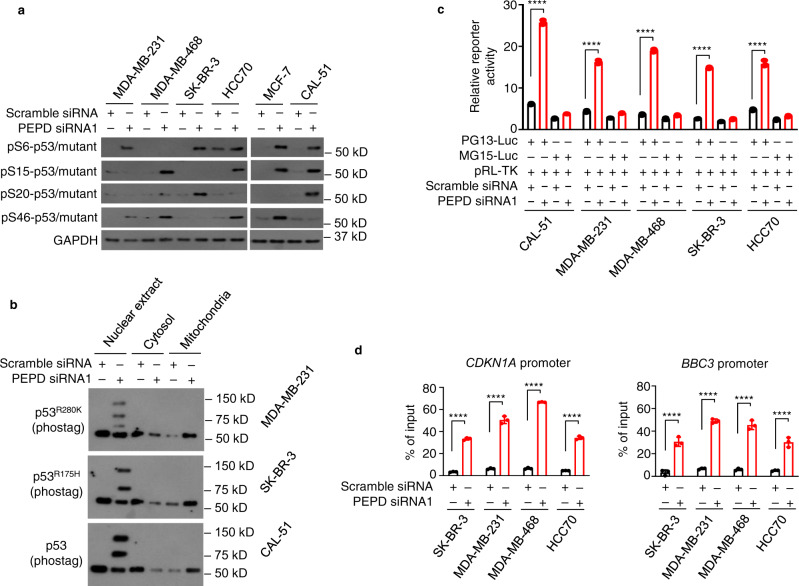
PEPD KD reactivates the transcription-dependent tumor suppressor activities of p53 mutants. **a** Cells were treated by siRNA (10 nM) for 24 h. Phospho-p53 and phospho-p53 mutants in WCL were analyzed by WB. GAPDH is a loading control. **b** Cells were treated by siRNA (10 nM) for 48 h. Phospho-p53 and phospho-p53 mutants in subcellular fractions were analyzed by phostag WB. **c** Cells were transfected with an equal amount of PG13-Luc or MG15-Luc along with pRL-TK and 24 h later treated with siRNA (10 nM) for 48 h. Luciferase activity was measured in WCL. **d** Cells were treated by siRNA (10 nM) for 48 h. Binding of p53 mutants to the p53-binding sites in the promoters of *CDKN1A* and *BBC3* genes were measured by ChIP-qPCR assay. The bar-dot plots in **c** and **d** show individual values and mean ± SD (*n* = 3). ****P < 0.0001, by paired two-tailed *t*-test. Cells carrying p53 (MCF-7 cells and CAL-51 cells) or MDA-MB-231 (p53^KO^) cells were included in **a–c** for comparison.

Our results suggested that PEPD KD also reactivates transcriptional functions of p53 mutants (e.g., Figs. 3b and 4c, e). This was further investigated, using p53 for comparison. Because phosphorylation is crucial for activation of p53 transcriptional activity, we compared p53 mutants and p53 for phosphorylation in the transactivation domains (S6, S15, S20, and S46). PEPD KD induced phosphorylation in all the proteins, but the phosphorylation sites varied among them. The variability may be partly related to cell context, because phosphorylation sites in p53 differed between MCF-7 cells and CAL-51 cells (Fig. 6a; Supplementary Fig. 7a). Because there was no shared phosphorylation site, we used phostag WB, which detects overall phosphorylation, to determine the cellular location of phosphorylated proteins, focusing on p53^R175H^, p53^R280K^, and p53. Phosphorylation of the proteins induced by PEPD KD occurred in the nucleus but not in the cytosol or mitochondria (Fig. 6b). When PEPD-bound p53 mutants and p53 in the nuclear extract were removed by PEPD IP, their phosphorylation was detected in the supernatant, not in the precipitate (Supplementary Fig. 7b), indicating that they were phosphorylated after leaving PEPD.

PG13-Luc is a p53 reporter, which contains multiple copies of p53-binding site, whereas MG15-Luc in which the p53-binding site is mutated does not respond to p53^21^. Cells were transfected with PG13-Luc or MG15-Luc, along with pRL-TK for control of transfection efficiency, and 24 h later treated with siRNA for 48 h. PEPD siRNA induced reporter expression from PG13-Luc by 4.2-fold in cells carrying p53 and 3.3–5.6-fold in cells carrying a p53 mutant (Fig. 6c). In contrast, PEPD siRNA had little effect on MG15-Luc reporter expression in all the cell lines. Using ChIP-qPCR, we further showed that in cells treated with PEPD siRNA for 48 h, binding of each p53 mutant to the p53-binding site in the promoter of *CDKN1A* gene encoding p21 increased 7.6–10.2-fold and in the promoter of *BBC3* encoding PUMA increased 5.9–7.6-fold (Fig. 6d).

Collectively, our results reveal that nuclear p53 mutants, once freed from PEPD due to PEPD KD, are phosphorylated and regulate gene expression by binding to the p53-binding site in gene promoter. The reactivated transcriptional activities of p53 mutants are almost indistinguishable from that of activated p53.

### Reactivation of p53 mutants requires K373 acetylation

**Fig. 7:**
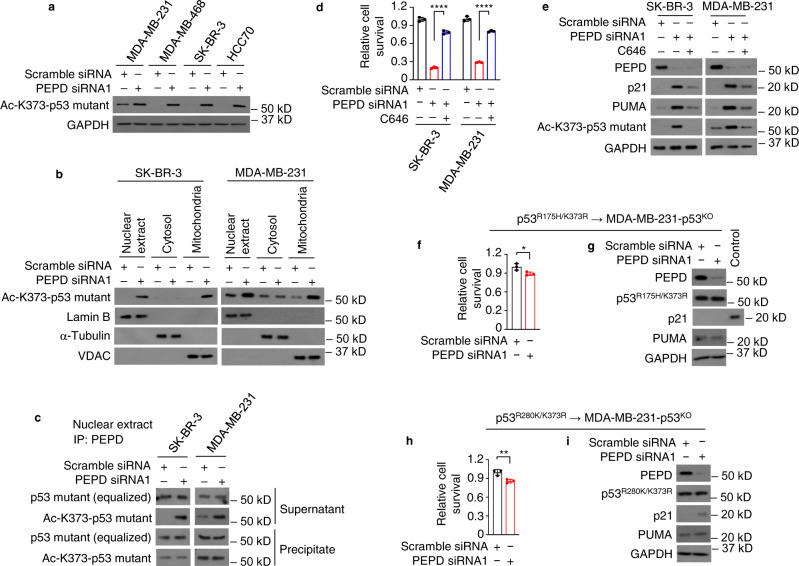
K373 acetylation is essential for reactivation of p53 mutants. **a, b** Cells were treated by siRNA (10 nM) for 48 h. Ac-K373-p53 mutants in WCL in **a** and in subcellular fractions in **b** were analyzed by WB. **c** Cells were treated by siRNA (10 nM) for 48 h. PEPD-p53 mutant complexes were removed from the nuclear extracts by PEPD IP. The precipitate and the supernatant were analyzed by WB for p53 mutants and Ac-K373-p53 mutants. **d**, **e** Cells were treated by siRNA (10 nM) with or without C646 (8 μM) for 72 h. Cell viability was measured by trypan blue assay in **d**. PEPD and other proteins in WCL were analyzed by WB in **e**. **f–i** MDA-MB-231 (p53^KO^) cells were transfected with p53^R175H/K373R^ or p53^R280K/K373R^ and 24 h later treated by siRNA (10 nM) for 96 h. The relatively long siRNA treatment time was for harvesting enough cells for analysis. Cell viability was measured by trypan blue assay in **f** and **h**. PEPD and other proteins in WCL were analyzed by WB in **g** and **i**. A p21-positive sample (WCL of SK-BR-3 cells treated by PEPD siRNA) was used as a control in **g**. GAPDH is a loading control in **a**, **e**, **g**, and **i**. α-Tubulin, lamin B and VDAC are loading controls and for ruling out cross contamination in **b**. The bar-dot plots in **d**, **f**, and **h** show individual values and mean ± SD (*n* = 3). *P < 0.05, **P < 0.01, ****P < 0.0001, by paired two-tailed *t*-test.

Acetylation of p53 is critical for its activation^22, 23^. Acetylation was also reported to be important for reactivation of certain p53 mutants^24, 25^. We measured the effect of PEPD KD on acetylation of p53 mutants at ten lysine residues, including K120, K305, K319, K320, K370, K372, K373, K381, K382, and K386. PEPD KD caused acetylation at various sites, but only K373 acetylation is shared by all the mutants (Fig. 7a; Supplementary Fig. 8a). No mutant showed K370 acetylation and K372 acetylation was negligible. Using p53^R175H^ and p53^R280K^, we showed that Ac-K373-p53 mutants exist in the nucleus and mitochondria but not in the cytosol (Fig. 7b). We asked whether K373 acetylation occurs to the mutants bound to PEPD. We studied nuclear extracts, since PEPD is not in mitochondria. We removed p53 mutants bound to PEPD from nuclear extracts by PEPD IP and measured Ac-K373-p53 mutants in the precipitate and supernatant. Ac-K373-p53 mutants, resulting from PEPD KD, were detected only in the supernatant (Fig. 7c). Thus, K373 acetylation occurred after the mutants left PEPD.

C646 inhibits p300/CBP that acetylates p53^26^. C646 prevented PEPD KD from causing death of cells carrying a p53 mutant (Fig. 7d; Supplementary Fig. 8b). C646 also blocked K373 acetylation of p53 mutants and induction of p53 target proteins, including p21 and PUMA, despite profound PEPD KD (Fig. 7e; Supplementary Fig. 8c). To further clarify the role of K373 acetylation in reactivation of p53 mutants, we generated p53^R175H/K373R^, p53^R248Q/K373R^, p53^R273H/K373R^, and p53^R280K/K373R^, expressed each new mutant in MDA-MB-231 (p53^KO^) cells, and then subjected the cells to PEPD KD. The mutants bind to PEPD normally as described later but were not notably reactivated by PEPD KD, as assessed by cell survival and induction of p21 and PUMA (Fig. 7f–i; Supplementary Fig. 8d, e). In contrast, p53^R175H^, p53^R248Q^, p53^R273H^ and p53^R280K^ transfected to MDA-MB-231 (p53^KO^) cells were strongly reactivated by PEPD KD (Fig. 4b, c). Thus, K373 acetylation is key to the reactivation of p53 mutants by PEPD KD.

### p53 mutants refold after dissociating from PEPD

**Fig. 8:**
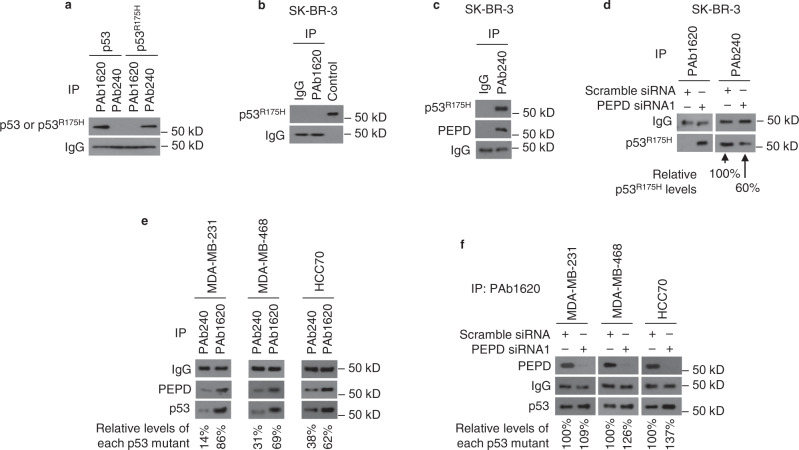
p53 mutants refold once separated from PEPD. **a** Recombinant p53 and p53^R175H^ were subjected to IP by PAb1620 or PAb240, followed by WB analysis of the precipitate for p53 and p53^R175H^. **b**–**f** WCL of untreated cells in **b**, **c**, and **e** or WCL of cells treated with siRNA (10 nM) for 48 h in **d** and **f** were subjected to IP by PAb1620, PAb240, or isotype-matched IgG, followed by WB analysis of the precipitate for p53 mutants and PEPD. A p53-containing sample (WCL of untreated CAL-51 cells) was used as a positive control during WB in **b**. The relative level of each p53 mutant in **d–f** was measured by ImageJ.

We asked whether PEPD scaffolds p53 mutants back to wild-type (WT) conformation. Antibodies PAb1620 and PAb240 detect p53 in “WT” and “denatured” conformation, respectively^6, 27^, which was confirmed using recombinant p53 and p53^R175H^ (Fig. 8a). p53^R175H^ is known to exist entirely in “denatured” conformation^28^. Indeed, p53^R175H^ in SK-BR-3 WCL did not bind to PAb1620 but bound to PAb240 (Fig. 8b, c). PEPD was pulled down with p53^R175H^ by PAb240 (Fig. 8c), indicating that p53^R175H^ bound to PEPD remains in “denatured” conformation. About 40% of p53^R175H^ molecules switched from “denatured” to “WT” conformation after PEPD KD (Fig. 8d). The refolding of about 40% p53^R175H^ closely matches the percentage of p53^R175H^ initially bound to PEPD in SK-BR-3 cells (Fig. 2b), indicating that most if not all p53^R175H^ molecules refold after leaving PEPD. We also examined p53^R248Q^, p53^R273H^ and p53^R280K^ in WCL, using PAb240 and PAb1620, and found that each mutant exists mainly in “WT” conformation, with the rest in “denatured” conformation (Fig. 8e), which is consistent with literature data^28^. PEPD was pulled down with each p53 mutant by IP with PAb1620 or PAb240, but mainly with PAb1620 (Fig. 8e). Thus, PEPD binds to p53 mutants regardless of their conformation and does not induce refolding of the mutants. However, PEPD KD increased the levels of the three mutants in “WT” conformation (Fig. 8f), and the relatively small increases closely match the relatively small pools of the mutant molecules existing initially in “denatured” conformation. Because PEPD KD does not change the total levels of p53 mutants in cells as shown before, the above results indicate that most if not all p53 mutant molecules that were initially in “denatured” conformation changed to “WT” conformation after leaving PEPD. Collectively, our results show that PEPD binding to p53 mutants does not change their conformation but the mutants refold after leaving PEPD.

### K373 acetylation is essential for p53 mutants to refold

**Fig. 9:**
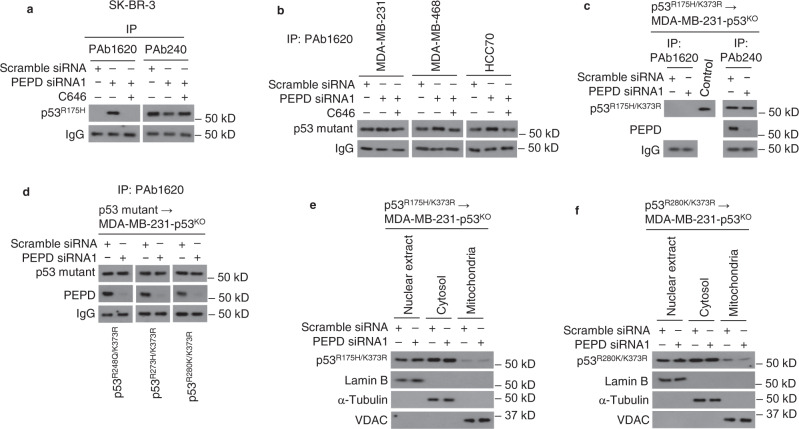
K373 acetylation is required for refolding and mitochondrial enrichment of p53 mutants freed from PEPD. **a**, **b** Cells were treated by siRNA (10 nM) for 48 h with or without C646 (8 μM). WCL was subjected to IP by PAb1620 or PAb240, followed by WB analysis of the precipitate for p53 mutants. **c, d** MDA-MD-231 (p53^KO^) cells were transfected with p53^R175H/K373R,^ p53^R248Q/K373R^, p53^R273H/K373R^, or p53^R280K/K373R^ and 24 h later treated with siRNA (10 nM) for 48 h. WCL was subjected to IP by PAb1620 or PAb240, followed by WB analysis of the precipitate by p53 mutants and PEPD. A p53-containing sample (WCL of untreated CAL-51 cells) was used as positive control during WB in **c**. **e**, **f** MDA-MD-231 (p53^KO^) cells were transfected with p53^R175H/K373R^ or p53^R280K/K373R^ and 24 h later treated with siRNA (10 nM) for 48 h. p53 mutants in subcellular fractions was analyzed by WB. Lamin B, α-tubulin and VDAC were measured as loading controls and for ruling out cross contamination.

C646 blocked PEPD KD-induced conformation change of all four p53 mutants from “denatured” to “WT” (Fig. 9a, b). We next expressed p53^R175H/K373R^, p53^R248Q/K373R^, p53^R273H/K373R^ or p53^R280K/K373R^ in MDA-MB-231 (p53^KO^) cells and subjected the cells to PEPD KD. K373R mutation did not disrupt binding of each p53 mutant to PEPD but almost completely blocked conformation change of the mutants induced by PEPD KD (Fig. 9c, d). Therefore, K373 acetylation, which occurs after p53 mutants leave PEPD as described before, enables refolding of the mutants. Moreover, using p53^R175H/K373R^ and p53^R280K/K373R^, we showed that neither mutant, when expressed in MDA-MB-231 (p53^KO^) cells, enriched in mitochondria upon PEPD KD (Fig. 9e, f). This indicates that p53 mutants must refold before translocating to mitochondria.

### Reactivated p53 mutants strongly inhibit tumor growth

**Fig. 10:**
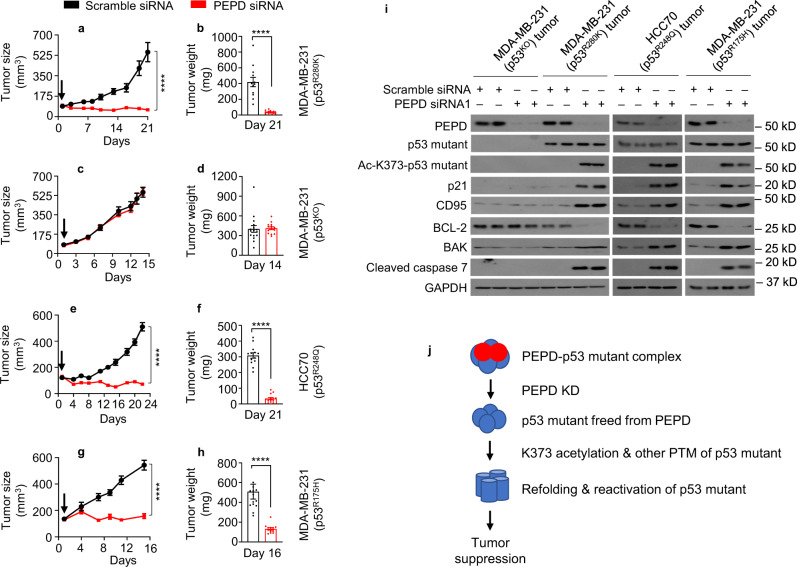
p53 mutants reactivated by PEPD KD in vivo inhibit tumor growth. **a–i** Mice bearing orthotopic breast tumors generated from MDA-MB-231 (p53^R280K^) cells, MDA-MB-231 (p53^KO^) cells, HCC70 (p53^R248Q^) cells, or MDA-MB-231 (p53^R175H^) cells were treated by intratumor injection of siRNA (10 pmol) every 3 days. Arrows in **a**, **c**, **e**, and **g** indicate treatment start. The experiments were stopped 24 or 48 h after the last siRNA dose. Treatment of HCC70 (p53^R248Q^) tumors was suspended for 7 days before the final dose, because the tumors in the PEPD siRNA group became too small to treat. Each value in **a**–**h** is mean ± SEM (*n* = 13–16). The bar plots in **b**, **d**, **f**, and **h** show individual values and mean ± SEM (*n* = 13–16). ****P < 0.0001, by paired two-tailed *t*-test. PEPD and other proteins in tumor homogenates (two tumors per group) were analyzed by WB (**i**). GAPDH is a loading control. **j** Graphic representation of reactivation of p53 mutants by PEPD KD. A PEPD homodimer binds to a homo-tetramer of p53 mutants. p53 mutants freed from PEPD due to PEPD KD undergo K373 acetylation and other PTM (mono-ubiquitination and phosphorylation), which result in refolding of the mutants and reactivation of their transcription-dependent and transcription-independent tumor-suppressing activities.

Intratumor injection of siRNA was previously shown to silence PEPD and other genes^7, 29, 30^. Using this approach, we compared the effects of PEPD KD on isogenic orthotopic tumors with or without a p53 mutant. We inoculated MDA-MB-231 (p53^R280K^) cells or MDA-MB-231 (p53^KO^) cells to the mammary fat pads of female SCID mice. To evaluate a p53 mutant in a tumor line different from MDA-MB-231, we inoculated HCC70 cells (p53^R248Q^) to the mammary fat pads of female SCID mice. Once average tumor size reached about 100–130 mm^3^, intratumor injection of scramble or PEPD siRNA (10 pmol) was given once every 3 days. The experiment was stopped when average tumor size in the control reached about 550 mm^3^, to minimize impediment to siRNA tissue distribution. MDA-MB-231 (p53^KO^) tumors and MDA-MB-231 (p53^R280K^) tumors were treated without interruption, but treatment of HCC70 (p53^R248Q^) tumors was suspended for 1 week before the last dose, because the tumors in the PEPD siRNA group became too small to treat. The tumors were collected from the mice 1 and 2 days after the final treatment, respectively. All the tumors grew rapidly on scramble siRNA. PEPD siRNA inhibited MDA-MB-231 (p53^R280K^) tumors by 89.4% (tumor volume) and 91.4% (tumor weight) at the end of the experiment (Fig. 10a, b). In contrast, PEPD siRNA had no effect on the growth of MDA-MB-231 (p53^KO^) tumors (Fig. 10c, d). However, PEPD siRNA also inhibited HCC70 (p53^R248Q^) tumors by 85.9% (tumor volume) and 88.8% (tumor weight) at the end of the experiment (Fig. 10e, f).

Because p53^R280K^ and p53^R248Q^ are contact mutants, we also investigated p53^R175H^, a conformation mutant. SK-BR-3 cells carry p53^R175H^ but failed to generate tumors in vivo. Since p53^R175H^ expressed transiently in MDA-MB-231 (p53^KO^) cells was reactivated by PEPD KD as described before, we generated MDA-MB-231 cells stably expressing p53^R175H^. We selected a p53^R175H^-expressing clone (clone #1), whose p53^R175H^ level is similar to that in SK-BR-3 cells (Supplementary Fig. 9a). PEPD siRNA strongly inhibited the growth of MDA-MB-231 (p53^R175H^) cells in vitro (Supplementary Fig. 9b). We inoculated MDA-MB-231 (p53^R175H^) cells to the mammary fat pads of female SCID mice, and tumors grew rapidly. When average tumor size reached about 130 mm^3^, intratumor injection of siRNA (10 pmol) was started, which was given once every 3 days. The experiment was stopped when average tumor size in the control reached about 550 mm^3^. Tumors were collected from the mice 2 days after the final treatment. Whereas tumors grew rapidly on scramble siRNA, PEPD siRNA inhibited tumor growth by 71.1% (tumor volume or tumor weight) at the end of the experiment (Fig. 10g, h).

Analysis of representative tumors showed that PEPD siRNA caused pronounced PEPD KD in all four types of tumors. In MDA-MB-231 (p53^R280K^) tumors, MDA-MB-231 (p53^R175H^) tumors, and HCC70 (p53^R248Q^) tumors, PEPD siRNA did not change total p53 level but strongly induced its acetylation at K373, upregulated p21, CD95 and BAK, downregulated BCL-2, and activated caspase 7, but in MDA-MD-231 (p53^KO^) tumors, PEPD siRNA had no effect on these proteins (Fig. 10i).

Notably, in a pair of isogenic tumors in mice derived from human colon cancer HCT116 cells (p53 or p53^KO^), PEPD KD by intratumor injection of siRNA inhibited the growth of p53 tumors by 79% with strong induction of p53 target proteins but had no effect on p53^KO^ tumors^7^. Thus, the in vivo tumor-suppressing activities of p53^R175H^, p53^R248Q^, and p53^R280K^ reactivated by PEPD KD are similar to that of p53 activated by PEPD KD.

## Discussion

In this study, we show a cellular mechanism that reactivates oncogenic p53 mutants (Fig. 10j). p53 mutants bind to PEPD in both nucleus and cytosol, with 40–47% of each mutant binding to 6–9% of PEPD. Interestingly, cellular concentrations of PEPD are 3.1–6.7-fold higher than that of p53 mutants. However, many proteins are known to bind to p53 mutants, some of which may interfere with PEPD binding. PEPD binds to PRD in p53 via its C-terminal sequence^7^. Because the PRD is rarely changed in p53 mutants, we speculate that PEPD may bind to a wide variety of p53 mutants. PEPD binding does not change the conformation of p53 mutants, but disrupting the binding via PEPD KD causes the mutants to undergo acetylation, mono-ubiquitination and phosphorylation, which cause their refolding and reactivation. The reactivated p53 mutants are almost indistinguishable functionally from activated p53. Our findings raise the question whether loss-of-function p53 mutants are also reactivated by PEPD KD. Several small molecule agents reactivate certain p53 mutants^31–33^. A peptide inhibitor of mutant p53 aggregation also rescues tumor-suppressing activities of some p53 mutants^34^. However, these external activators must bind to p53 mutants to reactivate them, whereas PEPD KD reactivates p53 mutants by freeing them from PEPD.

Why does PEPD bind to p53 mutants in the first place? Our results do not suggest that PEPD is required for the oncogenic functions of p53 mutants. It is possible that PEPD binds to p53 mutants simply because they carry the same PRD as does p53. Indeed, PEPD binds to p53 and p53 mutants with similar affinity. PEPD is important for p53 response to stress, as stress signals activate p53 in part by freeing it from PEPD^7^. An important question is whether stress signals may reactivate p53 mutants by freeing them from PEPD.

Approximately 50% of each p53 mutant in cells do not bind to PEPD and therefore are not reactivated by PEPD KD. This was shown clearly by p53^R175H^. PEPD KD causes refolding of about 40% of p53^R175H^ in SK-BR-3 cells, and only about 40% of p53^R175H^ initially bind to PEPD. Our analysis of caspase 3 activation also indicates presence of both reactivated and unreactivated p53 mutants in cells upon PEPD KD. PEPD KD activates caspase 3 in cells carrying p53 but fails to do so in cells carrying any p53 mutant, although caspases 8 and caspase 9, both of which activate caspase 3, are activated in all the cell lines. p53 mutants, such as p53^R175H^ and p53^R273H^, were shown to bind to caspase 3 to block its activation^35^. Thus, unreactivated p53 mutants likely block caspase 3 activation by their reactivated counterparts. p53 and p53 mutants are known to antagonize each other^36, 37^. Further study is needed to better understand the antagonism between p53 mutants reactivated by PEPD KD and their unreactivated counterparts. However, our study shows that reactivated p53 mutants determine cell fate, as reactivation of p53 mutants leads to cell death and tumor growth inhibition.

PEPD KD frees p53 mutants from PEPD for acetylation, and K373 acetylation is critical for their reactivation. K373 in p53 is a substrate of p300/CBP, and C646 which inhibits p300/CBP blocks the reactivation of p53 mutants by PEPD KD. This suggests the critical role of p300/CBP in reactivation of p53 mutants. However, it remains undetermined whether PEPD KD somehow activates p300/CBP or simply frees p53 mutants for modification by the enzyme. p53 mutants are enriched in the mitochondria upon PEPD KD. PEPD KD frees p53 mutants from PEPD for mono-ubiquitination by MDM2, and mono-ubiquitinated p53 mutants accumulate in mitochondria. Mono-ubiquitination of p53 is known to drive its nuclear export and mitochondrial translocation^18, 19^. Interestingly, our study shows that K373 acetylation is also required for mitochondria translocation of p53 mutants induced by PEPD KD. This raises the question whether K373 acetylation is a prerequisite for mono-ubiquitination of p53 mutants by MDM2. Multiple lysine residues in the C-terminal regulatory domain of p53 are substrates of MDM2, including K370, K372, K373, K381, K382, and K386^38^, but they may also be acetylated by p300/CBP^39^. The exact sites of mono-ubiquitination induced by PEPD KD remain undefined. Notably, despite PEPD KD, no p53 mutant shows acetylation at K370, and acetylation at K372, K382 or K386 is either unchanged or only increased in some p53 mutants, suggesting that some of these lysine residues may be mono-ubiquitinated upon PEPD KD. Mitochondrial increase in p53 was associated with corresponding decrease in its cytosolic and nuclear levels. Mitochondria increase in p53 mutants was also accompanied by their decrease in the cytosol, but their nuclear levels varied from no change (p53^R280K^ and p53^R175H^), increase (p53^R273H^), to decrease (p53^R248Q^). The exact reason for the variability is not known, but PEPD KD does not induce mono-ubiquitination of nuclear p53^R175H^.

PEPD KD also frees p53 mutants from PEPD for phosphorylation. The phosphorylation sites vary with p53 mutant, but the variability does not apparently impact their transcriptional activity, as the p53 mutants show similar induction of a p53 reporter in response to PEPD KD. However, only four phosphorylation sites in the transactivation domains are analyzed, and there are over 20 such sites in p53^40^. Analysis of other phosphorylation sites in p53 mutants may clarify whether there is a shared phosphorylation site and the role of phosphorylation in reactivation of p53 mutants by PEPD KD. Enzymes that catalyze p53 phosphorylation at various sites are known^39^, making it possible to address whether PEPD KD activates these enzymes or simply frees p53 for phosphorylation by them.

It remains unknown why p53 mutants freed from PEPD, not the p53 mutants unbound to PEPD, undergo posttranslational modifications that result in their refolding and reactivation. It is possible that PEPD binding to p53 mutants may recruit other proteins, such as p300/CBP, and these proteins may initiate posttranslational modifications of the mutants once they separate from PEPD. It should also be noted that previous genome-wide CRISPR screening did not identify PEPD as critical for cell survival^41, 42^. However, CRISPR-induced insertion/deletion have been shown to frequently provoke on-target mRNA mis-regulation, generating truncated or aberrant proteins^43–45^. The C-terminal fragment of PEPD (PEPD^265–492,493*aa*^) binds to the PRD in p53^7^. If similar PEPD fragments were generated in the CRISPR screening studies mentioned above, p53 and its mutants might not be perturbed and cells might continue to grow. PEPD is widely distributed^46^, and PEPD regulation of p53 mutants likely is wide-spread. Our study findings suggest that PEPD is a highly promising cancer therapeutic target and provide a strong scientific premise for seeking therapeutic strategies that target the specific interaction between PEPD and p53 mutants in cancer cells.

In conclusion, we have identified a cellular mechanism by which the tumor suppressor functions of oncogenic p53 mutants are restored, which is neither cell-specific nor mutant-specific. p53 mutants bind to PEPD, and disrupting their binding causes posttranslational modifications of the freed mutants, which drive their refolding and reactivation. The reactivated p53 mutants strongly inhibit cancer cell growth in vitro and in vivo.

## Methods

### Antibodies

Anti-acetyl-p53 (K120) (Cat# ab78316), anti-acetyl-p53 (K305) (Cat# ab109396), anti-acetyl-p53 (K386) (Cat# ab52172), and anti-PEPD (Cab# ab86507 and ab197890) were purchased from Abcam. Anti-acetyl-p53 (K372) (Cat# A96486) was purchased from Antibody.com. Anti-BAK (Cat# 3792), anti-BAX (Cat# 2772), anti-BCL-2 (Cat# 2870), anti-BCL-XL (Cat# 2764), anti-BID (Cat# 2002), anti-β-tubulin (cat#86298), anti-cleaved caspase 3 (Cat# 9661), anti-cleaved caspase 7 (Cat# 9491), anti-cleaved caspase 8 (Cat# 9496), anti-cleaved caspase 9 (Cat# 9501), anti-Cyto c (Cat# 4272), anti-EGFR (Cat# 2232), anti-EndoG (Cat# 4969), anti-MKK3 (Cat# 8535), anti-MYC (Cat# 9402), anti-p53 (Cat# 2524, 2527 and 18032), anti-phospho-p53 (S6) (Cat#9285), anti-phospho-p53 (S15) (Cat# 9284), anti-phospho-p53 (S20) (Cat# 9287), anti-phospho-p53 (S46) (Cat#2521), anti-p21 (Cat# 2946), and anti-VDAC (Cat# 4866) were purchased from Cell Signaling Technology. Anti-acetyl-p53 (K320) (Cat# 06-1283), anti-GAPDH (Cat# MAB374), PAb1620 (Cat# OP33-20UG), and PAb240 (Cat# OP29-100UG) were purchased from Millipore. Anti-AIF (Cat# sc-13116), anti-lamin B (Cat# sc-6216), anti-MDM2 (Cat# sc-965), anti-PUMA (sc-28226), and anti-ubiquitin (Cat# sc-8017) were purchased from Santa Cruz Biotechnology. Anti-acetyl-p53 (K319) (Cat# PA5-99334), anti-acetyl-p53 (K370) (Cat# MA5-32007), anti-acetyl-p53 (K373) (Cat# PA5-105109), anti-acetyl-p53 (K381) (Cat# PA5-105110), anti-acetyl-p53 (K382) (Cat# 710294), anti-CYPD (Cat# PA5-80923), and anti-6x-His tag (Cat# MA1-21315) were purchased from Thermo Fisher Scientific. Sheep anti-mouse IgG-HRP (Cat# NA931) and donkey anti-rabbit IgG-HRP (NA934) were purchased from GE Healthcare. Goat anti-rabbit IgG (Cat# 111-035-008) and goat anti-mouse IgG (Cat# 115-035-008) were purchased from Jackson ImmunoResearch Labs.

### Chemicals and enzymes

Bis(sulfosuccinimidyl) suberate (BS3) (Cat# 21580), BSA (Cat# 9048-46-8), C646 (Cat# 382113), RNase (Cat# AM2286), restriction enzyme Kpnl (Cat# FD0524), restriction enzyme Pmel (Cat# ER1342), and ProLong Gold Antifade Mountant with DAPI (Cat# P36941) were purchased from Thermo Fisher Scientific. Doxycycline hyclate (Dox) (Cat# D9891), EmbryoMax filtered light mineral oil (Cat# ES-005-C), phosphatase inhibitor cocktail 2 (Cat# P5726), phosphatase inhibitor cocktail 3 (Cat# P0044), phenylmethanesulfonyl fluoride (PMSF) (Cat# 329-98-6), propidium iodide (PI) (Cat# P-4170), puromycin (Cat# P8833), and trypan blue (Cat# T8154) were purchased from Sigma-Aldrich. Cell lysis buffer (Cat# 9803), TMB substrate (Cat# 7004), and STOP solution (Cat# 7002) were purchased from Cell Signaling Technology. Protease inhibitor cocktail (Cat# 11-836-153-001) was purchased from Roche Applied Science. Ubiquitin aldehyde (Cat# BML-UW8450) was purchased from Enzo Life Sciences. Phostag acrylamide (Cat# 304-93562) was purchased from Wako. Matrigel (Cat# 356237) was purchased from Corning.

### Assay kits

Lipofectamine 3000 (Cat# L3000001), Lipofectamine RNAiMAX (Cat# 13778-075), the High-Capacity cDNA Reverse Transcription Kit (Cat# 4368814), the NE-PER Nuclear and Cytoplasmic Extraction Kit (Cat# 78833), the MagMAX DNA Multi-Sample Kit (Cat# 4413020), and the Silver Staining Kit (Cat# 24612) were purchased from Thermo Fisher Scientific. The Dual-Luciferase Reporter Assay Kit (Cat# E1910), CellTiter-Glo 2.0 Cell Viability Assay (Cat# G9241), and FuGENE HD (Cat# E231A) were purchased from Promega. Luminata Classico (Cat# WBLUC0500), and Luminata Crescendo (Cat# WBLUR0100) were purchased from Millipore. The Clarity Western ECL Substrate (Cat# 1705060), the Clarity Max Western ECL Substrate (Cat# 1705062), and the Universal SYBR Green Master Mix (Cat# 172-5122) were purchased from Bio-Rad. The MinElute PCR Purification Kit (Cat# 28004), the RNeasy Plus Mini Kit (Cat# 74134), and the QIAquick Gel Extraction Kit (Cat# 28704) were purchased from Qiagen. The Immunoprecipitation Starter Pack was purchased from GE Healthcare (Cat# 17-6002-35). The JC-1 Mitochondria Membrane Potential Assay Kit was purchased from G-Bioscience (Cat# 786-1322). The EpiQuik Chromatin Immunoprecipitation Kit was purchased from Epigentek (Cat# P-2002). The Bicinchoninic Acid Assay Kit (Reagent A Cat# 23228, and Reagent B Cat#1859078) was purchased from Pierce. The QuikChange Lightning Site-Directed Mutagenesis Kit was purchased from Agilent (Cat# 210518). The TUNEL Andy Fluor Apoptosis Detection Kit was purchased from ABP Biosciences (Cat# A051).

### Plasmids

pBAD/TOPO-human PEPD-His and pET15b-His-human p53^mPRD^ were generated previously in our lab^7,47^. PG13-Luc (Cat# 16442), MG15-Luc (Cat# 16443), and pET15b-His-human p53 (Cat# 24859) were purchased from Addgene. pRL-TK (Cat# E2241) was purchased from Promega.

pET15b-His-human p53 was used as a template to generate plasmids for bacterial expression of p53 mutants, including pET15b-His-human p53^R175H^, pET15b-His-human p53^R248Q^, pET15b-His-human p53^R273H^, and pET15b-His-human p53^R280K^. pCMV6-A-human p53-puro, which was generated previously in our lab^7^, was used as a template to generate plasmids for mammalian expression of p53 mutants, including pCMV6-A-human p53^R175H^-puro, pCMV6-A-human p53^R248Q^-puro, pCMV6-A-human p53^R273H^-puro, and pCMV6-A-human p53^R280K^-puro. pCMV6-A-human p53^R175H^-puro, pCMV6-A-human p53^R248Q^-puro, pCMV6-A-human p53^R273H^-puro, and pCMV6-A-human p53^R280K^-puro were used as templates to generate double mutants, including pCMV6-A-human p53^R175H/K373R^-puro, pCMV6-A-human p53^R248Q/K373R^-puro, pCMV6-A-human p53^R273H/K373R^-puro, and pCMV6-A-human p53^R280K/K373R^-puro. pCMV6-XL5-human PEPD (Origene, Cat# SC119982) was used to generate pCMV6-XL5-human PEPD^G278D^ for mammalian expression of PEPD^G278D^ without 3′-UTR. All mutations were generated using the QuikChange Lightning Site-Directed Mutagenesis Kit. All the primers were purchased from IDT, including primers for generating PEPD^G278D^ (forward: 5′-TGCCTGTTCGACATGGACGGTGAGTATTACTGC-3′; reverse: 5′-GCAGTAATACTCACCGTCCATGTCGAACAGGCA-3′), primers for generating PEPD^G278D^ without 3′-UTR (forward: 5′-TCTGGCCCCAAGTAGCTCTAGATTGCGGCC-3′; reverse: 5′-GGCCGCAATCTAGAGCTACTTGGGGCCAGA-3′); primers for generating p53^R175H^ (forward: 5′-GAGGTTGTGAGGCACTGCCCCCACCAT-3′; reverse: 5′-ATGGTGGGGGCAGTGCCTCACAACCTC-3′), primers for generating p53^R248Q^ (forward: 5′-GGCGGCATGAACCAGAGGCCCATCCTC-3′; reverse: 5′-GAGGATGGGCCTCTGGTTCATGCCGCC-3′), primers for generating p53^R273H^ (forward: 5′-GAACAGCTTTGAGTGCATGTTTGTGCCTGTCCTG-3′; reverse: 5′-CAGGACAGGCACAAACATGCACCTCAAAGCTGTTC-3′), primers for generating p53^R280K^ (forward: 5′-TGTGCCTGTCCTGGGAAAGACCGGCG-3′; reverse: 5′-CGCCGGTCTTTCCCAGGACAGGCACA-3′), and primers for generating p53^K373R^ (forward: 5′-CCAGCCACCTGAAGTCCAAAAGGGGTCAGTCTA-3′; reverse: 5′-TAGACTGACCCCTTTTGGACTTCAGGTGGCTGG-3′). To generate Tet-inducible mammalian PEPD expression (pTet-on-PEPD), the full-length coding sequence of human PEPD was subcloned from pCMV6-AC-PEPD (Origene, Cat# SC322376) into AAVS1_Puro_Tet3G_3xFlag_Twin_Strep (Addgene, Cat# 92099), to generate AAVS1_Puro_Tet3G_PEPD_Twin_Strep. Both the donor plasmid and the destination plasmid were cut with restriction enzymes KpnI and PmeI. The PEPD fragment from pCMV6-AC-PEPD and the linearized destination plasmid were purified using the QIAquick Gel Extraction Kit and then ligated at 15 °C overnight. All constructs were confirmed by DNA sequence analysis.

### *TP53* mutation analysis

Total genomic DNA was extracted from cells using the MagMAX DNA Multi-Sample Kit. The *TP53* sequences encompassing R175, R248, R273 and R280 were amplified by PCR. The PCR condition is as follows: 95 °C for 3 min, 35 cycles at 95 °C for 30 s (denaturation), 65 °C for 30 s (annealing), and 72 °C for 1.7 min (extension), with the final extension performed at 72 °C for 5 min. The primers used for the reaction are as follows: 5′-gTACTCCCCTGCCCTCAACAAGAT-3′ (forward) and 5′-ggaaagaggcaaggaaaggtgata-3′ (reverse). The uppercase letters denote exon sequence, and the lowercase letters denote intron sequence. The primers were purchased from IDT. All PCR reaction products were purified using the MinElute PCR Purification Kit and were subjected to DNA sequencing. The afore-mentioned forward primer was used for sequencing across R175, and the reverse primer was used for sequencing across R248, R273, and R280.

### Preparation of recombinant proteins

Recombinant human PEPD, human p53 and its mutants were expressed in BL21-AI One Shot Chemically Competent *E. coli* (human p53 and its mutants) or One Shot TOP10 Chemically Competent *E. coli* (human PEPD). BL21-AI One Shot Chemically Competent *E. coli* (Cat# C607003) and One Shot TOP10 Chemically Competent *E. coli* (Cat# C404010) were purchased from Thermo Fisher Scientific. p53 and its mutants are N-terminal His tagged, whereas PEPD is C-terminal His tagged. The bacterial expression constructs used include pET15b-His-human p53, pET15b-His-human p53^R175H^, pET15b-His-human p53^R248Q^, pET15b-His-human p53^R273H^, pET15b-His-human p53^R280K^, pET15b-His-human p53^mPRD^, and pBAD/TOPO-human PEPD-His. For protein expression, the *E. coli* colonies transduced with the plasmids were grown in LB Broth Miller from Novagen (Cat# 71753-5) overnight at 37 °C with shaking. Once the cells grew to OD_600_ ≈ 1, arabinose was added to the LB broth (0.2% final concentration), and the cells were grown at 37 °C with shaking for another 3 h. Cells were harvested by centrifugation, resuspended in cell lysis buffer (50 mM NaH_2_PO_4_, 300 mM NaCl, 10 mM imidazole, pH 8.0) at 3 ml per g of cells, which was sonicated on ice using six 10 s bursts with a 10 s cooling period between each burst (Branson Sonifier 450, VWR Scientific). The lysates were centrifuged at 10,000 × *g* for 30 min at 4 °C to pellet and remove the cellular debris. The cleared supernatant was mixed with 50% Ni-NTA slurry from Qiagen (Cat# 30210) at 4:1 ratio by volume, which was gently shaken at 4 °C for 60 min. The mixture was then loaded onto a polypropylene column from Qiagen (Cat# 34964), which was washed twice with wash buffer (50 mM NaH_2_PO_4_, 300 mM NaCl, 20 mM imidazole, pH 8.0). The recombinant protein was eluted with elution buffer (50 mM NaH_2_PO_4_, 300 mM NaCl, 250 mM imidazole, pH 8.0) and was concentrated in phosphate buffered saline (PBS) using the Ultracel YM-10 Centricon (Cat# MRCPRT010) or YM-30 Centricon (Cat# MRCF0R030) from Millipore. The purity of each protein was examined by sodium dodecyl sulfate polyacrylamide gel electrophoresis (SDS-PAGE) and silver staining. Protein concentrations were measured by the Bicinchoninic Acid Assay Kit.

### Measurement of PEPD binding to p53 and its mutants by ELISA

ELISA plate wells were coated with a p53 antibody (Cell Signaling Technology, Cat# 2527) by incubating 1 μg/100 μl of the antibody per well overnight at 4 °C. After washing the wells with PBS with 0.05% Tween 20 (PBST), residual protein binding sites were blocked by incubation for 2 h at room temperature (RT) with 300 μl/well of 1% bovine serum albumin (BSA) in PBS. Sixty microliters of serially diluted recombinant human PEPD was added to each well (final concentration: 10, 40, 80, 160, 640, 1280, and 2560 nM), followed by addition of 60 μl of recombinant human p53 or p53 mutant (final concentration: 250 nM) to each PEPD-containing well and incubation at 37 °C for 2 h. Each well was washed with PBST, incubated with 12.5 ng detection antibody in 100 μl PBS (anti-PEPD, Sigma-Aldrich, Cat# WH0005184M1) for 2 h at RT, washed again with PBST, incubated with 100 μl of a HRP-conjugated secondary antibody (GE Healthcare, Cat# NA931, 1:8,000 dilution) for 1 h at RT, washed again with PBST, and incubated with 100 μl TMB substrate (3,3′,5,5′-Tetramethylbenzidine). After adequate color development, 100 μl/well of STOP solution was added, followed by absorbance reading at 450 nm by the Synergy 2 Multi-Mode Microplate Reader from BioTek.

### Analysis of the PEPD-p53 complex and PEPD-p53^R175H^ complex

Recombinant human p53 or p53^R175H^ (114 nM) were incubated with recombinant human PEPD (801 nM) in 100 μl PBS (pH 7.4) for 15 min at RT, which was then incubated with 2 mM BS3, a cross-linker, for 30 min at RT. The cross-linking reaction was terminated by adding 1M Tris-HCl (pH 7.5) (final concentration: 20 mM) to each mixture, which was incubated at RT for 15 min. The samples were then analyzed by WB for PEPD, p53 and p53^R175H^, using 5% SDS-PAGE.

### Cell lines and cell culture

MDA-MB-231 (Cat# HTB-26), MDA-MB-468 (Cat# HTB-132), SK-BR-3 (Cat# HTB-30), HCC70 (Cat# CRL-2315), and MCF-7 (Cat# HTB-22) were from American Type Culture Collection. CAL-51 (Cat# ACC-302) was from Deutsche Sammlung von Mikroorganismen und Zellkulturen. MDA-MB-231 (p53^KO^) cells were generated from MDA-MB-231 cells by CRISPR-Cas9^13^. MDA-MB-231^DKO^ cells were generated from MDA-MB-231 (p53^KO^) cells by CRISPR-Cas9, and MDA-MB-231 (p53^R175H^) cells were generated by transfecting pCMV6-A-human p53^R175H^-puro into MDA-MB-231 (p53^KO^) cells and selection under puromycin, as described below. The cell lines were authenticated using short tandem repeat analysis and tested negative for mycoplasma contamination, and their *TP53* genotypes were verified by Sanger sequencing. MDA-MB-231 cells, MDA-MB-231 (p53^KO^) cells, MDA-MB-231 (p53^R175H^) cells, MDA-MB-468 cells, MCF-7 cells, and CAL-51 cells were cultured in high-glucose Dulbecco’s modified Eagle medium (DMEM) supplemented with 10% fetal bovine serum (FBS). SK-BR-3 cells were cultured in McCoy’s 5A medium supplemented with 10% FBS. HCC70 cells were cultured in RPMI-1640 medium supplemented with 10% FBS. MDA-MB-231^DKO^ cells were cultured in high-glucose DMEM supplemented with 10% FBS or high-glucose DMEM supplemented with 10% Tet System Approved FBS. All cells were cultured in humidified incubators with 5% CO_2_ at 37 °C. High-glucose DMEM (Cat# 10-013-CV), McCoy’s 5A Medium (Cat# 10-050-CV) and RPMI-1640 Medium (Cat# 10-040-CV) were purchased from Corning Cellgro. FBS (Cat# 10437) was purchased from Gibco. Tet System Approved FBS (Cat# 631105) was purchased from Takara.

### Generating MDA-MB-231^DKO^ cells

MDA-MB-231 (p53^KO^) cells were used to generate MDA-MB-231^DKO^ (p53^KO^/PEPD^KO^) by CRISPR-Cas9. Cas9 nuclease expression vector (pST1374-N-NLS-Flag-Cas9-EGFP) and PEPD sgRNA vectors (pGL3-PEPD-sgRNA1, and pGL3-PEPD-sgRNA6) were purchased from Celltechgen. The PEPD sgRNA sequences are as follows: gccgctcacacaggcgctgc (sgRNA1), and gccacctggatgggaaagta (sgRNA6). Cells were grown in -well plates (1 × 10^5^ cells per well with 2 ml medium) and 24 h later cotransfected with 1 μg of pGL3-PEPD-sgRNA1, 1 μg of pGL3-PEPD-sgRNA6, and 2 μg of pST1374-N-NLS-Flag-Cas9-EGFP using Lipofectamine 3000. Three days after transfection with the plasmids, the cells were seeded into a 96-well plate at one cell per well using flow cytometry-based sorting, and the cells were grown in DMEM with 10% FBS and 1% penicillin and streptomycin until colony formation (about 3 weeks). Each colony was trypsinized and split into two replica plates. Once cells in each well grew near confluency, one plate of cells were subjected to next generation sequencing to identify cells with *PEPD* gene mutation. The second identical 96-well plate was preserved for future expansion of cells. To this end, cells in each well were trypsinized with 25 μl 1x trypsin, then overlaid with 25 μl culture medium (DMEM with 15% FBS and 1% penicillin and streptomycin), 50 μl 2x freezing medium (DMEM with 20% FBS and 20% dimethyl sulfoxide), and 50 μl EmbryoMax filtered light mineral oil. The plate was then sealed in a Kapak bag and stored at −80 °C. Once a positive PEPD KO colony was identified by next generation sequencing, the same cells in the frozen plate was thawed and expanded, which were then subjected to WB to confirm PEPD KO.

### Generating MDA-MB-231 cells stably expressing p53^R175H^

MDA-MB-231 (p53^KO^) cells growing in 12-well plates were transfected with pCMV6-A-human p53^R175H^-puro (1 μg DNA/well) using Lipofectamine 3000. The cells were treated with puromycin (2 μg/ml) at 48 h after gene transfection. The culture medium containing puromycin was changed twice weekly. After 1 week of puromycin treatment, the cells were sub-cultured to 96-well plates (1 cell/well) and about 3 weeks later, proliferating cells in select wells were sub-cultured and propagated in 24-well plates and analyzed by WB for transgene expression, to identify a desired stable clone, which was further propagated in 10-cm dishes.

### Preparation of WCL, subcellular fractions, and tumor homogenates

Cells were washed with PBS, mixed with 1x cell lysis buffer supplemented with 2 mM PMSF and a protease inhibitor cocktail at ~1 × 10^6^ cells per 100 μl lysis buffer, incubated on ice for 10 min, sonicated at 0–4 °C using the Branson Model 450 Sonifier to enhance cell lysis, and centrifuged at 13,000 × *g* for 10 min at 4 °C. The supernatant fraction was collected as WCL. To prepare cell lysates minus mitochondria as well as mitochondria, cells were washed with PBS, suspended in isotonic homogenization buffer (25 mM sucrose, 10 mM KCl, 1.5 mM MgCl_2_, 1 mM NaEDTA, 1 mM NaEGTA, 1 mM dithiothreitol, 1 mM PMSF, 0.1 mM Tris-HCl, pH 7.4, a protease inhibitor cocktail, and phosphatase inhibitor cocktail 2 and cocktail 3) at 10 × 10^6^ cells per 0.75 ml buffer, incubated on ice for 10 min, and then homogenized in a Dounce homogenizer. The homogenates were centrifuged at 400 × *g* for 5 min at 4 °C. The precipitates were resuspended in 0.1 ml 1x cell lysis buffer per sample (sample A), whereas the supernatant fraction was further centrifuged at 12,000 × *g* for 20 min at 4 °C to obtain cytosolic fraction (supernatant) and mitochondria fraction (pellet). When the cytosolic fraction was combined with sample A mentioned above, the mixture is designated as cell lysates minus mitochondria. The mitochondria pellets were washed with homogenization buffer and suspended in 1x lysis buffer supplemented with 2 mM PMSF, a protease inhibitor cocktail, and phosphatase inhibitor cocktail 2 and cocktail 3. Nuclear fraction was prepared using the NE-PER Nuclear and Cytoplasmic Extraction Kit supplemented with 2 mM PMSF, a protease inhibitor cocktail, and phosphatase inhibitor cocktail 2 and cocktail 3.

Tumor samples were mixed with RIPA buffer (25 mM Tris-HCl, pH 7.6, 150 mM NaCl, 1% Nonidet P-40, 1% sodium deoxycholate, and 0.1% SDS), which was supplemented with 2 mM PMSF, a protease inhibitor cocktail, and phosphatase inhibitor cocktail 2 and cocktail 3 at 14.3 μl buffer per mg tissue, and homogenized in a Dounce homogenizer. The homogenates were cleared by centrifugation at 13,000 × *g* for 15 min at 4 °C.

### Measurement of PEPD binding to p53 in cells

PEPD binding to p53 and p53 mutants in WCL, cytosol and nuclear extract were measured by IP followed by WB as well as ELISA. For IP, WCL (0.5 mg protein per sample), cytosolic fraction (0.2 mg protein per sample) or nuclear extract (0.2 mg protein per sample) was incubated overnight at 4 °C with a p53 antibody (Cell Signaling Technology, Cat# 2527) or a PEPD antibody (Abcam, Cat# ab86507), with either 1 μg antibody per sample (0.5 mg protein in 500 μl PBS) or 0.4 μg antibody per sample (0.2 mg protein in 200 μl PBS), and was then incubated with 30 μl Protein G Sepharose (2 mg/ml) for 1 h at RT. The precipitates were washed with IP buffer and analyzed along with an input (10–20% of each sample used for IP) by WB for p53, its mutant or PEPD. The intensities of the WB bands of interest were quantified by ImageJ. Select supernatant samples were also analyzed by WB to confirm full pull-down of p53, its mutant or PEPD.

Supernatants from IP of WCL samples were measured by ELISA for PEPD and p53 that are not bound to each other. ELISA plates wells were coated with a p53 antibody (Cell Signaling Technology, Cat# 18032) or a PEPD antibody (Sigma-Aldrich, Cat# WH0005185M1) by incubating the antibody (1 μg in 100 μl) in each well overnight at 4 °C, and then washing each well with PBST. Residual protein binding sites were blocked by incubation for 2 h at RT with 300 μl/well of 1% BSA in PBS. Assay samples (100 μl supernatants diluted in PBS) or standards (100 μl recombinant p53 or PEPD in PBS at 0, 3.6, 7.8, 15.6, 62.5, and 250 pM) were added to each well and incubated at 37 °C for 2 h. Each well was washed with PBST, incubated with a detection antibody (8–9 ng antibody) in 100 μl PBS for 2 h at RT, either a p53 antibody (Cell Signaling Technology, Cat# 2527) or a PEPD antibody (Abcam, Cat# ab197890), washed again with PBST, incubated with 100 μl of a HRP-conjugated secondary antibody (Jackson ImmunoResearch Labs, Cat# 111-035-008) at 1:10,000 dilution for 1 h at RT, and washed further with PBST, and then incubated with 100 μl of TMB substrate. After adequate color development, 100 μl/well of STOP solution was added, and absorbance of each well at 450 nm was recorded by the Synergy 2 Multi-Mode Microplate Reader. Concentrations of PEPD, p53 and p53 mutant in a sample were calculated based on calibration curves established using recombinant PEPD and p53.

### Gene transfection and Dox treatment

Plasmid transfection was performed using FuGENE HD or Lipofectamine 3000. Cells were seeded in 12-well plates (6-8 × 10^4^ cells/well with 1 ml medium) or 6-well plates (8–12 × 10^4^ cells/well with 2 ml medium) and 24 h later transfected with 1 or 2 μg plasmid per well. For transfection of plasmids including PG13-Luc, MG15-Luc and pRL-TK, cells were seeded in 6-well plates (1–2 × 10^5^ cells/well with 2 ml medium) and 24 h later transfected with 1 μg PG13-Luc or MG15-Luc along with 0.05 μg pRL-TK per well. In cells transfected with Tet-inducible PEPD (Tet-on-PEPD) along with a p53 mutant, cells were seeded in 12-well plates at 1.2 × 10^5^ cells per well with 1 ml medium and 24 h later transfected with each plasmid (1 µg DNA per well) and immediately treated with or without Dox (50 ng/ml). In another experiment, cells were seeded in 6-well plates at 1.2 × 10^5^ cells per well with 2 ml medium overnight and 24 h later transfected with Tet-on-PEPD and a p53 mutant (1 μg each plasmid per well) with Dox (50 ng/ml) added to the culture medium immediately after transfection. The cells were washed with culture medium 24 h after gene transfection and cultured in fresh medium (2 ml/well) with or without Dox (50 ng/ml).

### siRNA transfection and other treatments

Cells were seeded in 10-cm dish (1–3 × 10^6^ cells with 10 ml medium), 6-well plates (8–40 × 10^4^ cells/well with 2 ml medium), 12-well plates (6–8 × 10^4^ cells/well with 1 ml medium), or 96-well plates (1.5–2 × 10^4^ cells/well with 0.1 ml medium), and 24 h later, transfected with scramble siRNA or PEPD siRNA, or first transfected with scramble siRNA or MDM2 siRNA and 24 later transfected with scramble siRNA or PEPD siRNA. In some experiments, scramble siRNA was replaced by C911 control siRNA. Each siRNA was transfected at 10 nM using Lipofectamine RNAiMAX. In cells transfected with a plasmid(s), siRNA transfection was performed 24 h after plasmid transfection. In experiments where cells were transfected with siRNA and also treated with C646, C646 (8 μM) or solvent was added to the culture medium immediately after siRNA transfection. In experiments where cells were transfected with siRNA and also treated with ubiquitin aldehyde, ubiquitin aldehyde (100 μM) or solvent was added to the medium during the final 4 h of culture. The siRNAs were purchased from Origene or ITD, including nonspecific scrambled siRNA (rCrGrUrUrArArUrCrGrCrGrUrArUrArArUrArCrGrCrGrUAT; Cat# SR30004, Origene), Control siRNA (C911-PEPD) (rGrCrArUrUrUrGrArArGrUrGrArCrCrArArArCrArGrUrGCT; Custom-made, ITD), PEPD siRNA #1 (rGrCrArUrUrUrGrArUrCrArGrArCrCrArArArCrArGrUrGCT; Cat# SR303443D, Origene), PEPD siRNA#2 (rGrGrCrCrGrUrCrUrArUrGrArGrGrCrArGrUrGrCrUrGrCGG; Cat# SR303443E, Origene), and MDM2 siRNA (rCrCrCrUrArGrGrArArUrUrUrArGrArCrArArCrCrU rGrAAA; Cat# SR302849A, Origene).

### RT-PCR

Total RNA was isolated using the RNeasy Plus Mini Kit, and 500 ng RNA per sample was reverse transcribed to cDNA in 25 μl reaction using the High-Capacity cDNA Reverse Transcription Kit. The RT reaction was performed at 25 °C for 10 min, followed by heating at 48 °C for 30 min, and then 95 °C for 5 min. The primers for human PEPD and GAPDH were purchased from IDT. The PEPD primers are as follows: 5′-CTGCAGGGCGGGGAGGAGAC-3′ (forward), and 5′-CGCCCCGGGAGTAGCAGTAGTG-3′ (reverse). The GAPDH primers are as follows: 5′-CCAGGGCTGCTTTTAACTC-3′ (forward), 5′-GCTCCCCCCTGCAAATGA-3′ (reverse). Each PCR amplification was carried out in 20 μl volume, containing 10 μl 2x GoTaq Master Mix (Promega, Cat# M7122), 0.5–1 μl of the reverse-transcribed mixture (cDNA), and 0.25 μM each of specific forward and reverse primers. The PCR condition for PEPD is as follows: 94 °C for 3 min, 35 cycles at 95 °C (denaturation) for 30 s, 61 °C for 30 s (annealing), and 72 °C for 35 s (extension), with the final extension performed at 72 °C for 5 min. The PCR condition for GAPDH is as follows: 94 °C for 3 min, 25 cycles at 94 °C (denaturation) for 30 s, 60 °C for 30 s (annealing), and 72 °C for 30 s (extension), with the final extension performed at 72 °C for 5 min. The PCR products were analyzed by electrophoresis with 1% agarose gel, stained by ethidium bromide, and visualized under UV light.

### Cell cycle analysis

Cells were cultured in 6-well plates at 2 × 10^5^ cells per well, including MDA-MB-231 cells, MDA-MB-231 (p53^KO^) cells, and CAL-51 cells, or 4 × 10^5^ SK-BR-3 cells per well in 2 ml medium for 24 h and then treated with scramble siRNA or PEPD siRNA (10 nM) for 48 h. The cells were trypsinized, washed twice with ice-cold PBS, and pelleted by centrifugation at 2000 rpm for 5 min at 4 °C. The cells were fixed in ice-cold 70% ethanol for at least 30 min at 4 °C. After removing the ethanol by centrifugation and washing the cells twice with ice-cold PBS, the cells from each well were resuspended in 500 μl PI staining buffer containing 20 μg/ml RNase and 50 μg/ml PI, and incubated at RT in the dark for 30 min. The stained cells were analyzed by a flow cytometer (BD LSRFortessa). Cell cycle distribution was modeled using the ModFit LT software. Fifty thousand cells per sample were analyzed.

### Measurement of cell survival

Cell survival was measured by trypan blue exclusion assay or CellTiter-Glo cell viability assay. For the trypan blue exclusion assay, cells after experimental treatments were trypsinized and suspended in fresh culture medium. Ten microliters cell suspension (about 550 cells) was mixed with 10 μl of 0.4% trypan blue solution, which was loaded onto a hemocytometer, and viable cells (unstained cells) were counted under an inverted microscope. Approximately 1500–2000 cells were counted per sample. For the CellTiter-Glo assay, cells were seeded in 96-well plates with 100 μl medium per well (5 × 10^3^ CAL-51 cells, 6 × 10^3^ MDA-MB-231 cells, 1 × 10^4^ HCC70 cells, or 1.2 × 10^4^ SK-BR-3 cells per well) and after overnight culture, transfected with siRNA as described before. The CellTiter-Glo reagent (100 μl) was added to each well at 72 h after siRNA transfection and after incubation for 10 min at RT, cell viability in each well was determined by using a Veritas microplate luminometer (Turner Biosystems).

### WB, phostag WB, and IP

For WB, each sample was resolved by SDS-PAGE (8–12.5%). Proteins were transferred to polyvinylidene fluoride membrane, probed with specific antibodies, and detected using either Luminata Classico, Luminata Crescendo, Clarity Western ECL Substrate, or Clarity Max Western ECL Substrate. Certain WB bands were quantified by ImageJ. For phostag WB, MnCl_2_ (100 μM final concentration) and phostag acrylamide (20 μM final concentration) was added to the regular resolving gel. Notably, phostag provides phosphate affinity SDS-PAGE, generating mobility shift of phosphorylated proteins. For IP, WCL (0.5 mg protein per sample), cytosolic fraction (0.2 mg protein per sample), nuclear extract (0.2 mg protein per sample), or mitochondria (0.1 mg protein per sample) was incubated with a specific antibody (0.8 to 2 μg, depending on the antibody) in 500 μl PBS overnight at 4 °C, followed by incubation with 30 μl Protein A Sepharose or Protein G Sepharose (2 mg/ml) for 1 h at RT. Both Protein A Sepharose and Protein G Sepharose are components of the Immunoprecipitation Starter Pack. The immunoprecipitates were washed three times with IP buffer before WB analysis.

### Luciferase activity assay

Luciferase activity in WCL was measured by the Dual-Luciferase Reporter Assay Kit. Luminescence was measured by the Modulus Luminometer 9200-001 (Turner Biosystems).

### Mitochondrial membrane potential assay

MMP was determined by the JC-1 Mitochondria Membrane Potential Assay Kit. Cells were seeded in a 96-well black microtiter plate at 1.5–2 × 10^4^ cells per well in 100 μl cell culture medium overnight and then treated with siRNA as described before. The culture medium in each well was replaced with 100 μl JC-1 working solution (2 μM JC-1) per well, and the plate was incubated for 30 min at RT. The JC-1 solution in each well was then aspirated and replaced with 100 μl of prewarmed 1x MMP Assay Buffer. Fluorescence intensity in each well was immediately measured by the Synergy 2 Multi-Mode Microplate Reader, using excitation/emission at 535 nm/595 nm for JC-1 aggregates and excitation/emission at 485 nm/535 nm for JC-1 monomers.

### ChIP-qPCR

ChIP assay was performed using the EpiQuik Chromatin Immunoprecipitation Kit. Cells growing in 10 cm dishes, after siRNA treatment, were trypsinized and pelleted by centrifugation (1500 rpm, 5 min). Each pellet (~2 × 10^6^ cells) was resuspended in 10 ml PBS, pelleted by centrifugation (1500 rpm, 5 min), mixed with 9 ml culture medium containing 1% formaldehyde, incubated at RT for 10 min, mixed with 1 ml PBS containing 1.25 M glycine, pelleted by centrifugation (1500 rpm, 5 min), resuspended in ice-cold PBS (10 ml), and pelleted by centrifugation (1500 rpm, 5 min). Each pellet was resuspended in pre-lysis buffer (200 μl per 1 × 10^6^ cells), incubated on ice for 10 min, vortexed vigorously for 10 s, pelleted by centrifugation (1500 rpm, 5 min), resuspended in lysis buffer (100 μl per 1 × 10^6^ cells), and incubated on ice for 10 min with occasional vortexing. DNA was sheared by sonication using a Diagenode Bioruptor Sonicator at 4 °C. Cell debris were pelleted by centrifuge (14,000 rpm, 10 min), and the supernatant (100 μl) was added to a microwell and incubated at RT for 90 min to capture the DNA-p53 mutant complex. Each microwell was precoated with a p53 antibody by incubating with 2.5 μg p53 antibody (Cell Signaling Technology, Cat# 2524) in 100 μl of antibody buffer at RT for 90 min, followed by wash with antibody buffer. DNA was released from the antibody-captured protein-DNA complex by washing each well with wash buffer and then Tris-EDTA (TE) buffer (pH 8.0), adding 40 μl of DNA release buffer to each well and incubating at 65 °C for 15 min, adding 40 μl of reverse buffer to each well and incubating at 65 °C for 90 min, and finally adding 150 μl of binding buffer to each well and transferring the entire solution from each well to a spin column for DNA elution. Each column loaded with the sample was centrifuged at 12,000 rpm for 15 s and washed twice by adding 200 μl 90% ethanol each time and centrifuging at 12,000 rpm for 20 and 35 s. DNA in each column was eluted by adding 20 μl elution buffer and centrifuging at 12,000 rpm for 20 s. Promoter occupancy by p53 was analyzed by qPCR, which was performed using the Universal SYBR Green Master Mix in a 10 μ reaction, containing 0.4 μl forward primer (400 nM), 0.4 μl reverse primer (400 nM), 5 μl SYBR Green Supermix (2x), 1 μl DNA (20 ng/ml), and 3.2 μl H_2_O, in a CFX96 qPCR instrument (Bio-Rad). The qPCR condition is as follows: 95 °C for 10 min; 95 °C for 15 s, followed by 60 °C for 45 s (35 cycles). All experimental and control groups were performed in triplicates. The primers for *CDKN1A* and *BBC3* gene promoters were purchased from IDT. *CDKN1A* primers are as follows: 5′-CAGGCTGTGGCTCTGATTGG-3′ (forward), and 5′-CCTCACCTGAAAACAGGCAGC-3′ (reverse). *BBC3* primers as follows: 5′-GCGAGACTGTGGCCTTGTGT-3′ (forward), and 5′-CGTTCCAGGGTCCACAAAGTC-3′ (reverse). The input used in qPCR was 2.5% of each sample used in the pull-down.

### TUNEL assay

Apoptotic cells were detected using the TUNEL Andy Fluor Apoptosis Detection Kit. Briefly, CAL-51 cells, SK-BR-3 cells, MDA-MB-231 cells, and MDA-MB-231 (p53^KO^) cells were grown in 8-well chamber slides (4 × 10^4^ cells/well with 0.3 ml medium) overnight, followed by treatment with scramble siRNA or PEPD siRNA (10 nM) for 48 h. The cells were then washed with ice-cold PBS, fixed with 4% paraformaldehyde in PBS for 30 min at 37 °C, washed again with ice-cold PBS, and permeabilized with 0.2% Triton X-100 in PBS for 30 min at RT. The cells were then incubated with the TdT reaction cocktail for 60 min at 37 °C (protected from light), washed with 400 μl of 3% BSA in PBS (three times, each time for 5 min), and then incubated with the Alexa Fluor 594-Streptavidin staining solution (150 μl) for 30 min at RT. The cells were washed again with 3% BSA in PBS (three times, each time for 5 min) and counterstained with ProLong Gold Antifade Mountant with DAPI. The cells were then examined using the Keyence BZ-X710 All-in-One Fluorescence Microscope. Images were organized using ImageJ. Five hundred cells per sample were counted.

### Mouse study

SCID mice (C.B-17 SCID) were bred by the Laboratory Animal Shared Resource in our institute. All experiments were approved by our Institutional Animal Care and Use Committee. We established orthotopic breast tumors by inoculating MDA-MB-231 (p53^R280K^) cells, MDA-MB-231 (p53^R175H^) cells, MDA-MB-231 (p53^KO^) cells, or HCC70 (p53^R248Q^) cells to the mammary fat pads of 6–7 weeks old female SCID mice. Cells were inoculated to each site in 0.1 ml volume containing 1 × 10^6^ MDA-MB-231 (p53^R280K^) cells, 1.5 × 10^6^ MDA-MB-231 (p53^R175H^) cells, 2 × 10^6^ MDA-MB-231 (p53^KO^), or 2 × 10^6^ HCC70 (p53^R248Q^) cells. MDA-MB-231 cells with different p53 genotype were suspended in serum-free high-glucose DMEM, whereas HCC70 (p53^R248Q^) cells were suspended in 50% serum-free RPMI-1640 medium and 50% Matrigel. Tumor-bearing mice were randomized cage-wise using Research Randomizer (www.randomizer.org). Tumor size was measured using a caliper and their volumes were calculated using the equation of length × width^2^/2. When tumor size reached ~100–130 mm^3^, intratumor injection of either 50 μl PEPD siRNA (200 nM) or 50 μl scramble siRNA (200 nM) was initiated and was repeated every 3 days. The siRNAs were prepared in Lipofectamine RNAiMAX/Opti-MEM Reduced Serum Medium (1:9 by volume). Opti-MEM Reduced Serum Medium was purchased from Gibco (Cat# 31985-070). Treatments were stopped when average tumor size in the control mice reached ~550 mm^3^. Mice were killed at 24 h after the final treatment of MDA-MB-231 (p53^KO^) tumors and at 48 h after the final treatment of MDA-MB-231 (p53^R280K^) tumors, MDA-MB-231 (p53^R175H^) tumors, and HCC70 (p53^R248Q^) tumors. Treatment of HCC70 (p53^R248Q^) tumors was suspended for 7 days before the final dose, because the tumors in the PEPD siRNA group became too small to treat. All tumors were immediately removed from the mice, weighed, and snap frozen for subsequent molecular analysis.

### Statistics and reproducibility

All statistical analyses were carried out using GraphPad Prism 8. For two-group comparison, we used paired two-tailed *t*-test. For multigroup comparisons, we used analysis of variance followed by Tukey test. *P* value of 0.05 or lower was considered statistically significant. Sample size, mean, SD, SEM and *P* value are provided in each figure legend. Each replicate represents an independent sample, not repeated measurement of the same sample.

### Reporting summary

Further information on research design is available in the Nature Research Reporting Summary linked to this article.

## Supplementary information

Supplementary Information

Description of Additional Supplementary Files

Supplementary Data

Reporting Summary

## Data Availability

The authors declare that all the data supporting the findings of this study are available within the article and its Supplementary Information file and from the corresponding author upon reasonable request. Uncropped blots and gels are provided as supplementary figures in the Supplementary Information file (Supplementary Figs. 10–49). Source data for the graphs and charts in the figures are available in the Supplementary Data file.
