# Supplementary material for: Loss of peptidase D binding restores the tumor suppressor functions of oncogenic p53 mutants
Source: Commun Biol. 2021 Dec 8;4:1373. doi: 10.1038/s42003-021-02880-x (PMC8655031; doi:10.1038/s42003-021-02880-x)
Supplement: Supplementary file 1 — Supplementary Information [file 42003_2021_2880_MOESM1_ESM.pdf]

Yang et al., Supplementary Information

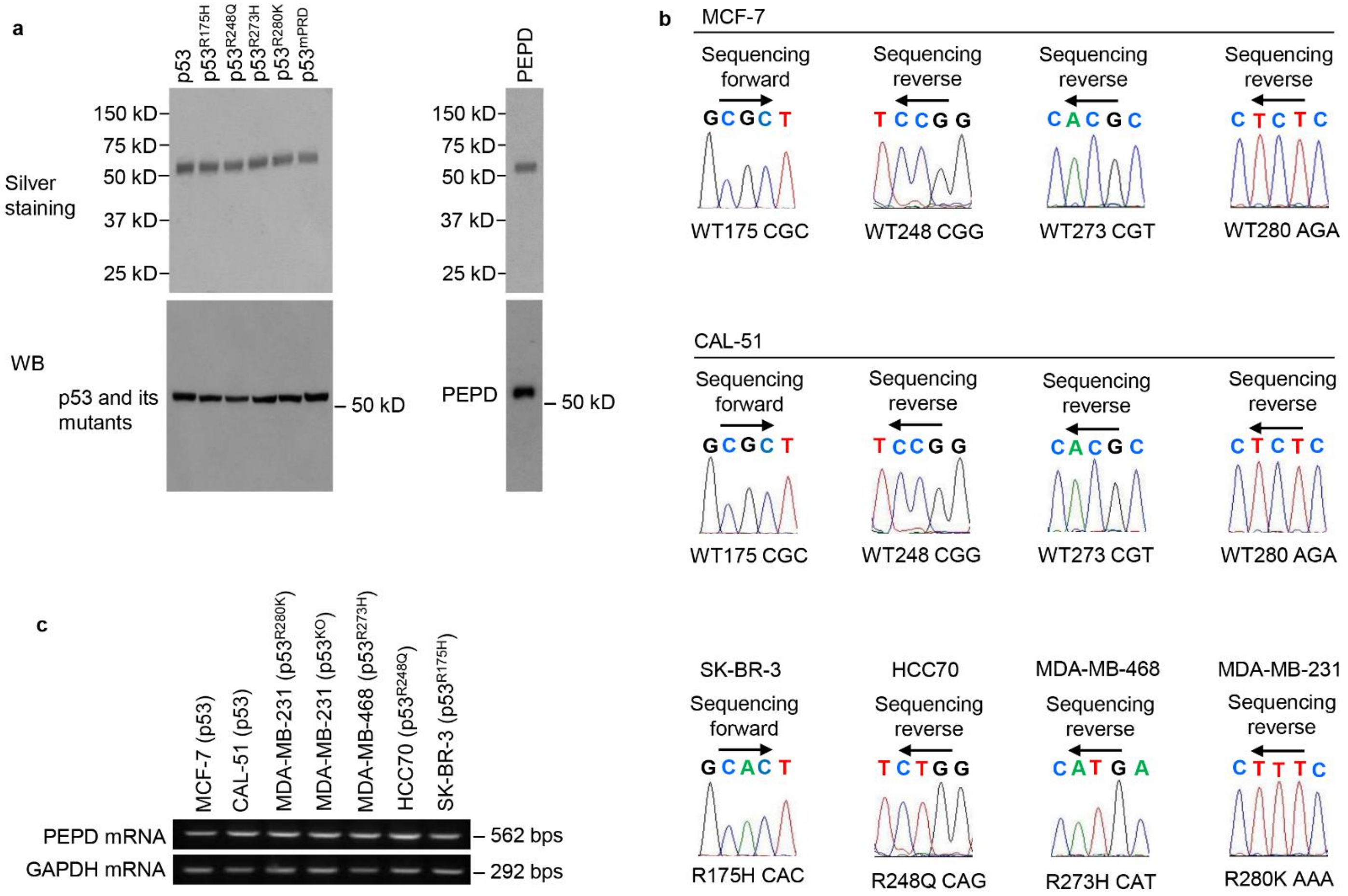

Supplementary Figure 1. Determining the purity of recombinant proteins and characterizing the cell lines used in the study. (**a**) Analysis of the purity of affinity-purified recombinant proteins, including p53, p53 mutants, and PEPD, by SDS-PAGE, followed by silver staining and WB analysis. (**b**) Sanger sequencing of p53 and p53 mutants in cell lines, showing the sequences coding amino acids #175, #248, #273, and #280. (**c**) Measurement of PEPD mRNA level in cells by RT-PCR, using GAPDH mRNA as a control.

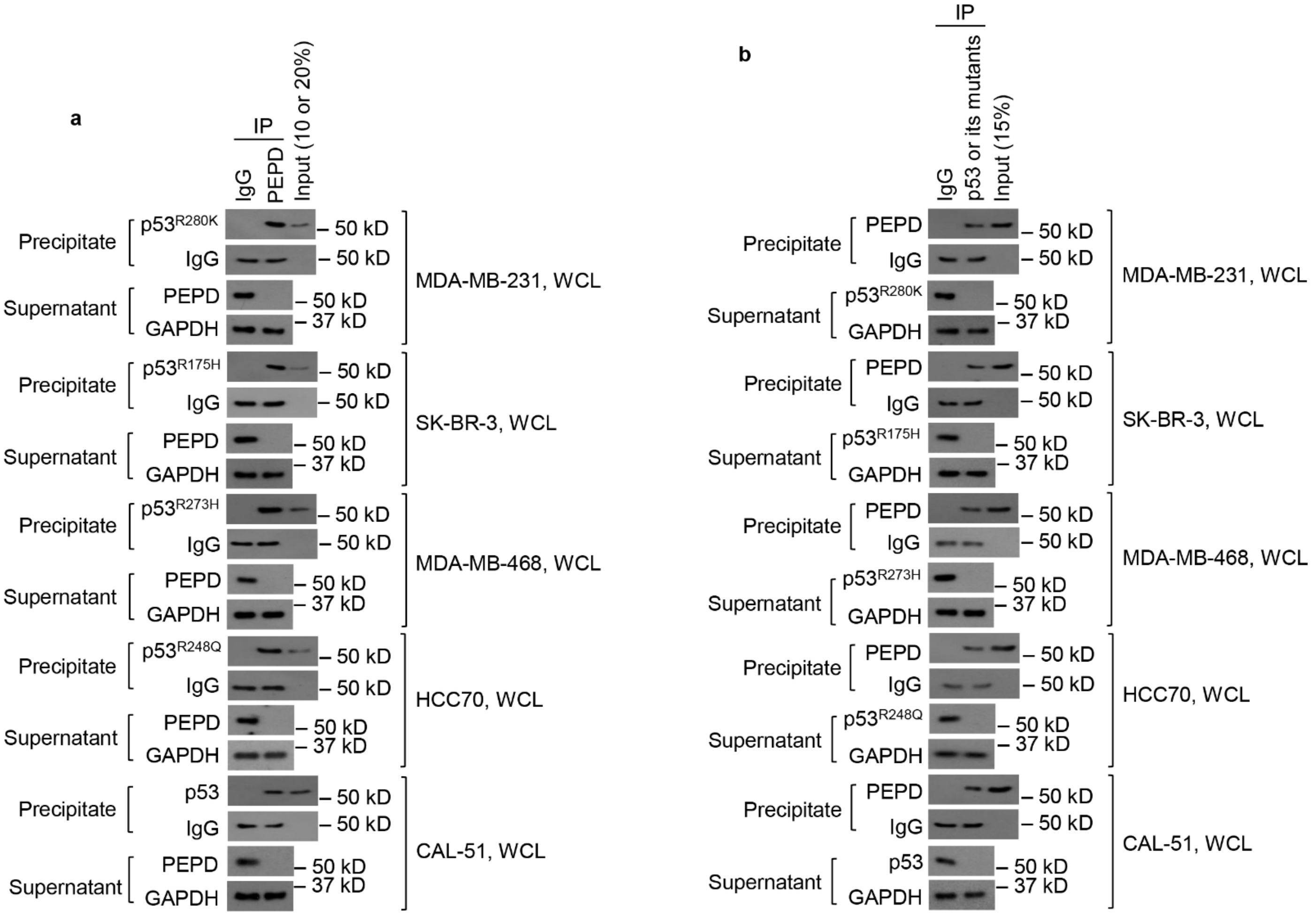

Supplementary Figure 2. PEPD binds to p53 mutants in cells. WCL was prepared from untreated cells carrying a p53 mutant. CAL-51 cells carrying p53 were included for comparison. (**a**) PEPD in WCL was completely pulled down by IP using a PEPD antibody in excess. An isotype-matched IgG was used as a control. The precipitate and an input were analyzed by WB for p53 and p53 mutants. The input was 20% (CAL-51 WCL) or 10% (other samples) of each sample used in the PEPD IP. The supernatants were also analyzed by WB to confirm that PEPD was completely pulled down. (**b**) p53 and p53 mutants in WCL were completely pulled down by IP using an antibody in excess. An isotype-matched IgG was used as a control. The precipitate and an input were analyzed by WB for PEPD. The input was 15% of each sample used in the IP of p53 and p53 mutants. The supernatant samples were also analyzed by WB to confirm that p53 and p53 mutants were completely pulled down. Percentages of p53 and p53 mutants binding to PEPD and vice versa were determined by comparing their band intensities with that of the inputs using ImageJ. Three independent experiments, one of which is shown here, were carried out to calculate the binding percentages shown in Fig. 2b-c.

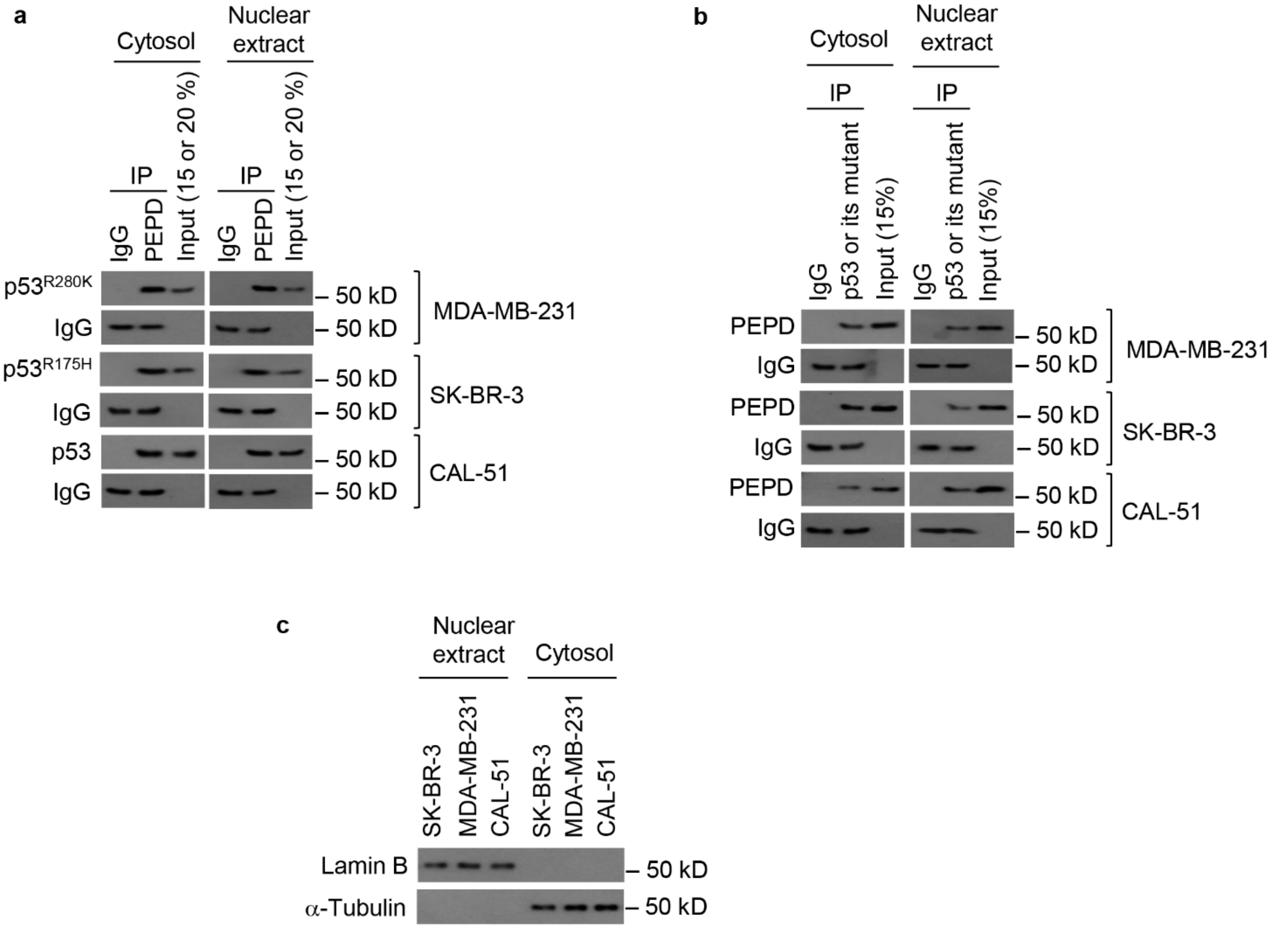

Supplementary Figure 3. PEPD binds to p53 mutants in cytosol and nuclear extract. All samples were prepared from untreated cells. CAL-51 cells carrying p53 were included for comparison. (**a**) PEPD in cytosol and nuclear extract was completely pulled down by IP using a PEPD antibody in excess. An isotype-matched IgG was used as a control. The precipitate and an input were analyzed by WB for p53 and p53 mutants. The input was 20% (CAL-51 samples) or 15% (other samples) of each sample used in the PEPD IP. (**b**) p53 and p53 mutants in cytosol and nuclear extract were completely pulled down by IP using an antibody in excess. An isotype-matched IgG was used as a control. The precipitate and an input were analyzed by WB for PEPD. The input was 15% of each sample used in the IP of p53 and p53 mutants. Percentages of p53 and p53 mutants binding to PEPD and vice versa were determined by comparing their band intensities with that of the inputs using ImageJ. Three independent experiments, one of which is shown in **a, b**, were carried out to calculate the binding percentages shown in Fig. 2b-c. (**c**) WB analysis of nuclear extract and cytosol for lamin B and α-tubulin to rule out cross-contamination.

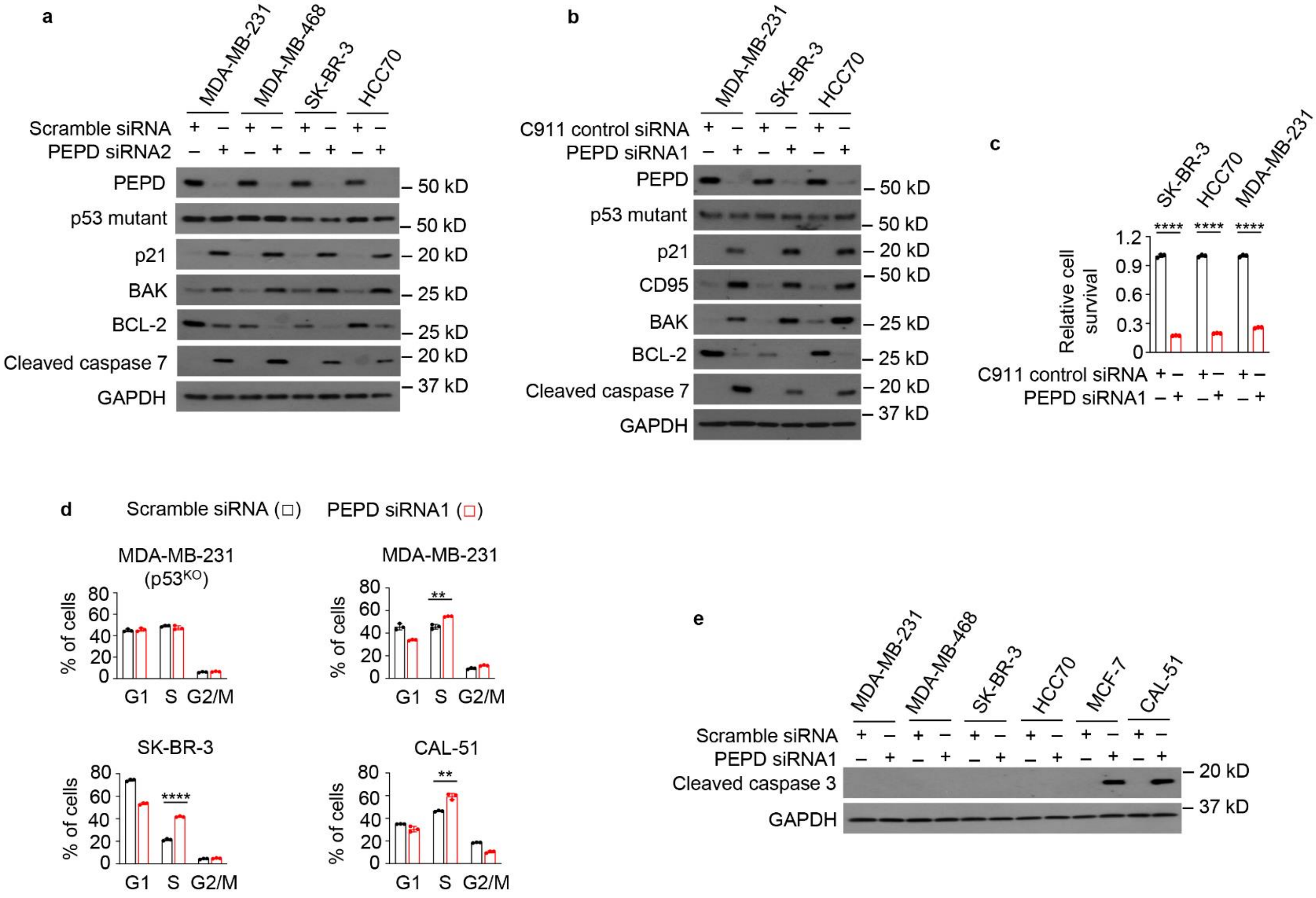

Supplementary Figure 4. PEPD KD reactivates p53 mutants. (**a**) Cells were treated by scramble or PEPD siRNA (10 nM) for 48 h. PEPD and other proteins in WCL were analyzed by WB, using GAPDH as a loading control. (**b, c**) Cells were treated by control siRNA (C911-PEPD) or PEPD siRNA (10 nM) for 72 h. PEPD and other proteins were analyzed by WB, using GAPDH as a loading control in **b**, and cell viability was measured by CellTiter-Glo assay in **c**. (**d**) Cells were treated by scramble or PEPD siRNA (10 nM) for 48 h. Cell cycle progression was measured by flow cytometry. (**e**) Cells were treated by scramble and PEPD siRNA (10 nM) for 48 h. Cleaved caspase 3 in WCL was analyzed by WB, using GAPDH as a loading control. MDA-MB-231 ($p53^{KO}$) cells and CAL-51 cells which carry p53 were included for comparison in **d**. CAL-51 cells as well as MCF-7 cells which also carry p53, were included for comparison in **e**. The bar-dot plots in **c, d** show individual values and mean ± SD (n = 3). **P<0.01, ****P<0.0001, by paired two-tailed t-test.

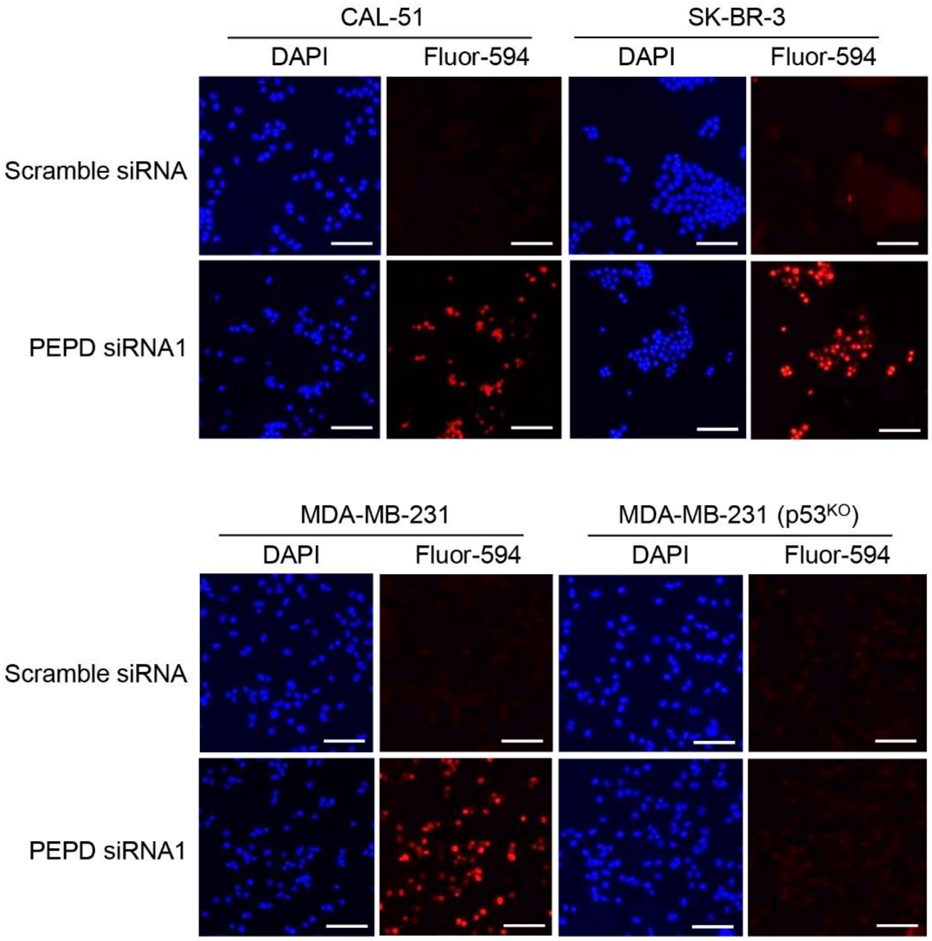

Supplementary Figure 5. p53 mutants reactivated by PEPD KD induce apoptosis. Cells were treated by scramble and PEPD siRNA (10 nM) for 72 h and then subjected to TUNEL fluorescence staining with Alexa Fluor-594 and nuclear fluorescence staining with DAPI. Scale bar: 100 μm. CAL-51 cells carrying p53 and MDA-MB-231 ($p53^{KO}$) cells were included for comparison.

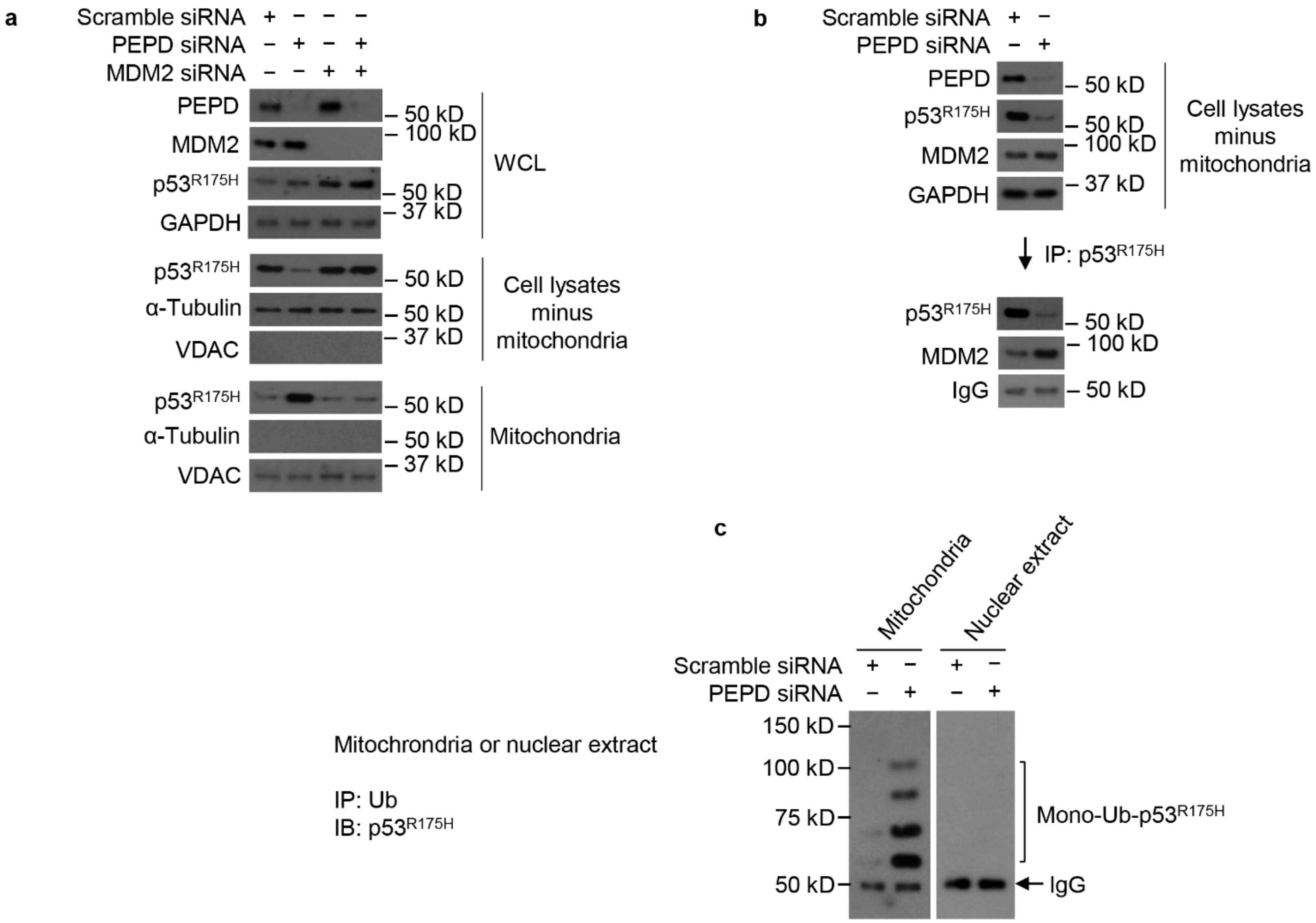

Supplementary Figure 6. MDM2 is critical for mitochondrial enrichment of p53^{R175H}. (a) SK-BR-3 cells (p53^{R175H}) were treated with scramble siRNA or MDM2 siRNA (10 nM) and 24 h later treated with scramble siRNA or PEPD siRNA (10 nM) for 48 h. PEPD and other proteins in WCL and subcellular fractions were analyzed by WB. GAPDH, VDAC and α-tubulin were measured as loading controls and to rule out cross-contamination. (**b**) SK-BR-3 cells were treated with siRNA (10 nM) for 48 h. PEPD and other proteins in a sample (cell lysate minus mitochondria) were analyzed by WB. GAPDH is a loading control. The same samples were also subjected to p53^{R175H} IP, and the precipitate was analyzed by WB for p53^{R175H} and MDM2. (**c**) SK-BR-3 cells were treated with siRNA (10 nM) for 48 h, with ubiquitin aldehyde (100 μM) added in the final 4 h to inhibit de-ubiquitination, from which mitochondria and nuclear extract were isolated. Mitochondria and nuclear extract were subjected to IP by a ubiquitin (Ub) antibody, and the precipitate was analyzed by WB for p53^{R175H}.

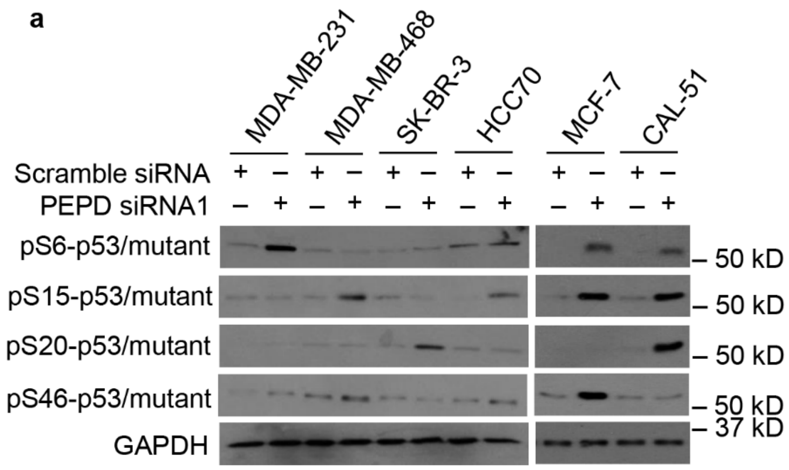

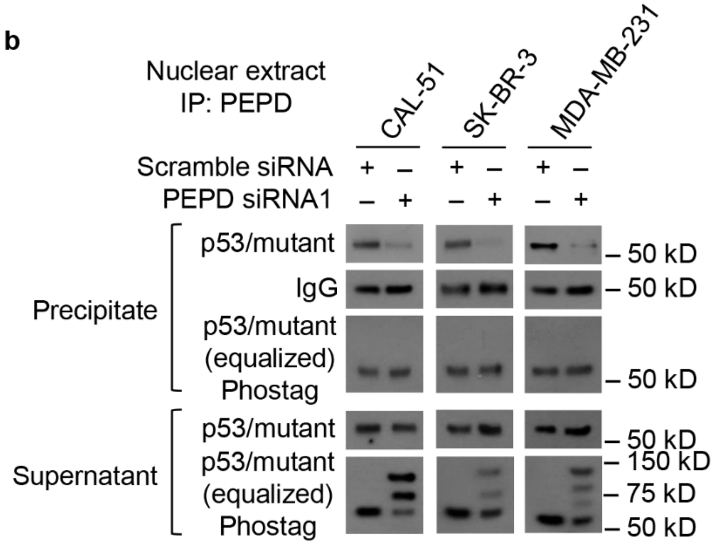

Supplementary Figure 7. PEPD KD stimulates phosphorylation of p53 mutants. (**a**) Cells were treated with siRNA (10 nM) for 48 h. Phospho-p53 mutants in WCL was analyzed by WB. GAPDH is a loading control. MCF-7 cells and CAL-51 cells, both of which carry p53, were included for comparison. (**b**) Cells were treated by siRNA (10 nM) for 48 h, from which nuclear extracts were prepared. CAL-51 cells were included for comparison. The nuclear extracts were subjected to PEPD IP. Both the precipitate and the supernatant were analyzed by WB and phostag WB for p53 and p53 mutants. Different amounts of samples were used in phostag WB, in order to equalize loading of p53 and p53 mutants.

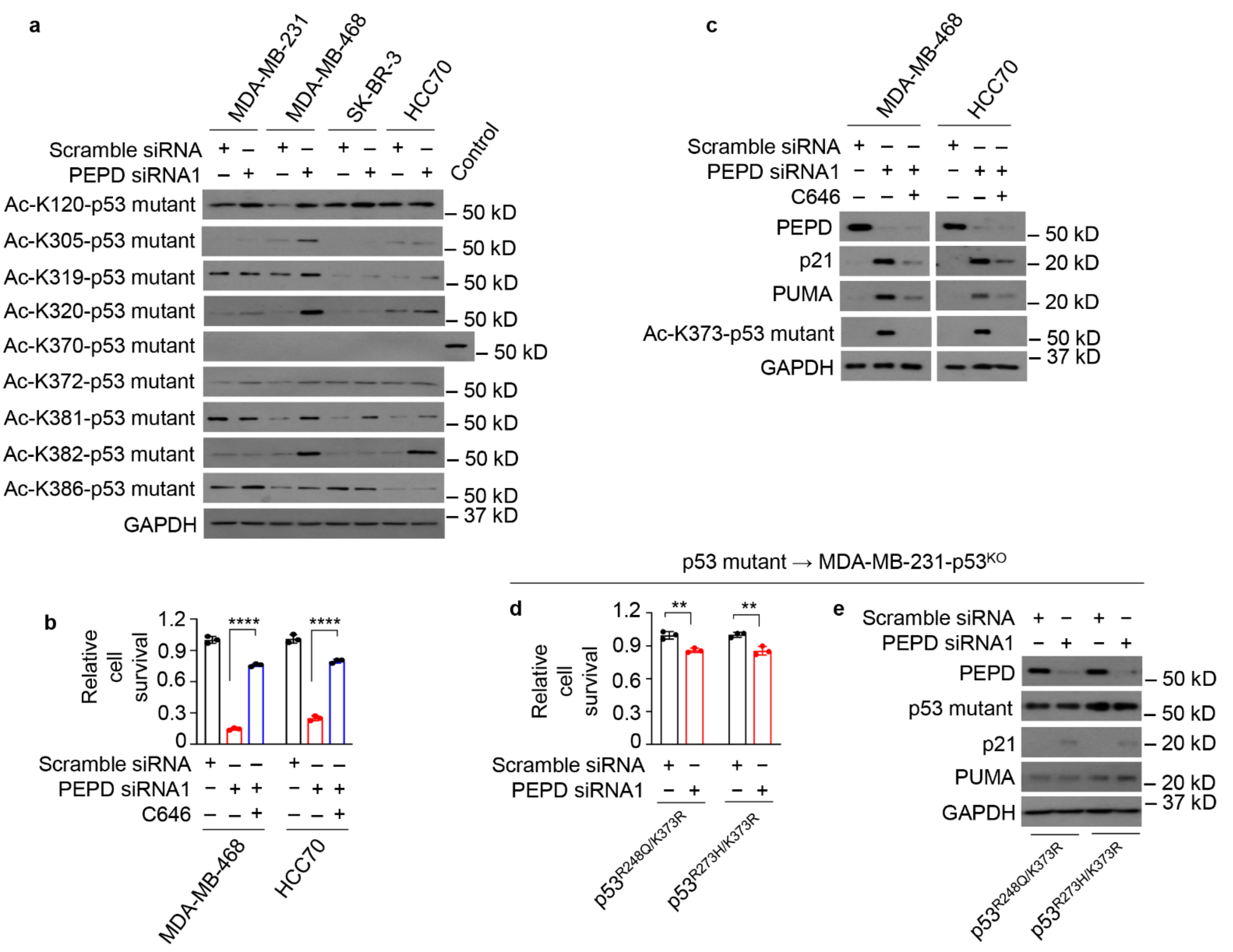

Supplementary Figure 8. Acetylation of p53 mutants is critical for their reactivation by PEPD KD. (**a**) Cells were treated by scramble and PEPD siRNA (10 nM) for 48 h. Acetylated p53 mutants in WCL were analyzed by WB. WCL of untreated HepG2 cells was used as a positive control for Ac-K370-p53. (**b, c**) Cells were treated by scramble and PEPD siRNA (10 nM) with or without C646 (8 μM) for 72 h. Cell viability was measured by trypan blue assay in **b**. PEPD and other proteins in WCL were analyzed by WB in **c**. (**d, e**) MDA-MB-231 ($p53^{KO}$) cells were transfected with a plasmid expressing a p53 mutant, including $p53^{R248Q/K373R}$ and $p53^{R273H/K373R}$, and 24 h later treated with scramble and PEPD siRNA (10 nM) for 96 h. The relatively long siRNA treatment time was for harvesting enough cells for analysis. Cell viability was measured by trypan blue assay in **d**. PEPD and other proteins in WCL were analyzed by WB in **e**. GAPDH is a loading control in **a, c, e**. The bar-dot plots in **b, d** show individual value and mean ± SD (n=3). **P<001, ****P<0.0001, by paired two-tailed t-test.

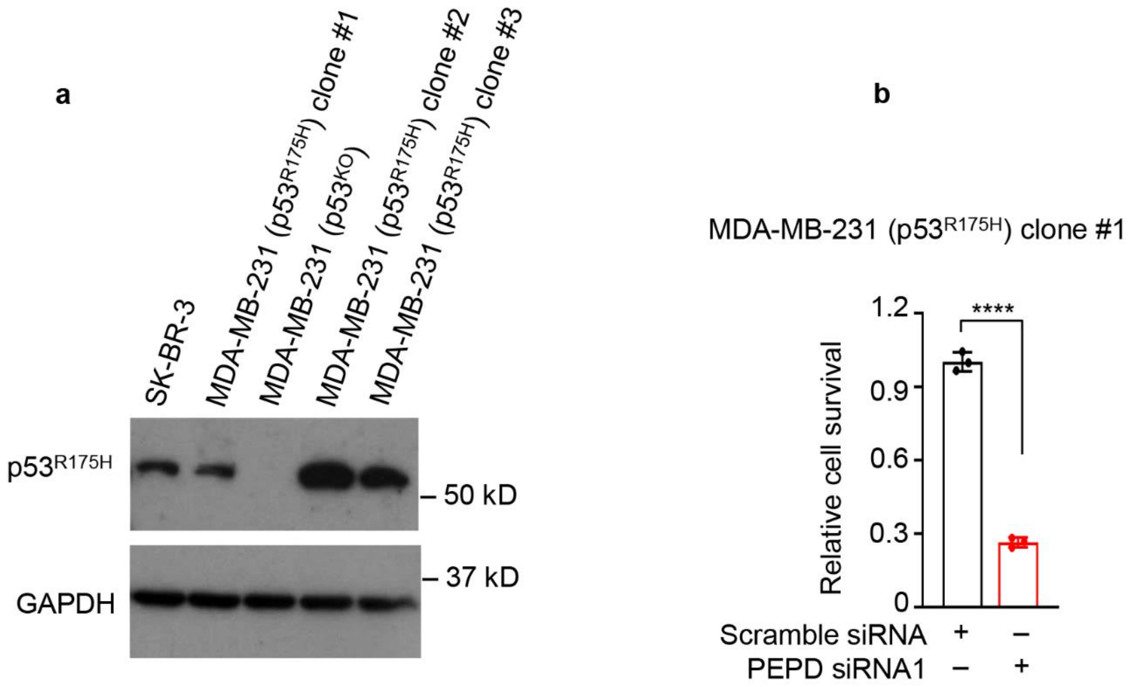

Supplementary Figure 9. Generating and characterizing MDA-MB-231 cells stably expressing p53^{R175H}. (**a**) MDA-MB-231 (p53^{KO}) cells were transfected with p53^{R175H}. Stable clones expressing the p53 mutant were selected by puromycin treatment. WCL was analyzed by WB for p53^{R175H} expression. WCL of SK-BR-3 cells was included for comparison in WB. GAPDH is a loading control. (**b**) Cells were treated by siRNA (10 nM) for 72 h. Cell viability was measured by trypan blue assay. The bar-dot plot shows individual values and mean ± SD (n=3), ****$P<0.0001$, by paired two-tailed t-test.

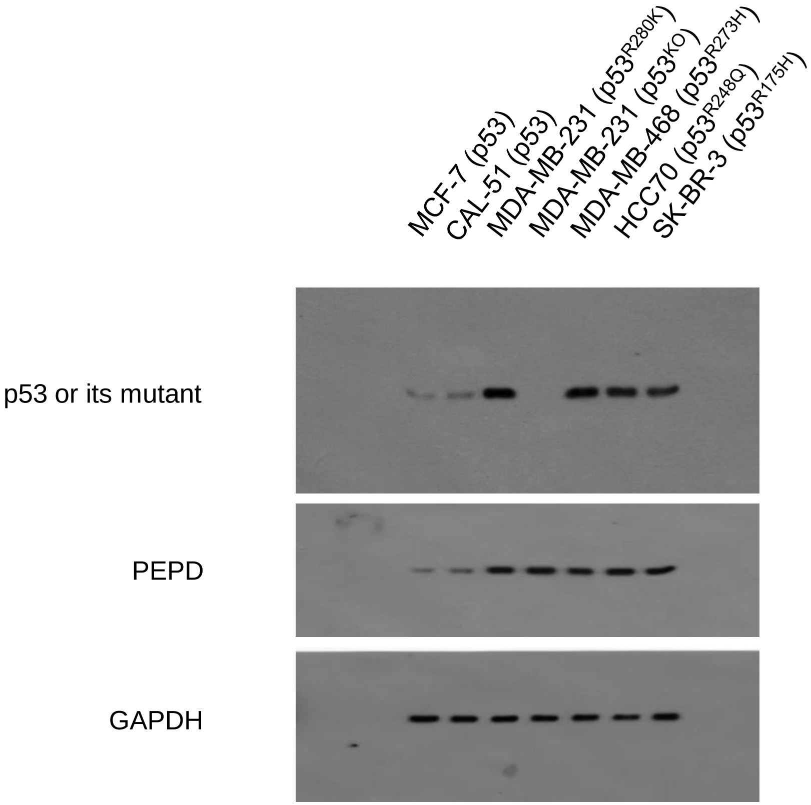

Supplementary Figure 10. Uncropped blots for Figure 2a

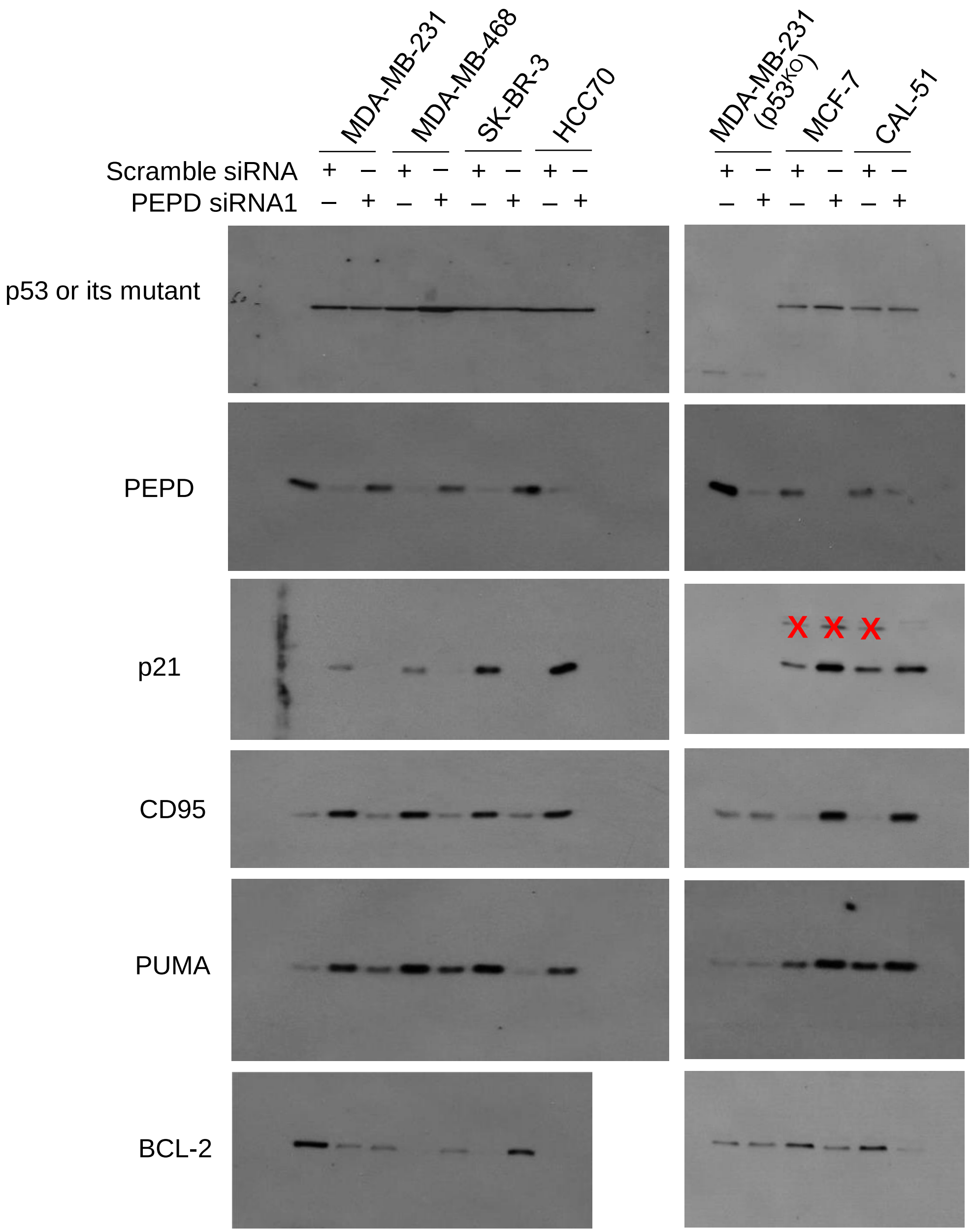

Supplementary Figure 11. Uncropped blots for Figure 3b

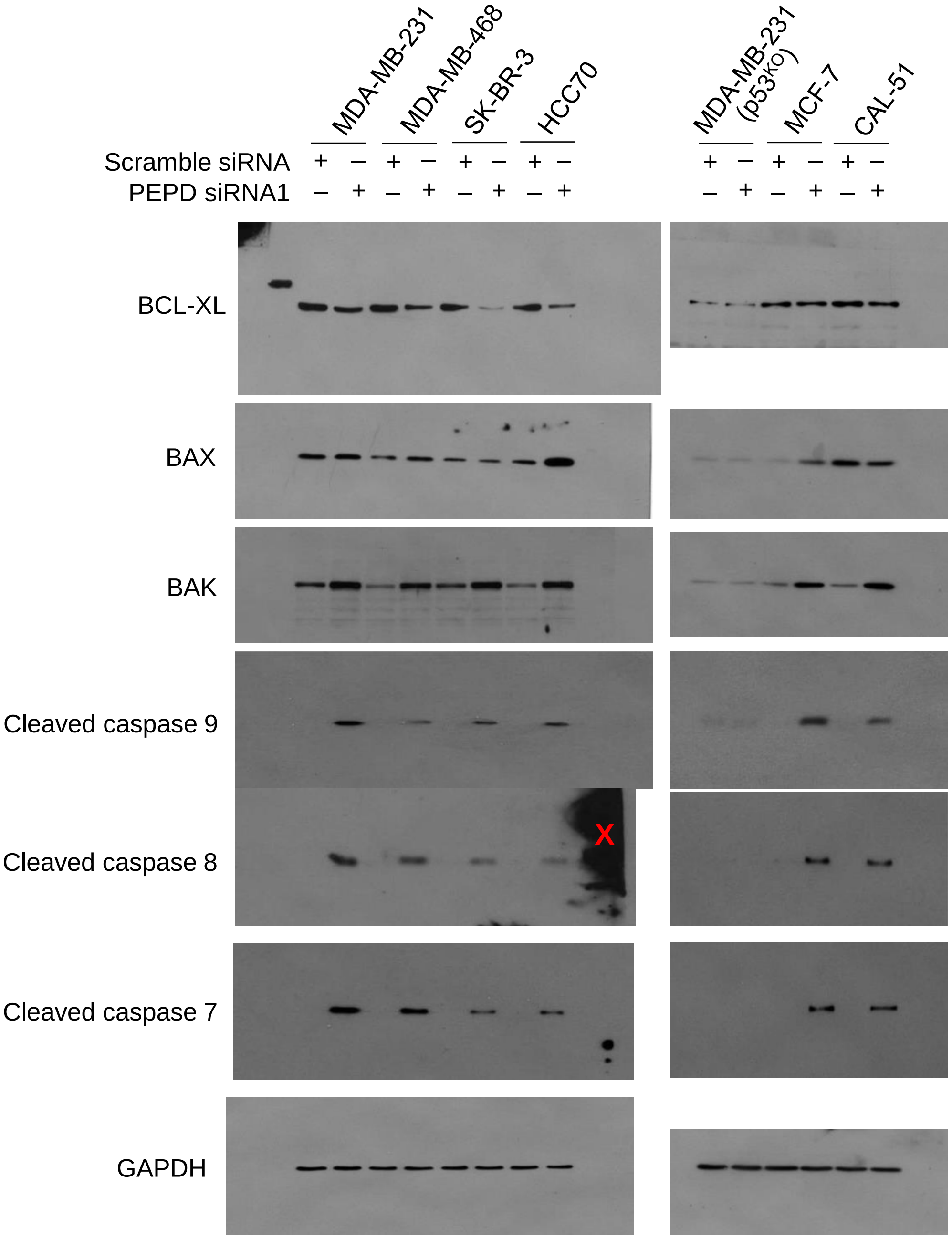

Supplementary Figure 12. Uncropped blots for Figure 3b

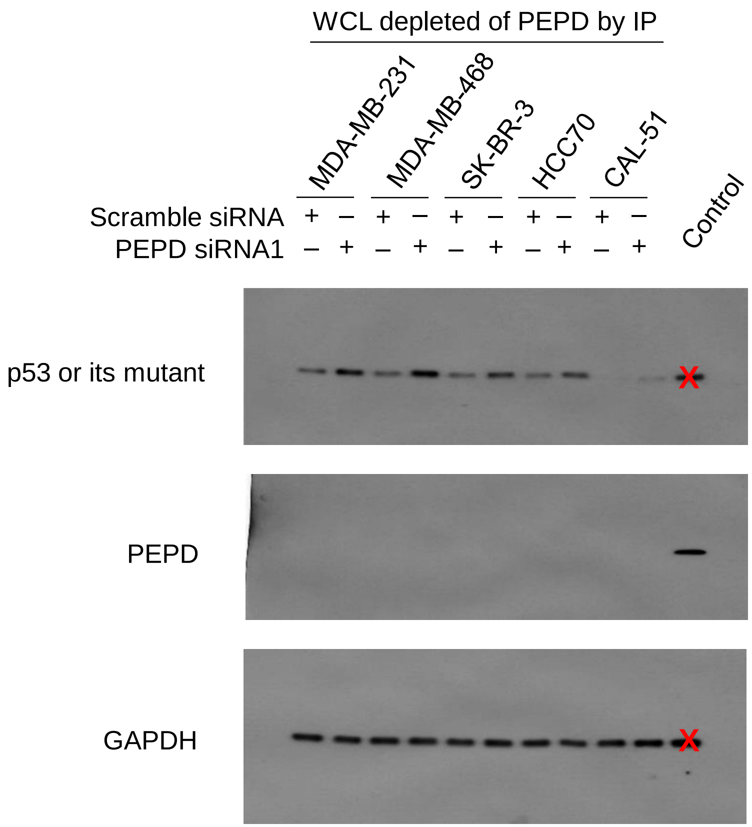

Supplementary Figure 13. Uncropped blots for Figure 3c

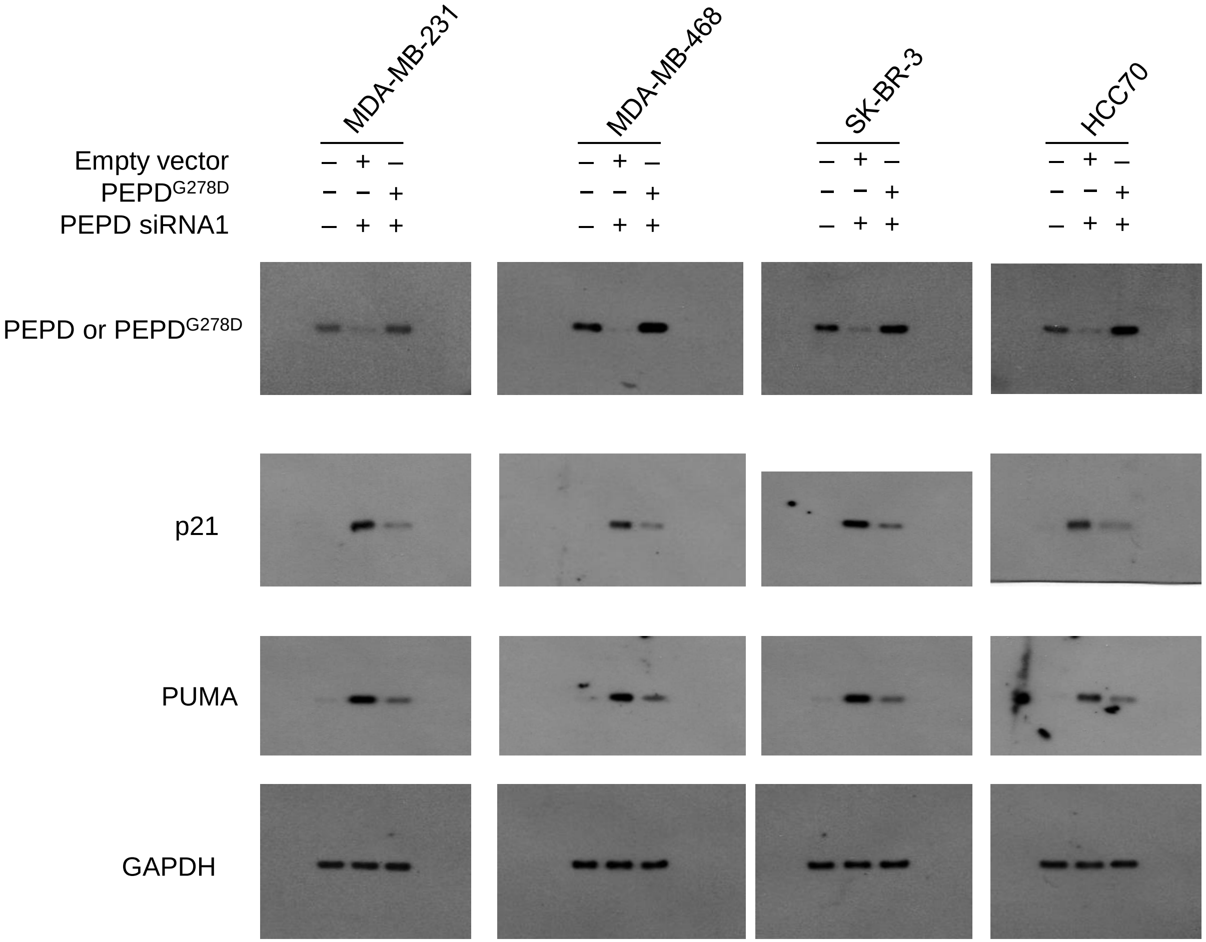

Supplementary Figure 14. Uncropped blots for Figure 3e

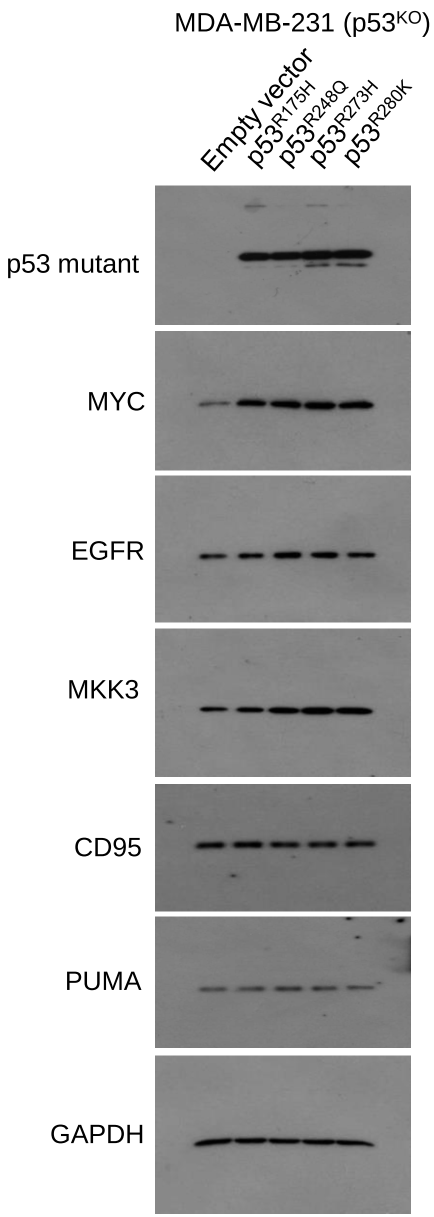

Supplementary Figure 15. Uncropped blots for Figure 4a

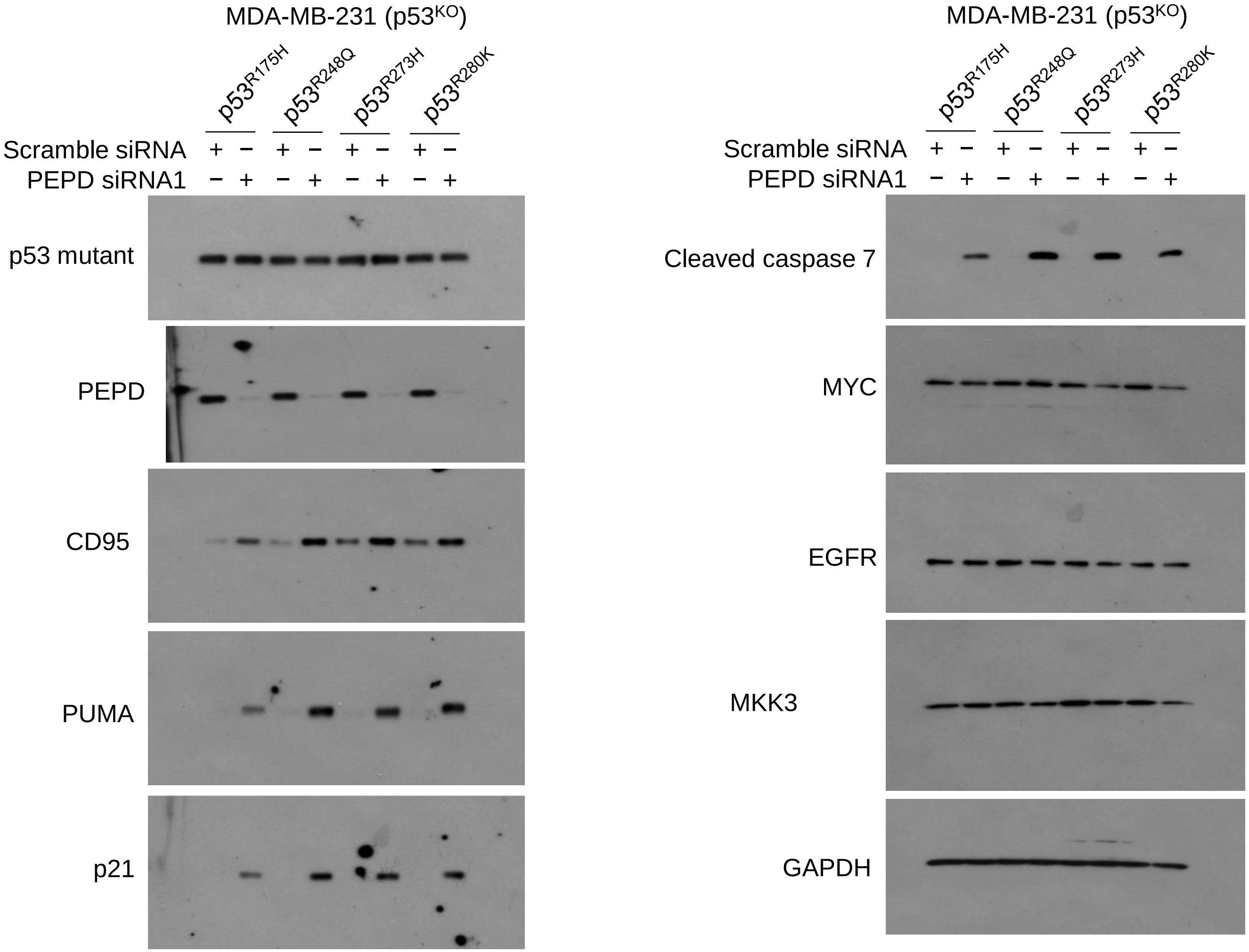

Supplementary Figure 16. Uncropped blots for Figure 4c

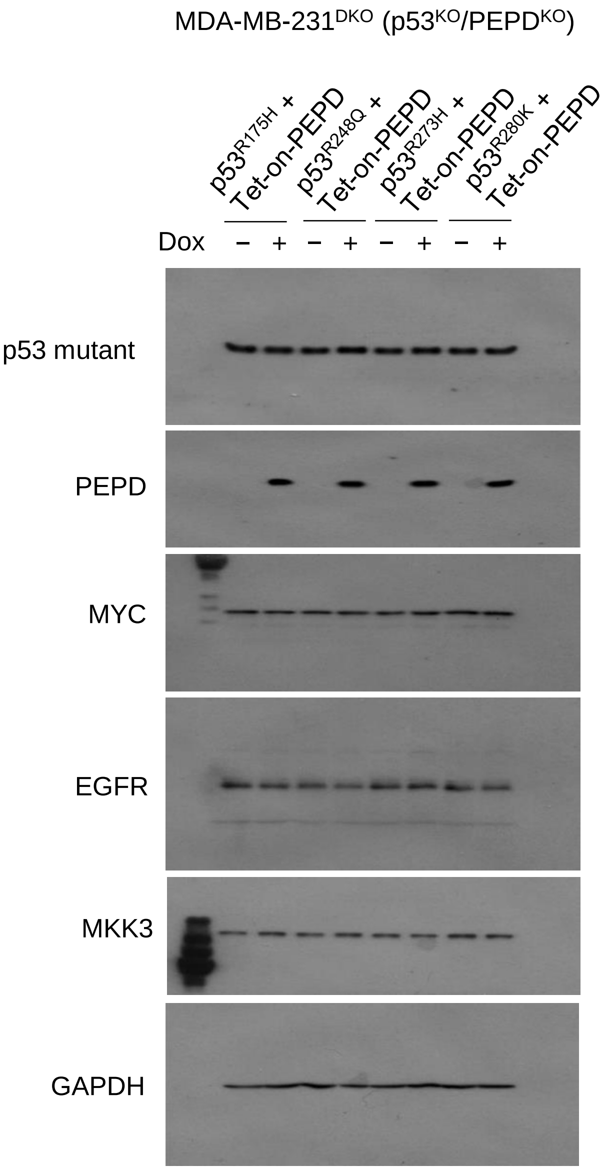

Supplementary Figure 17. Uncropped blots for Figure 4d

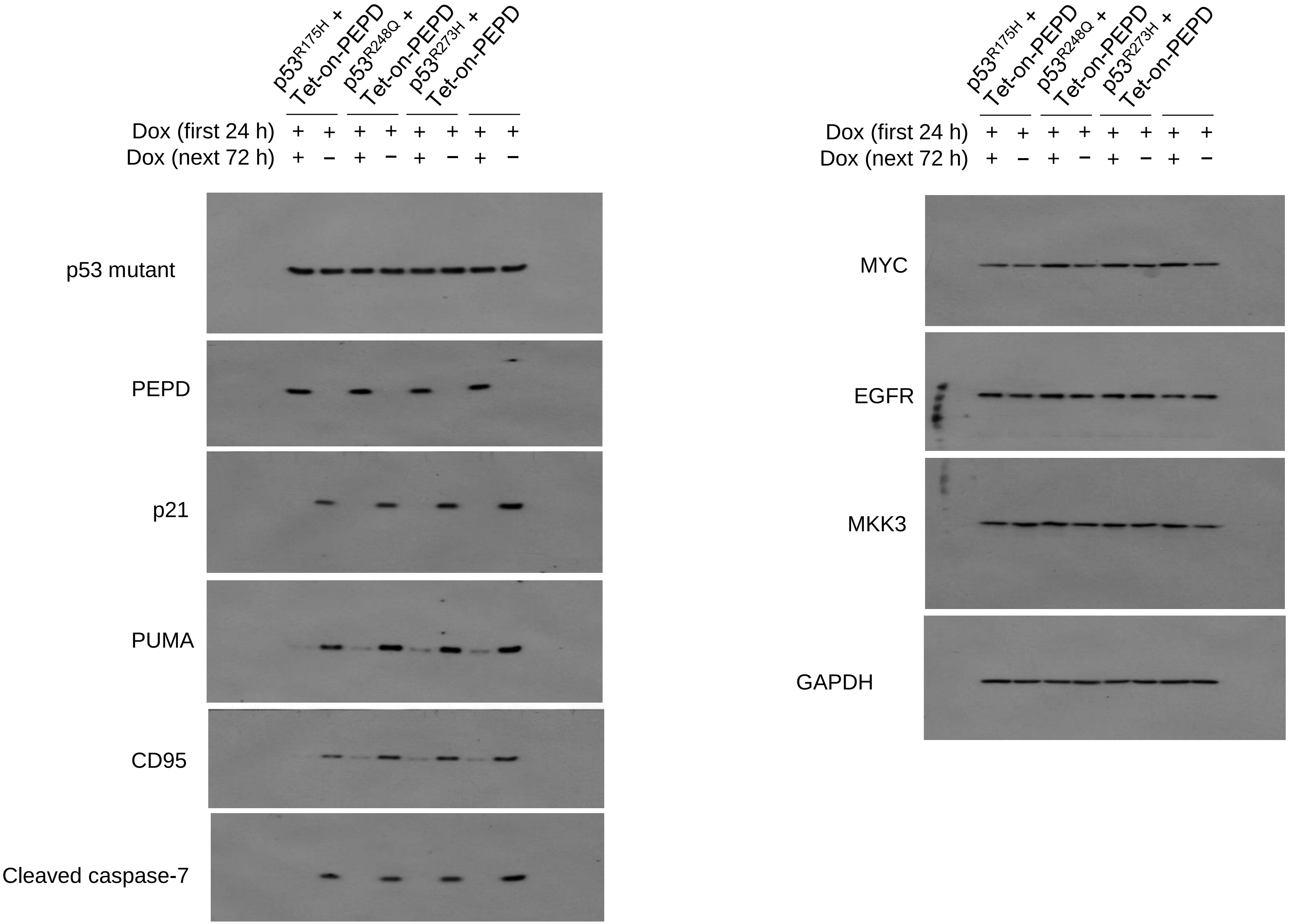

Supplementary Figure 18. Uncropped blots for Figure 4e

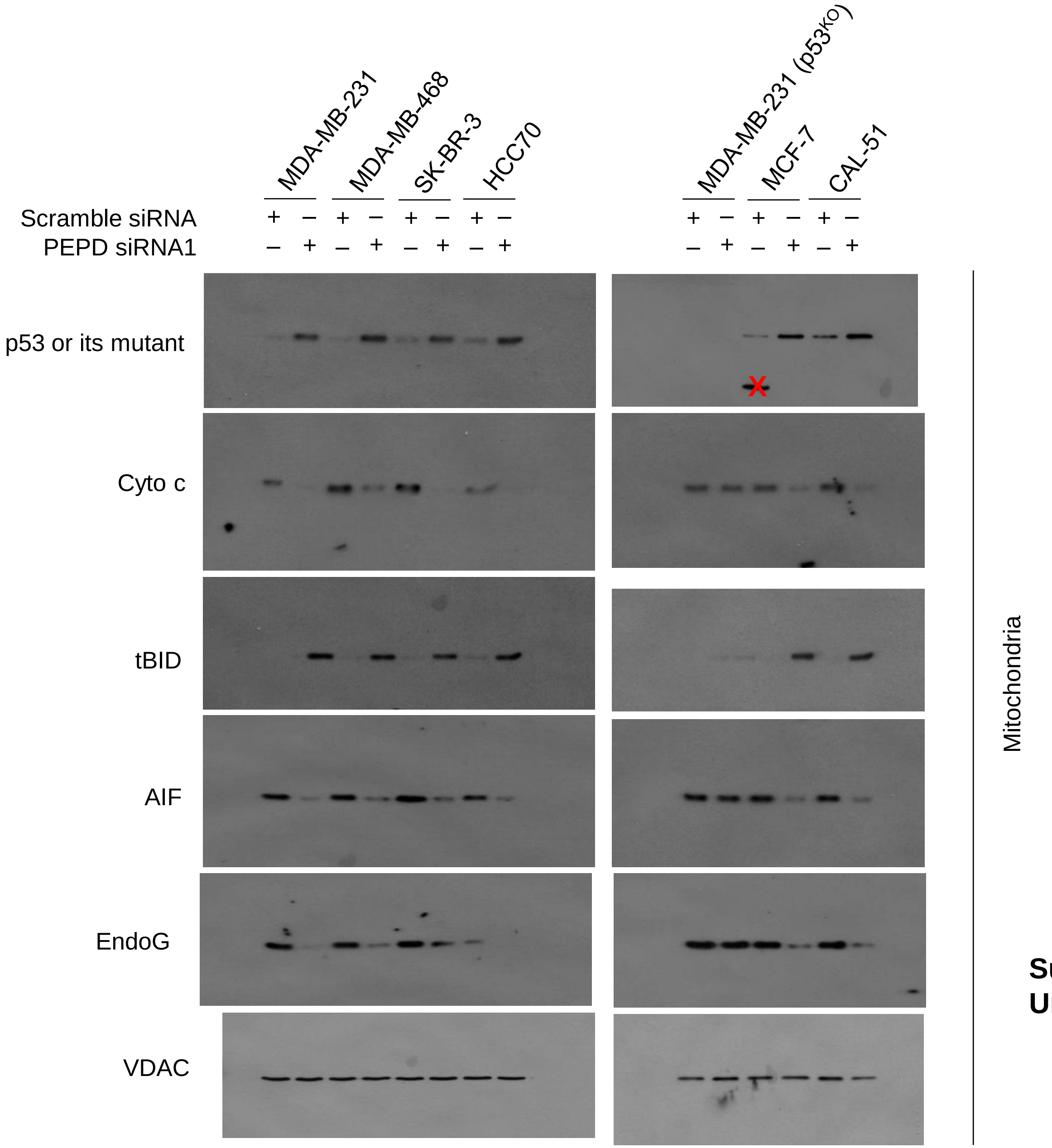

Supplementary Figure 19.
Uncropped blots for Figure 5a

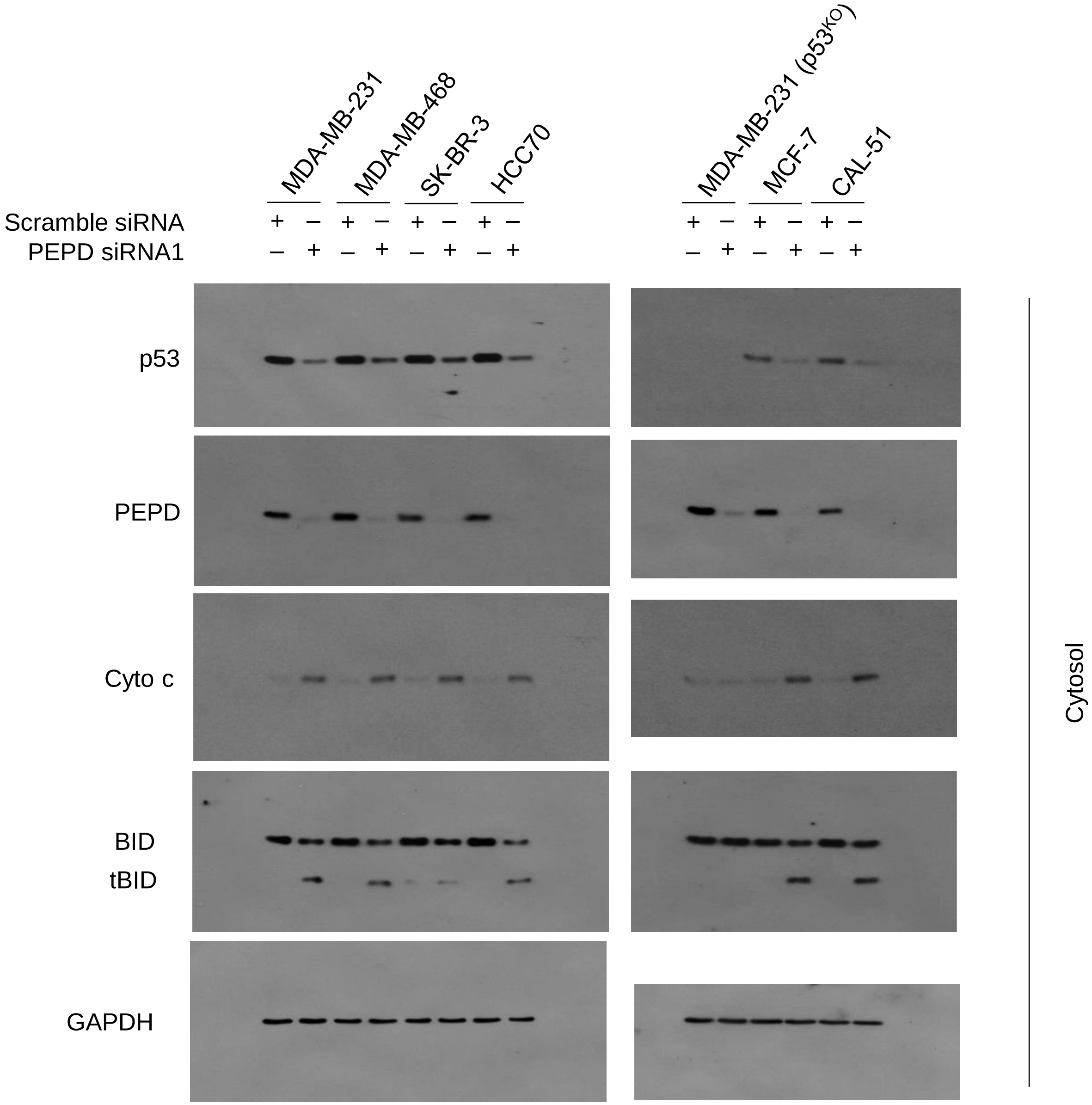

Supplementary Figure 20. Uncropped blots for Figure 5a

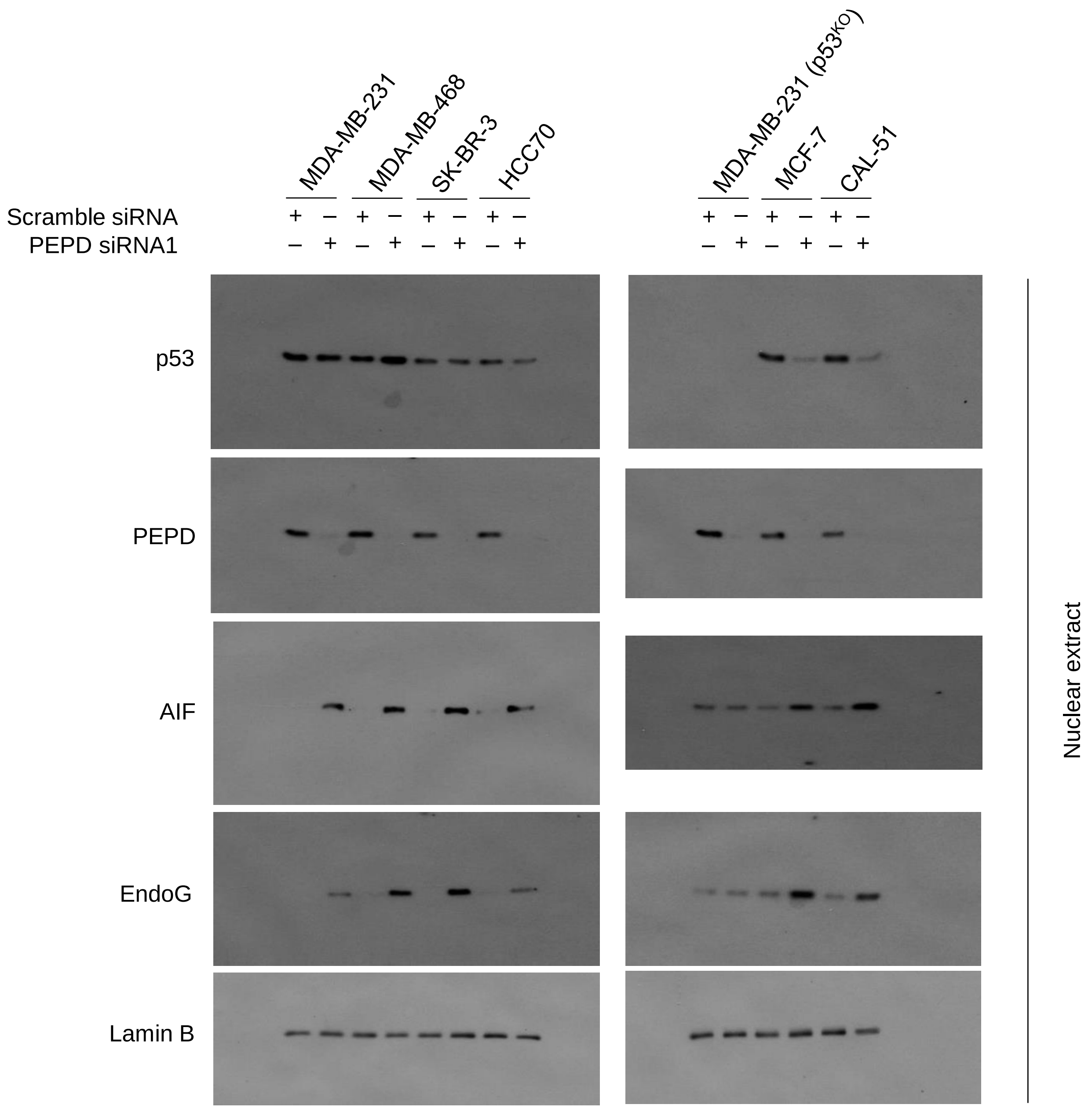

Supplementary Figure 21. Uncropped blots for Figure 5a

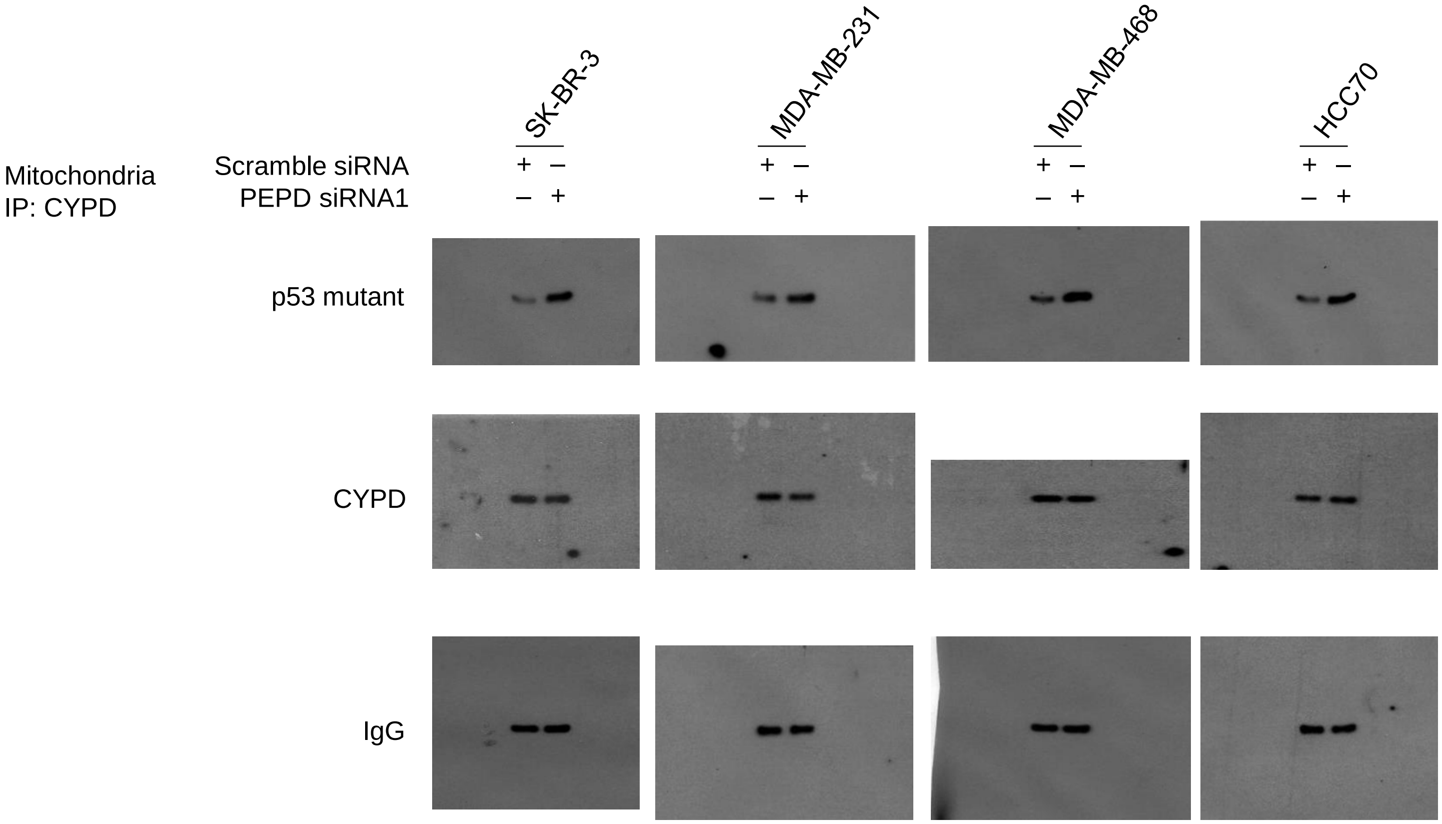

Supplementary Figure 22. Uncropped blots for Figure 5c

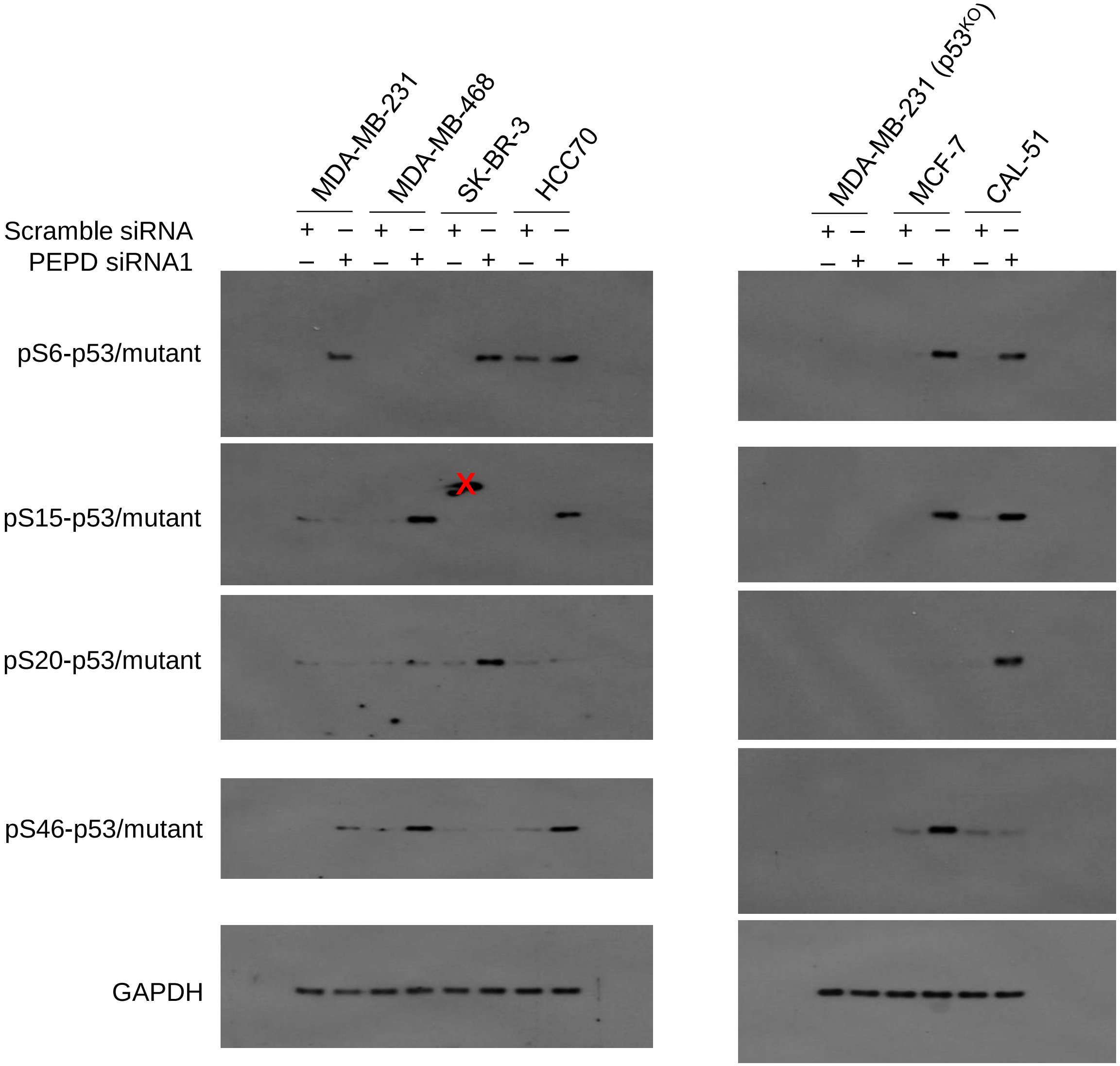

Supplementary Figure 23. Uncropped blots for Figure 6a

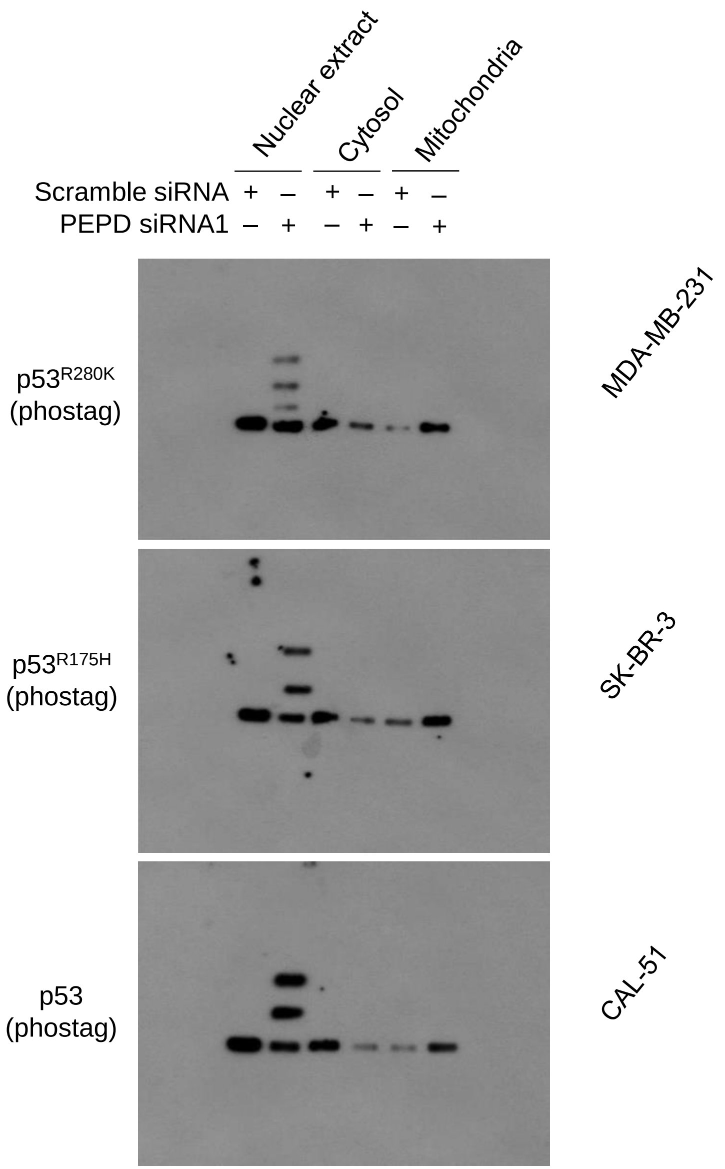

Supplementary Figure 24. Uncropped blots for Figure 6b

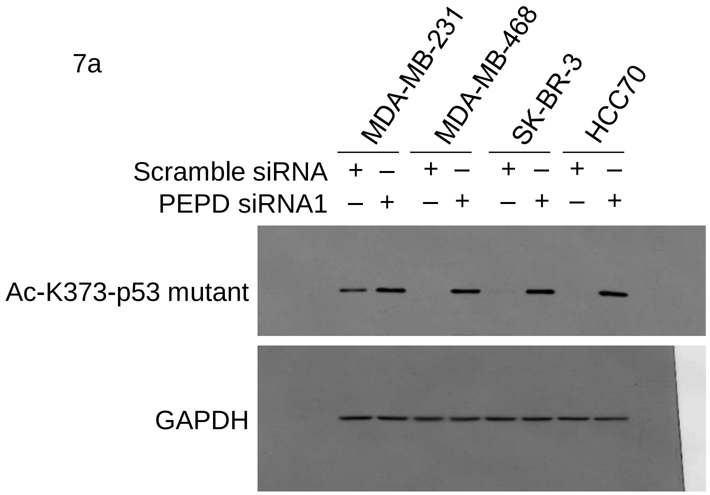

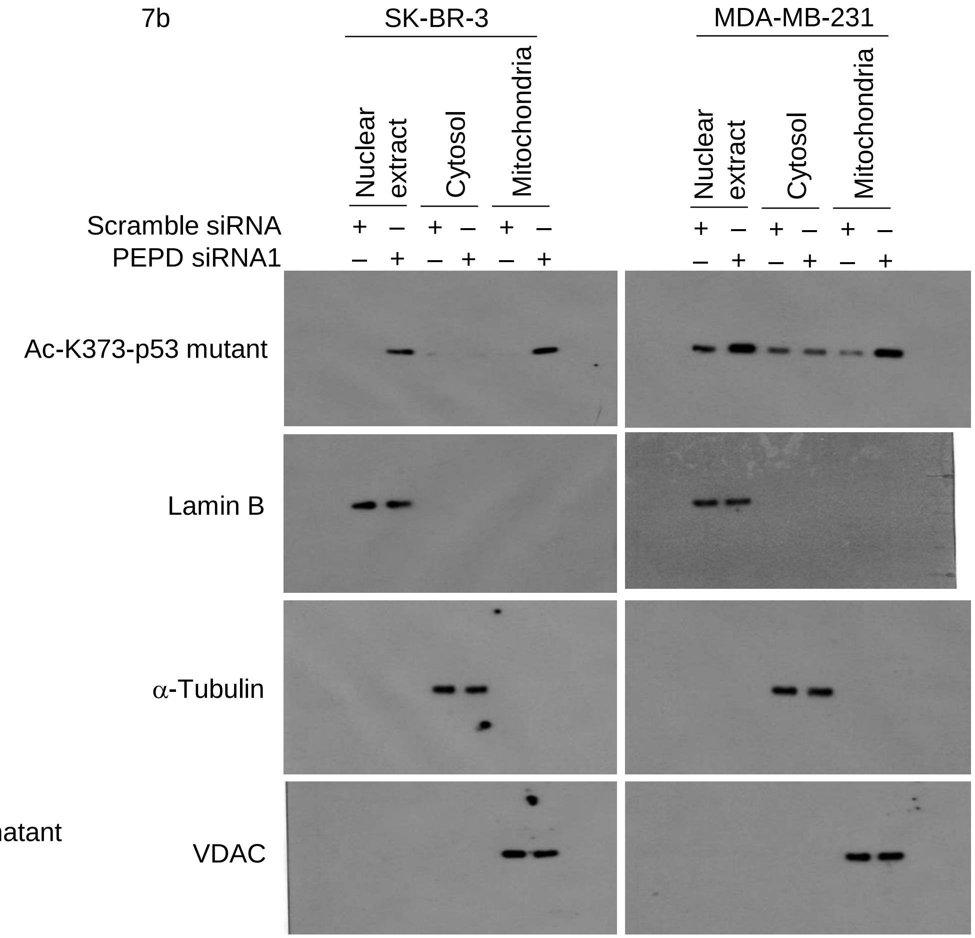

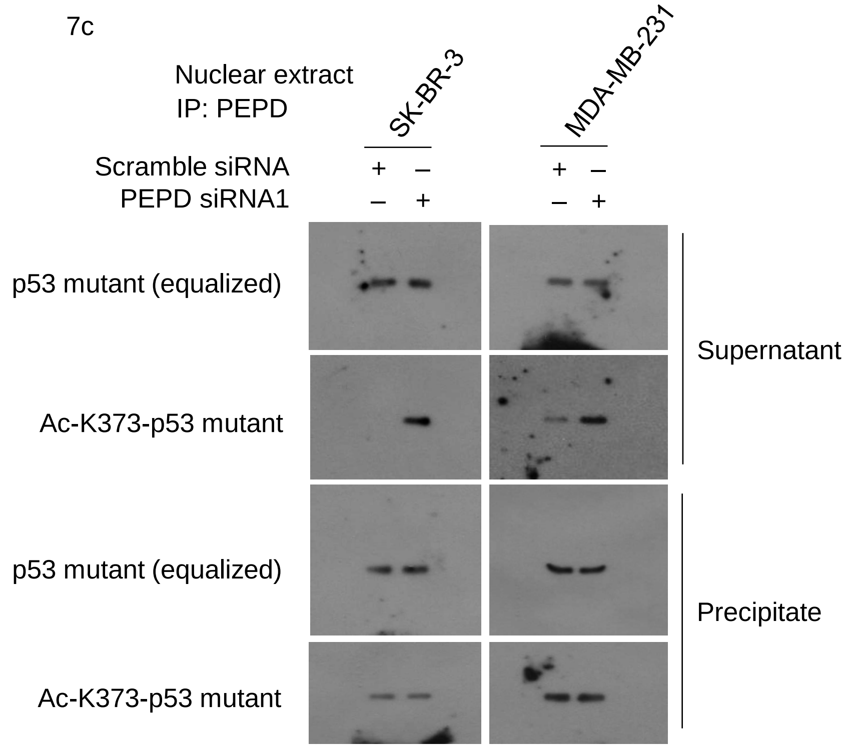

Supplementary Figure 25. Uncropped blots for Figure 7a, 7b, and 7c

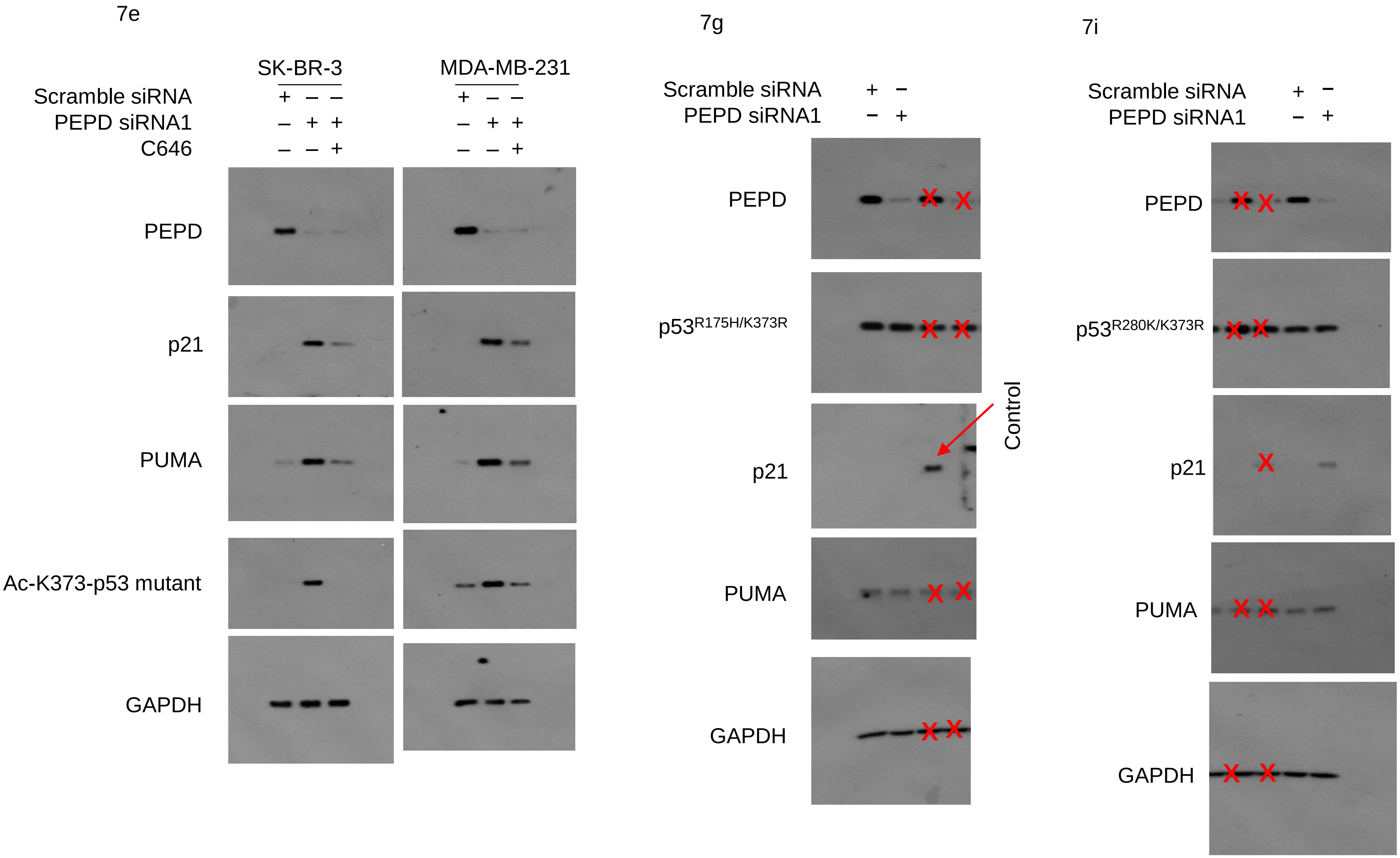

Supplementary Figure 26. Uncropped blots for Figure 7e, 7g, and 7i

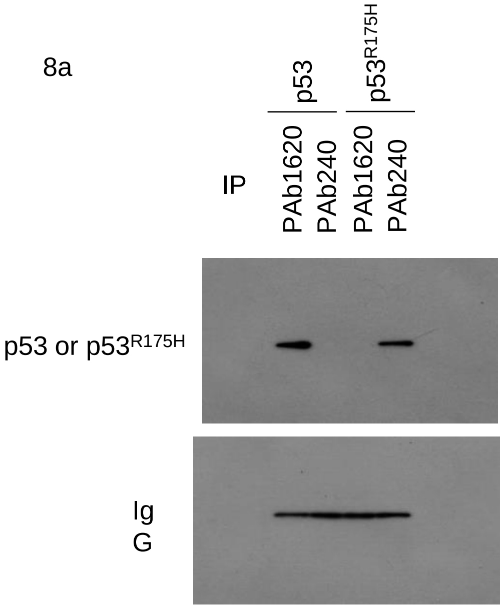

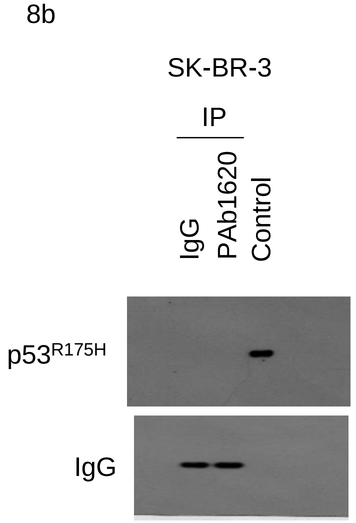

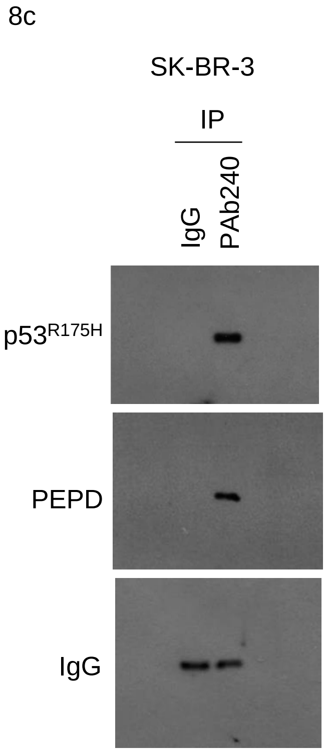

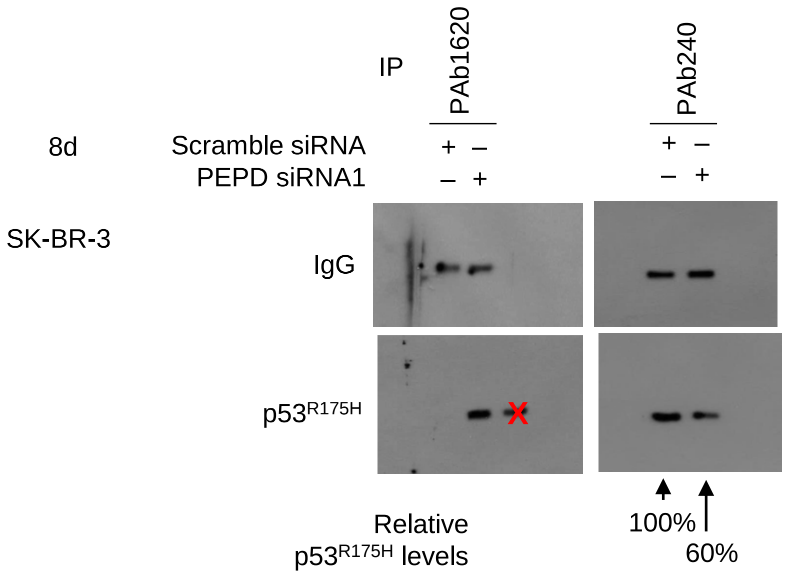

Supplementary Figure 27. Uncropped blots for Figure 8a, 8b, 8c, and 8d

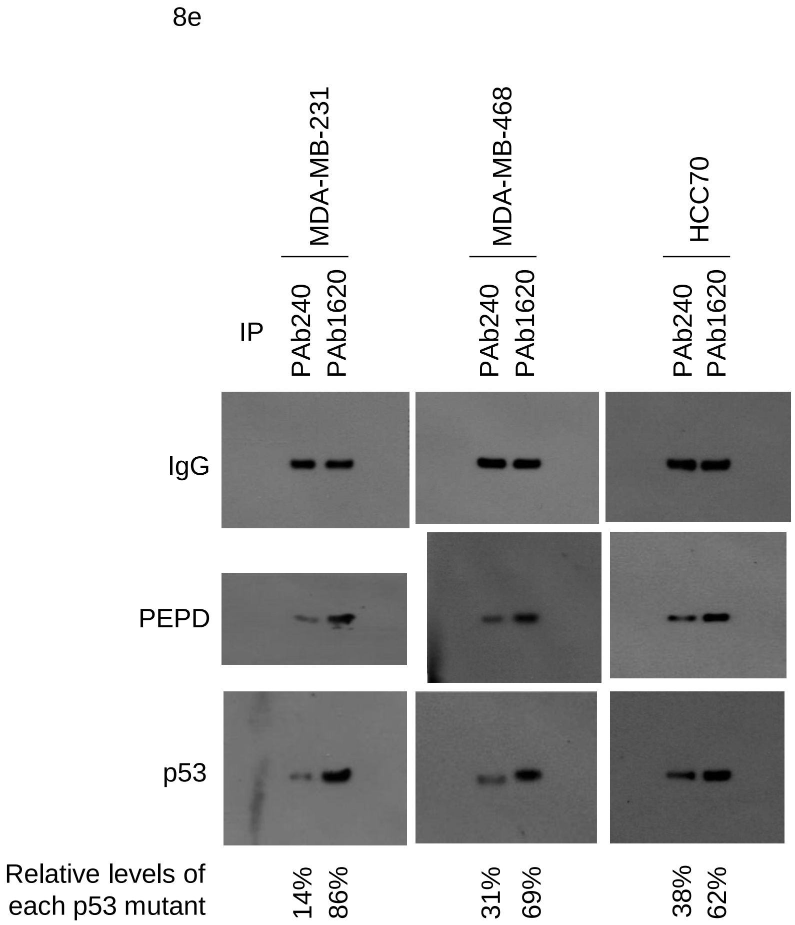

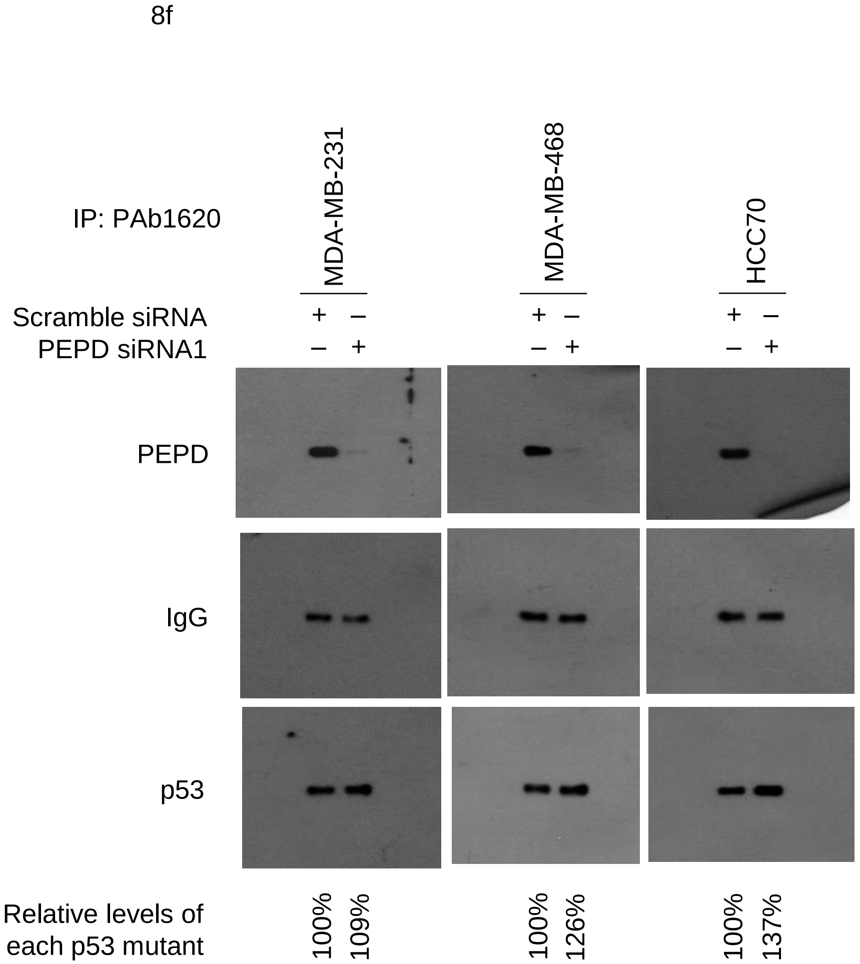

Supplementary Figure 28. Uncropped blots for Figure 8e and 8f

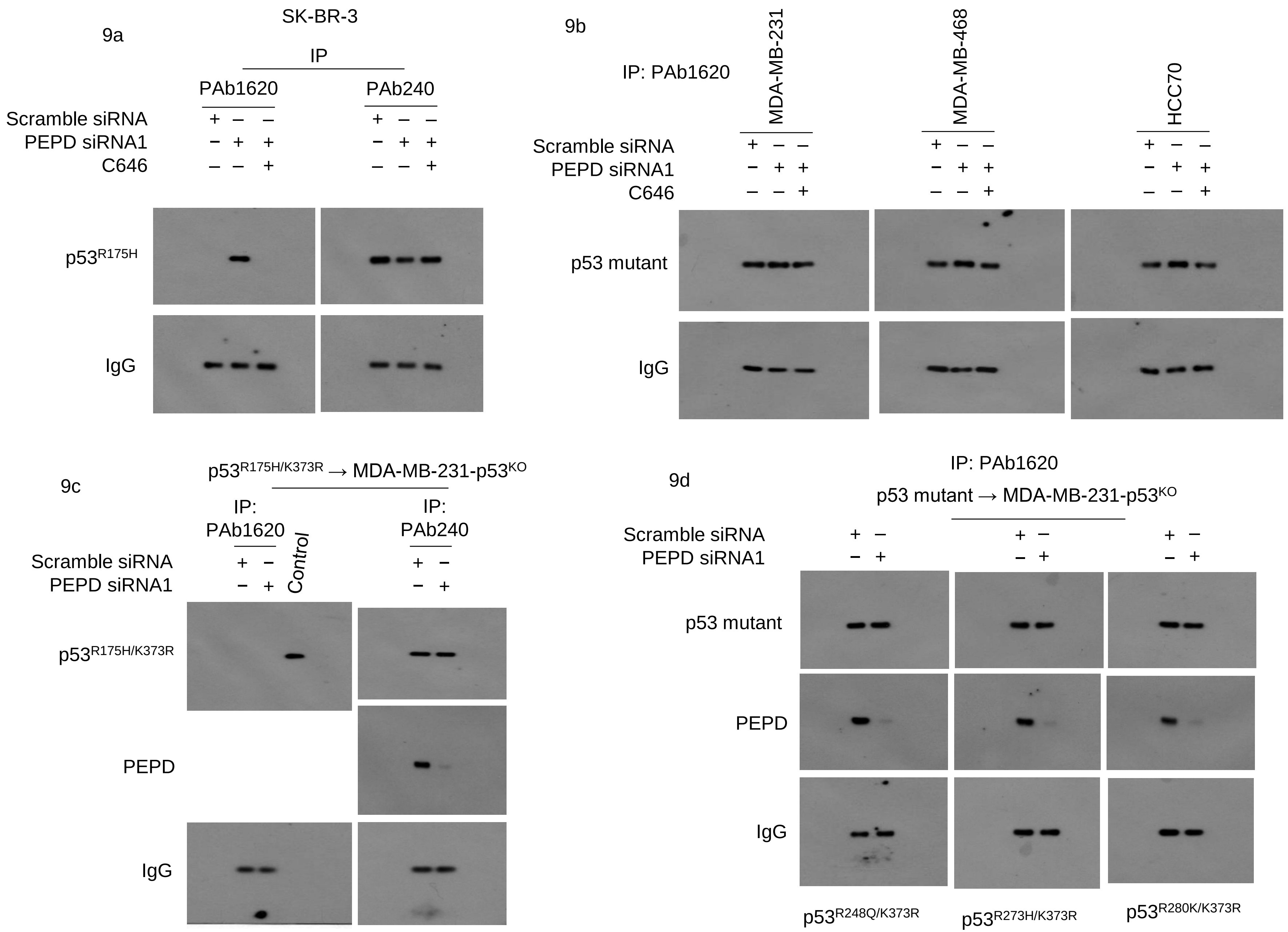

Supplementary Figure 29. Uncropped blots for Figure 9a, 9b, 9c, and 9d

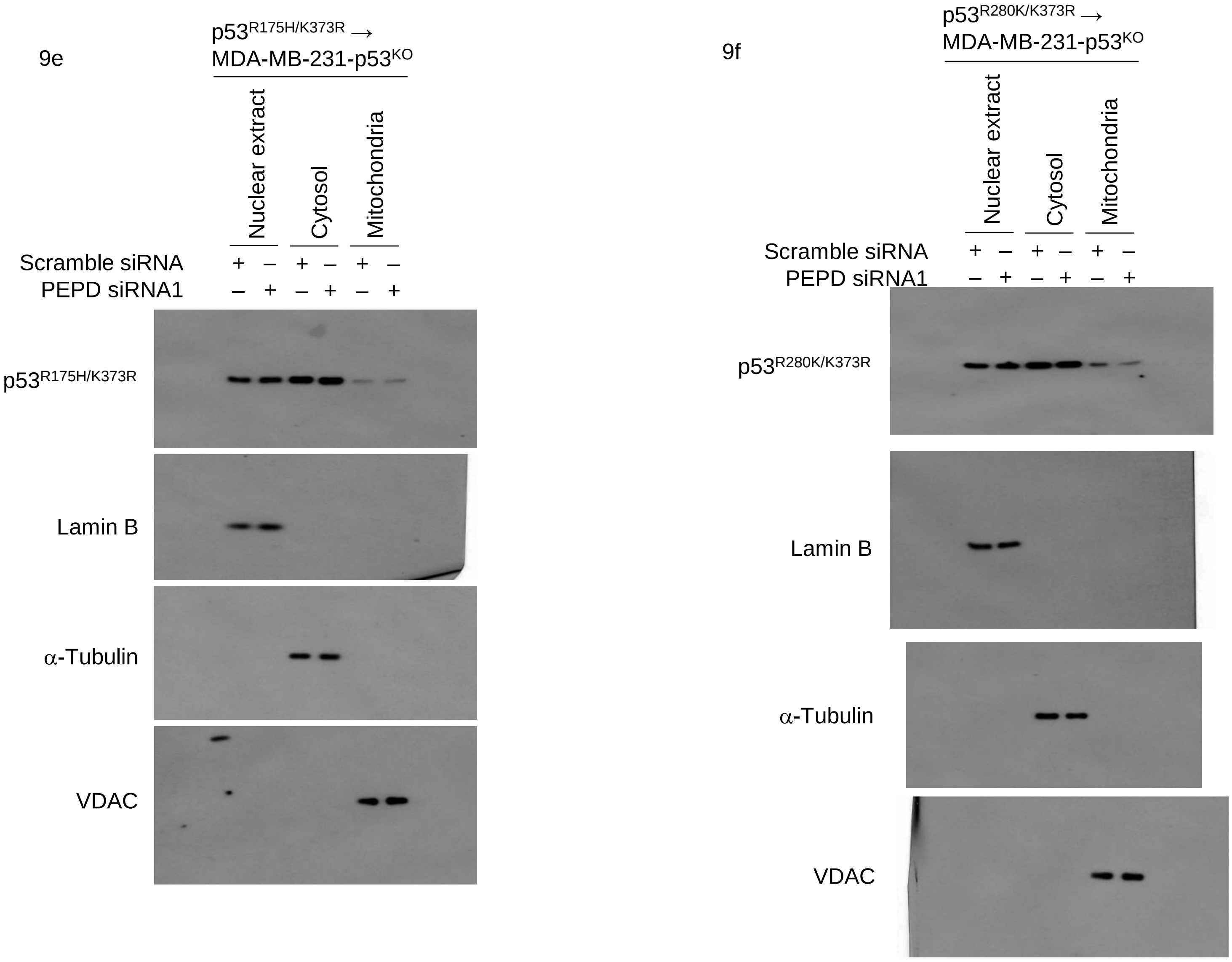

Supplementary Figure 30. Uncropped blots for Figure 9e and 9f

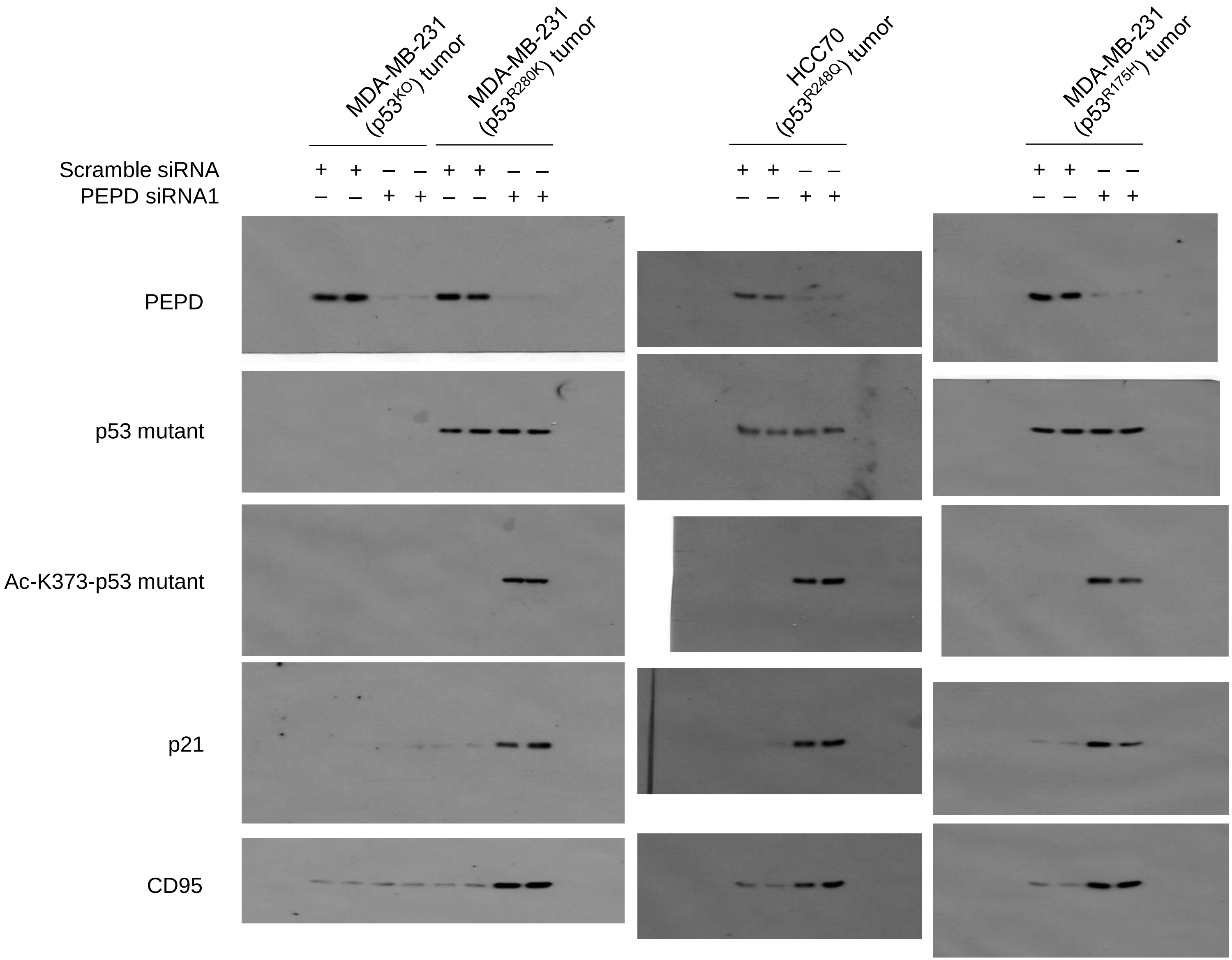

Supplementary Figure 31. Uncropped blots for Figure 10i

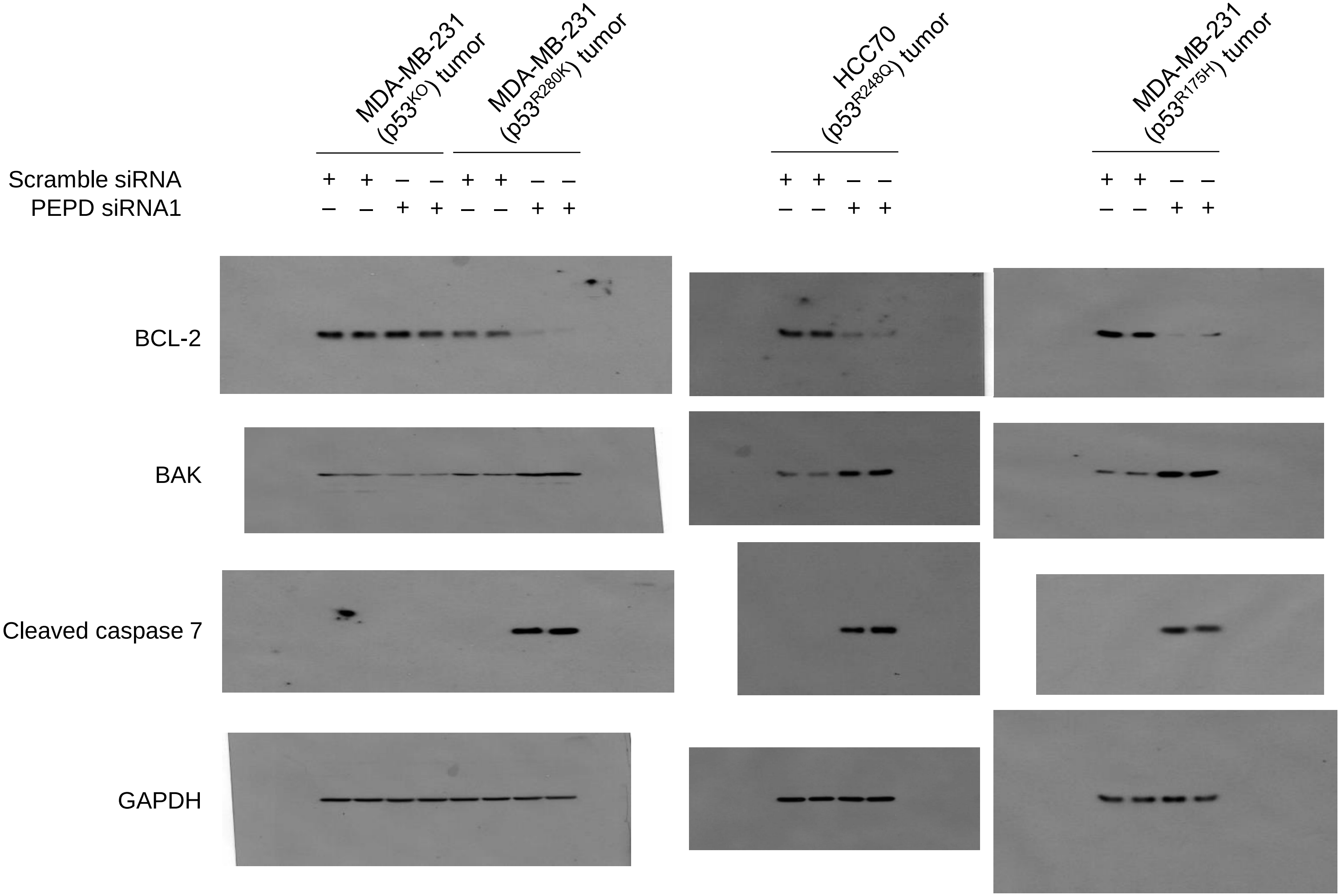

Supplementary Figure 32. Uncropped blots for Figure 10i

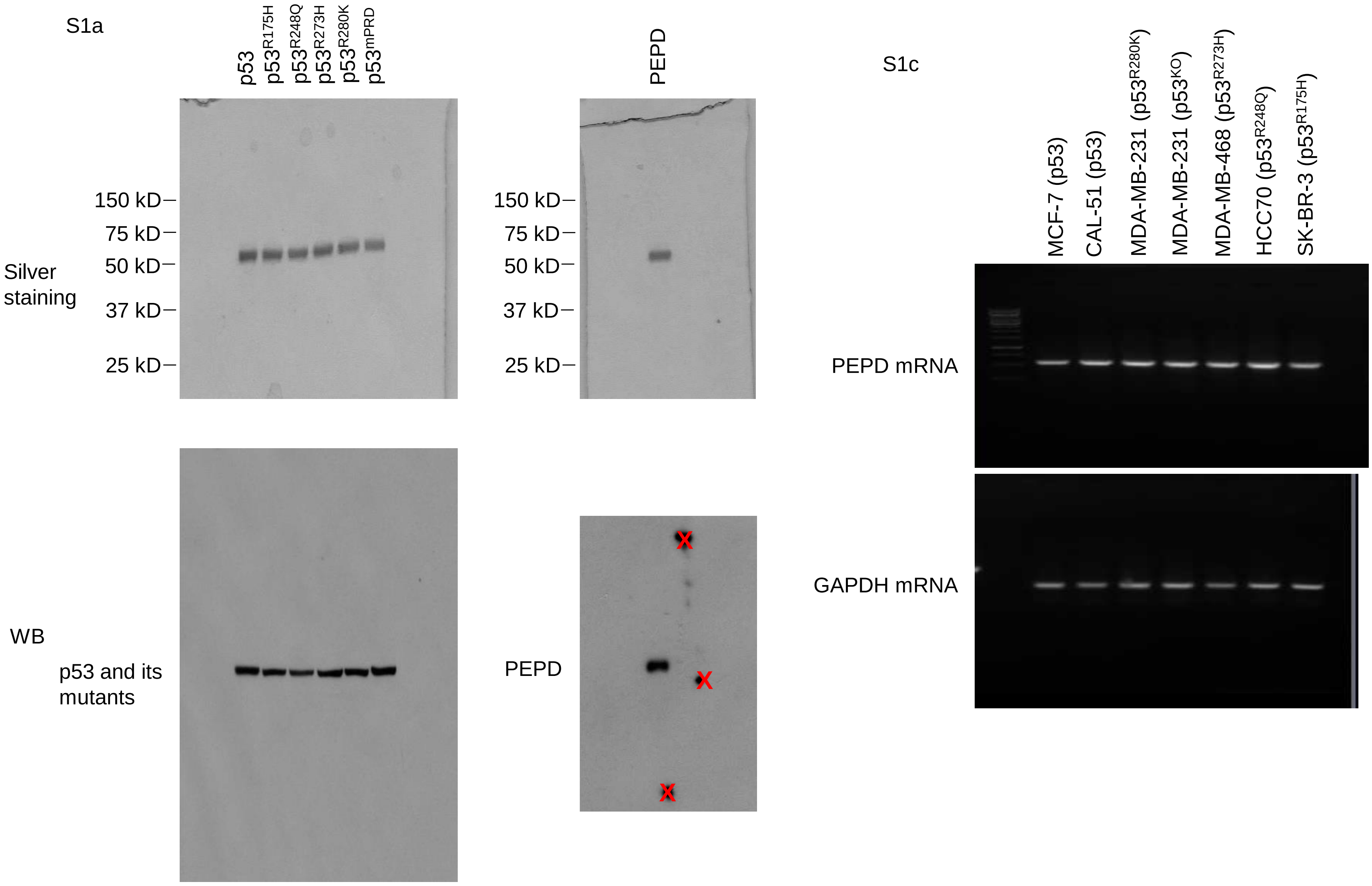

Supplementary Figure 33. Uncropped blots and gels for Figure S1a and S1c

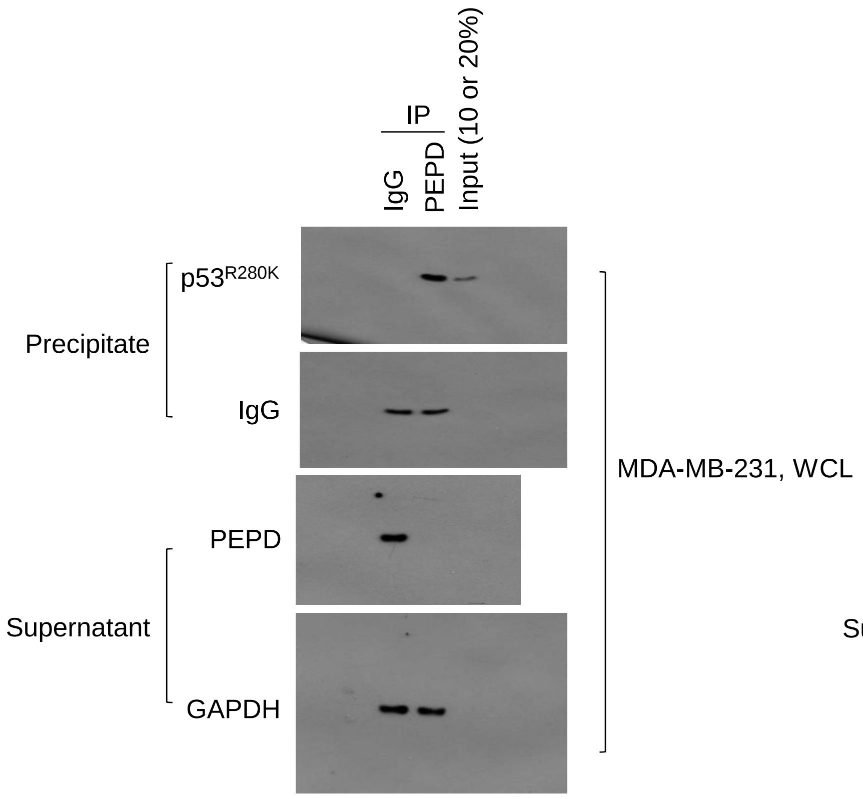

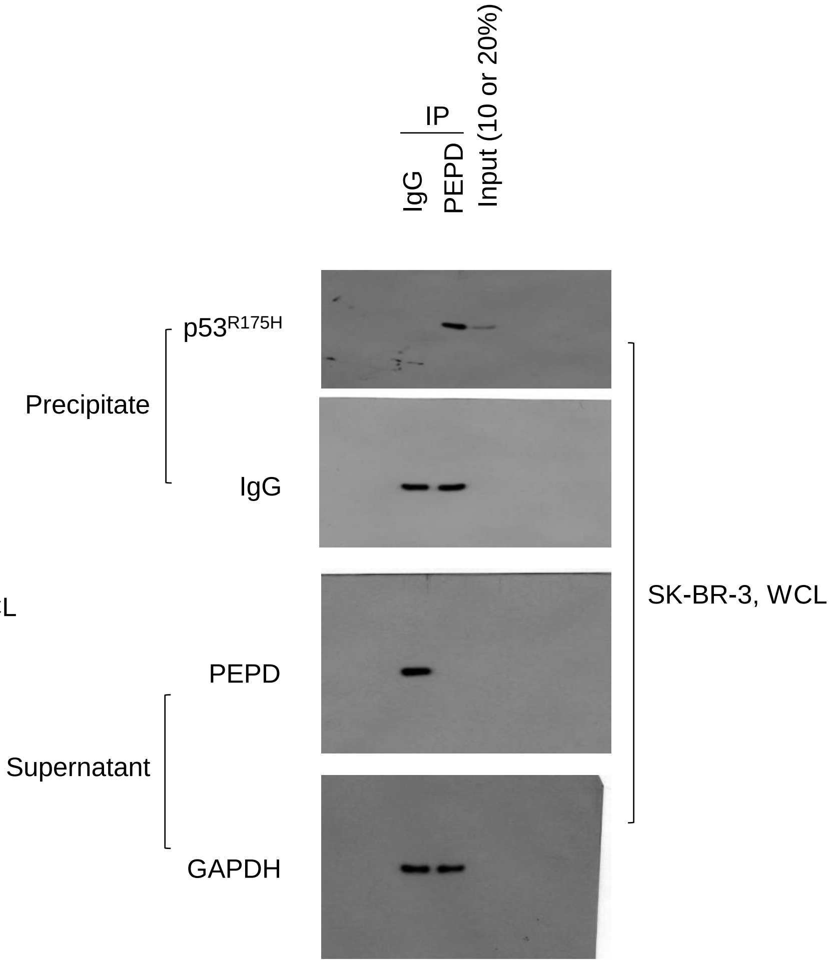

Supplementary Figure 34. Uncropped blots for Figure S2a

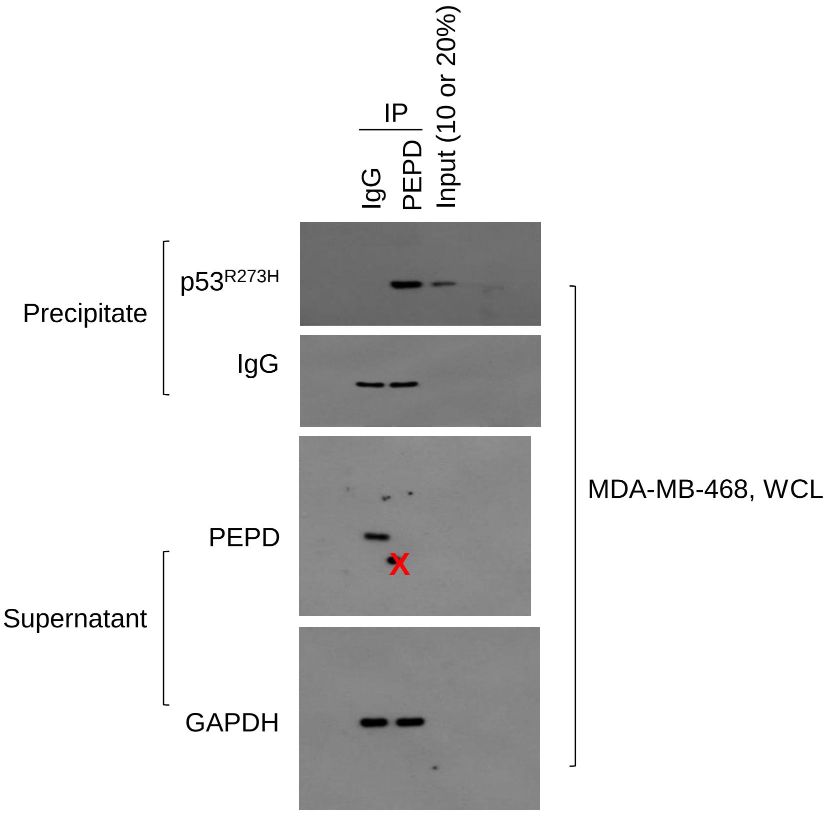

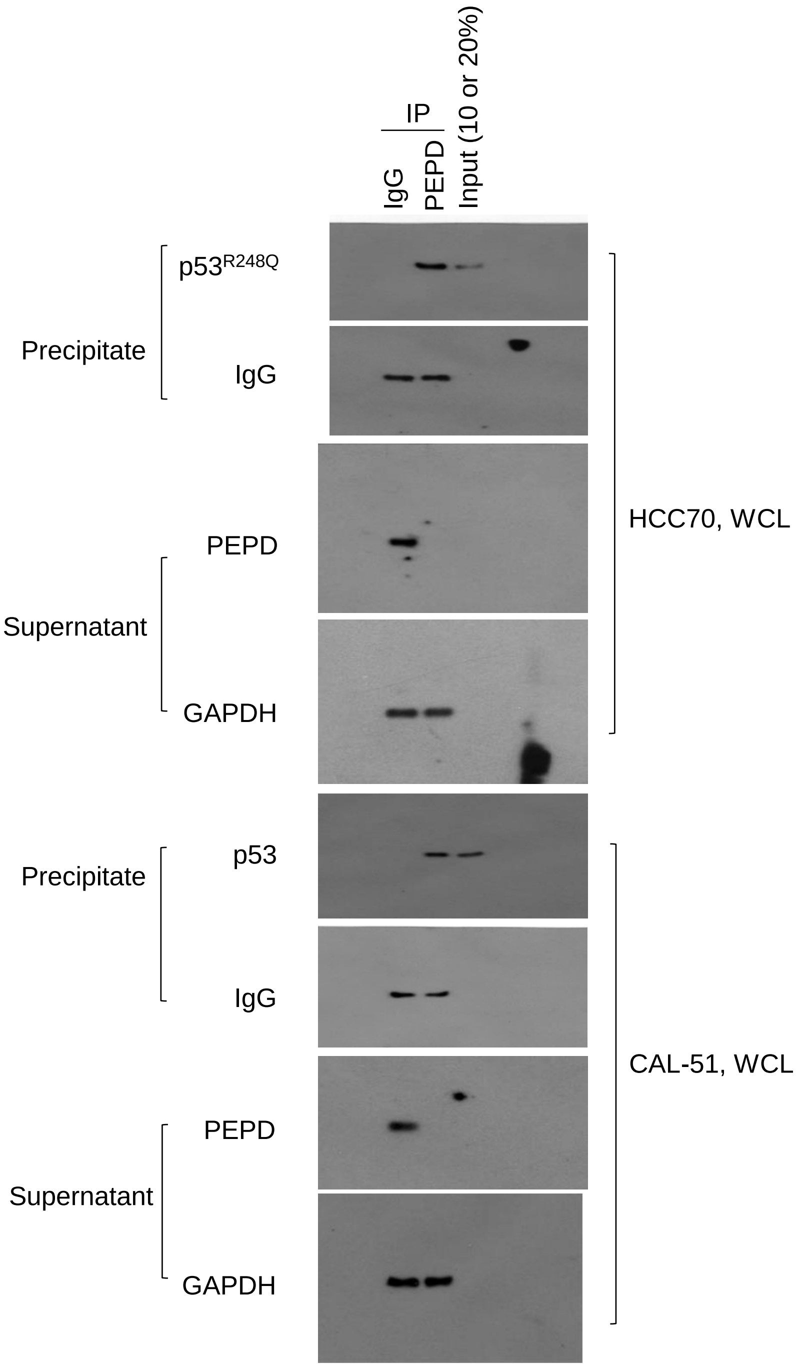

Supplementary Figure 35.
Uncropped blots for Figure S2a

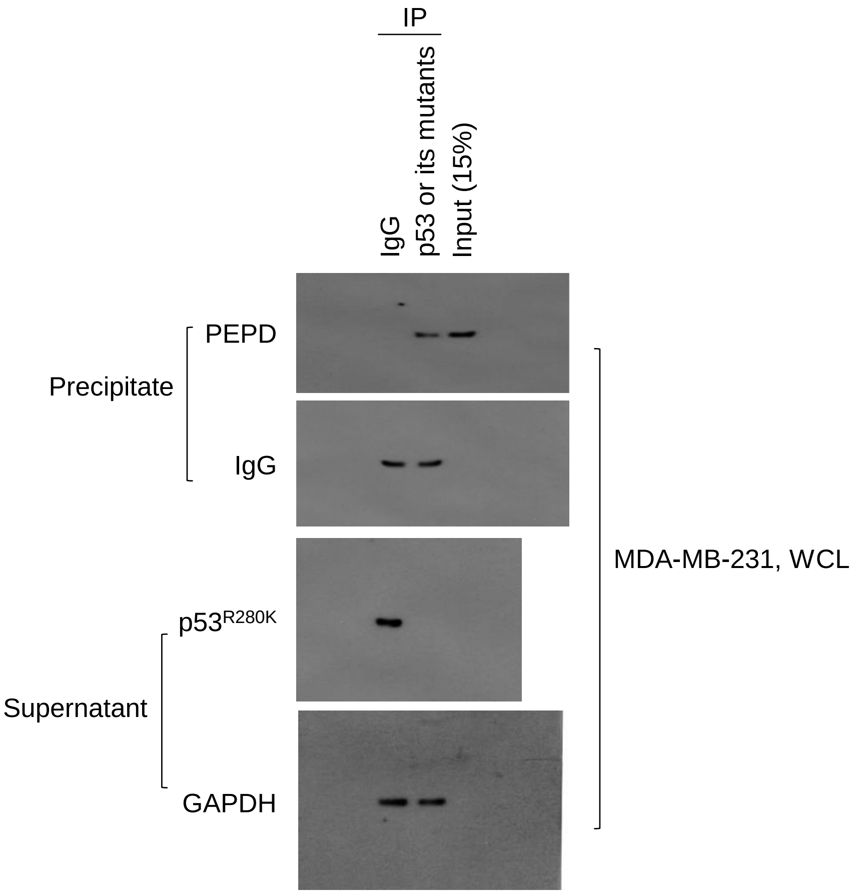

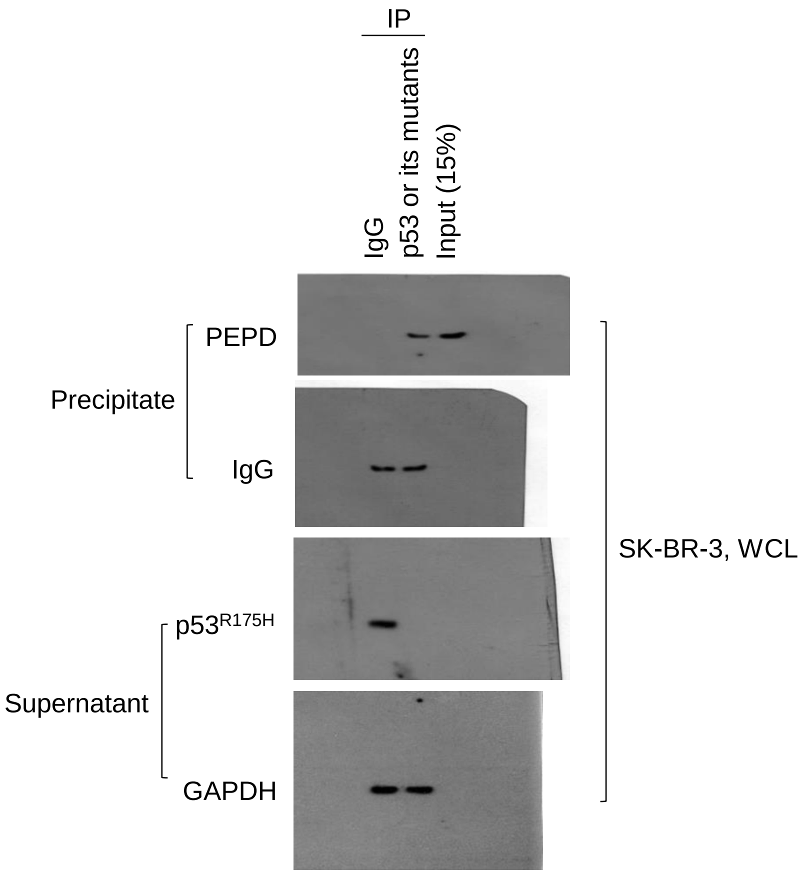

Supplementary Figure 36. Uncropped blots for Figure S2a

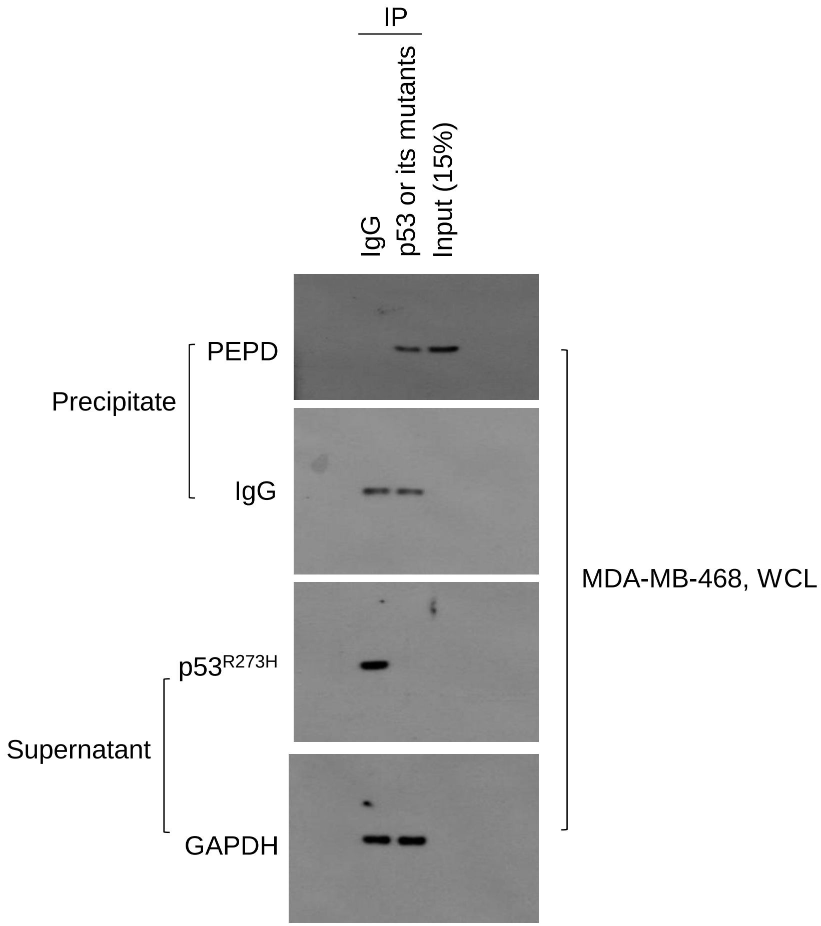

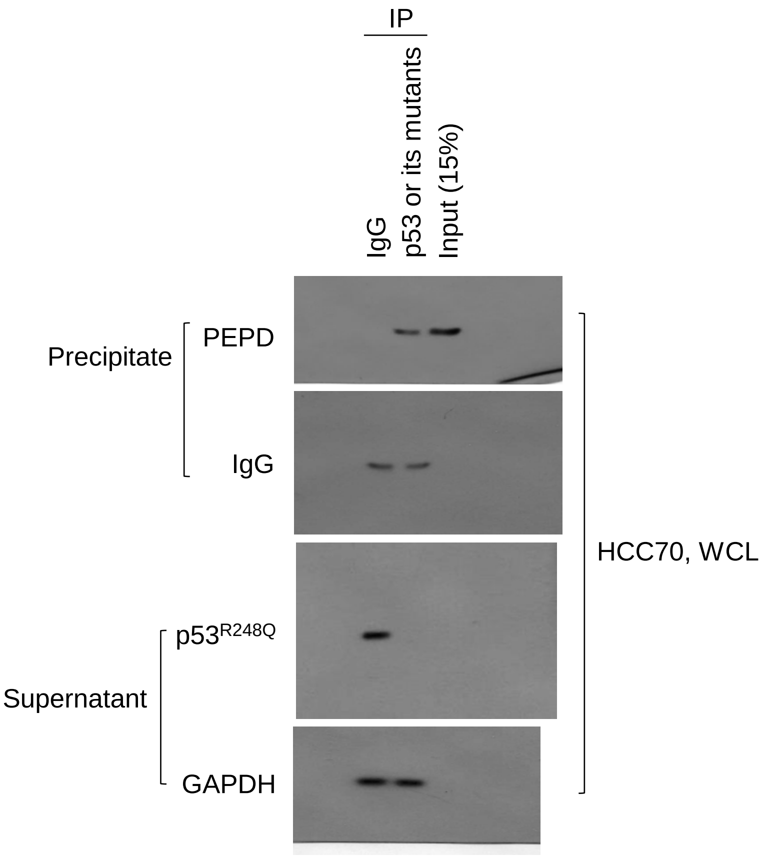

Supplementary Figure 37. Uncropped blots for Figure S2b

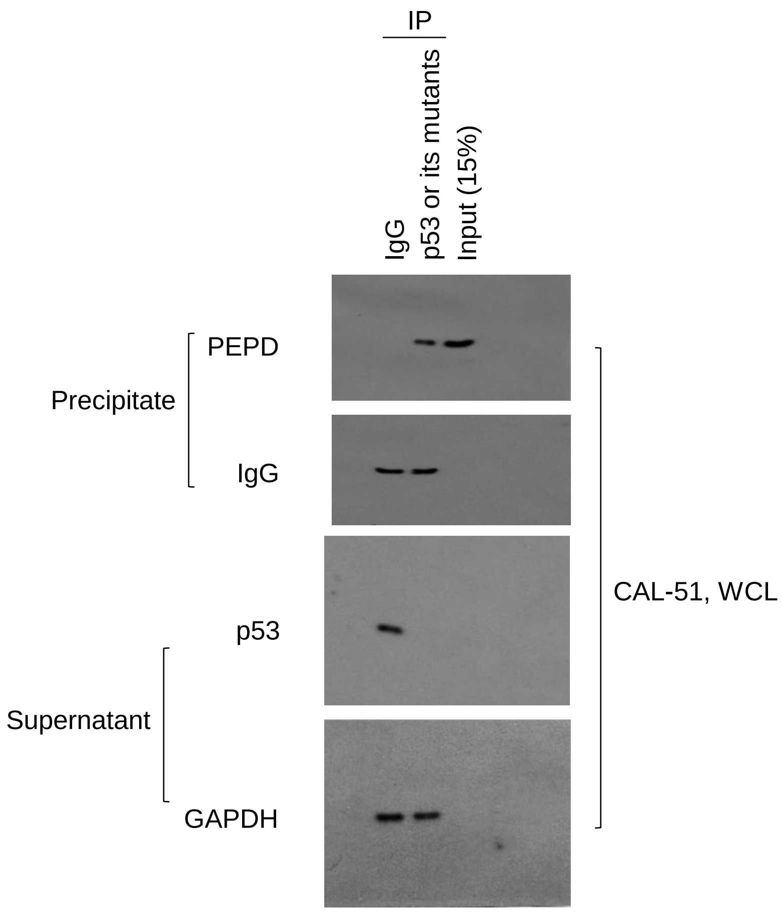

Supplementary Figure 38. Uncropped blots for Figure S2b

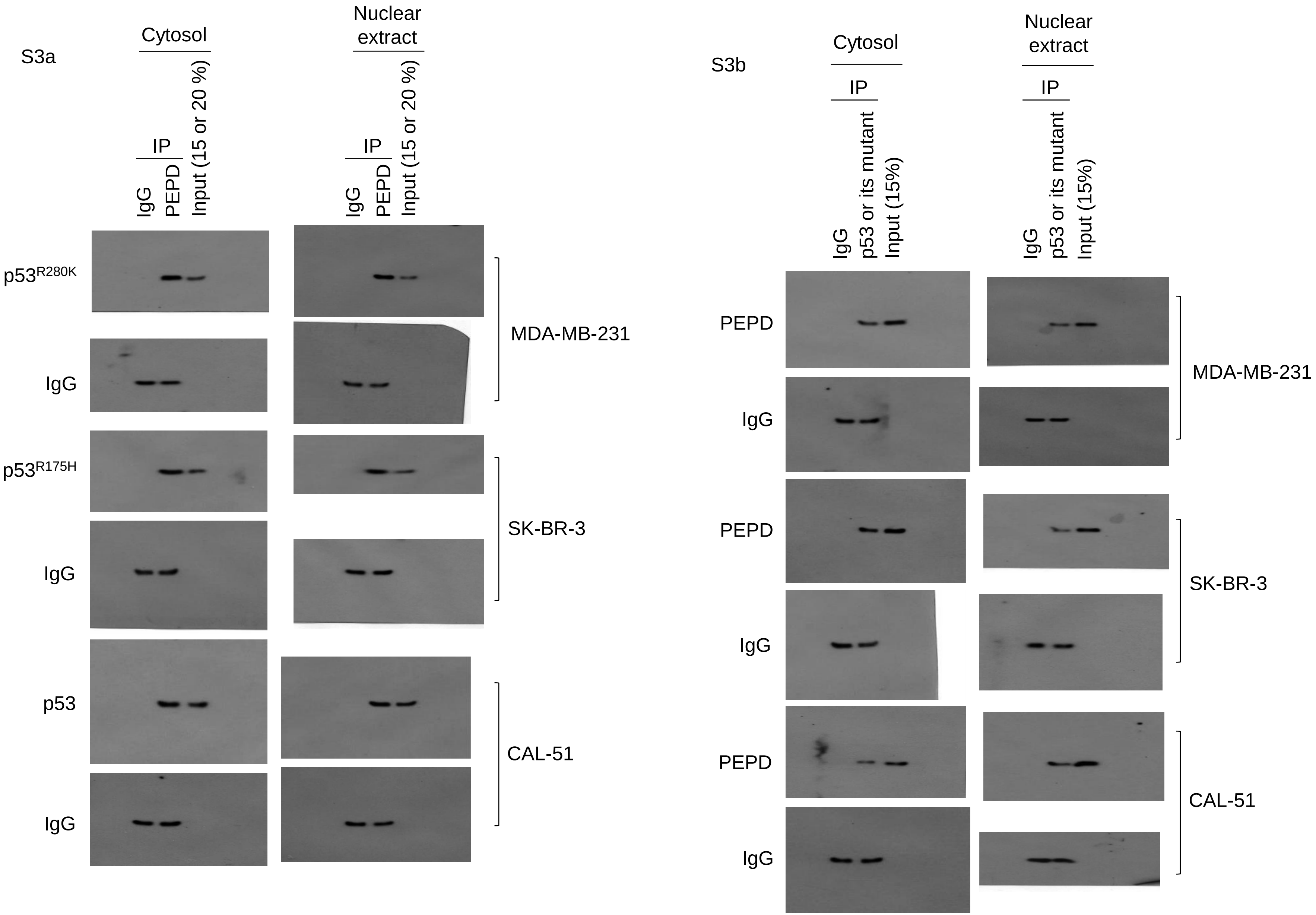

Supplementary Figure 39. Uncropped blots for Figure S3a and S3b

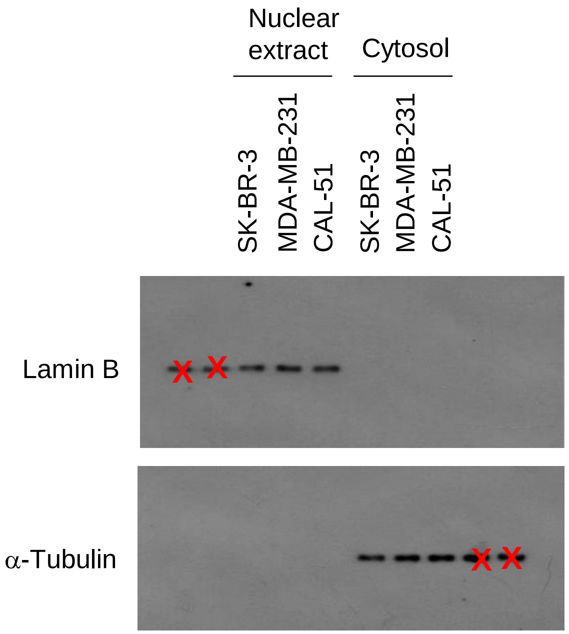

Supplementary Figure 40. Uncropped blots for Figure S3c

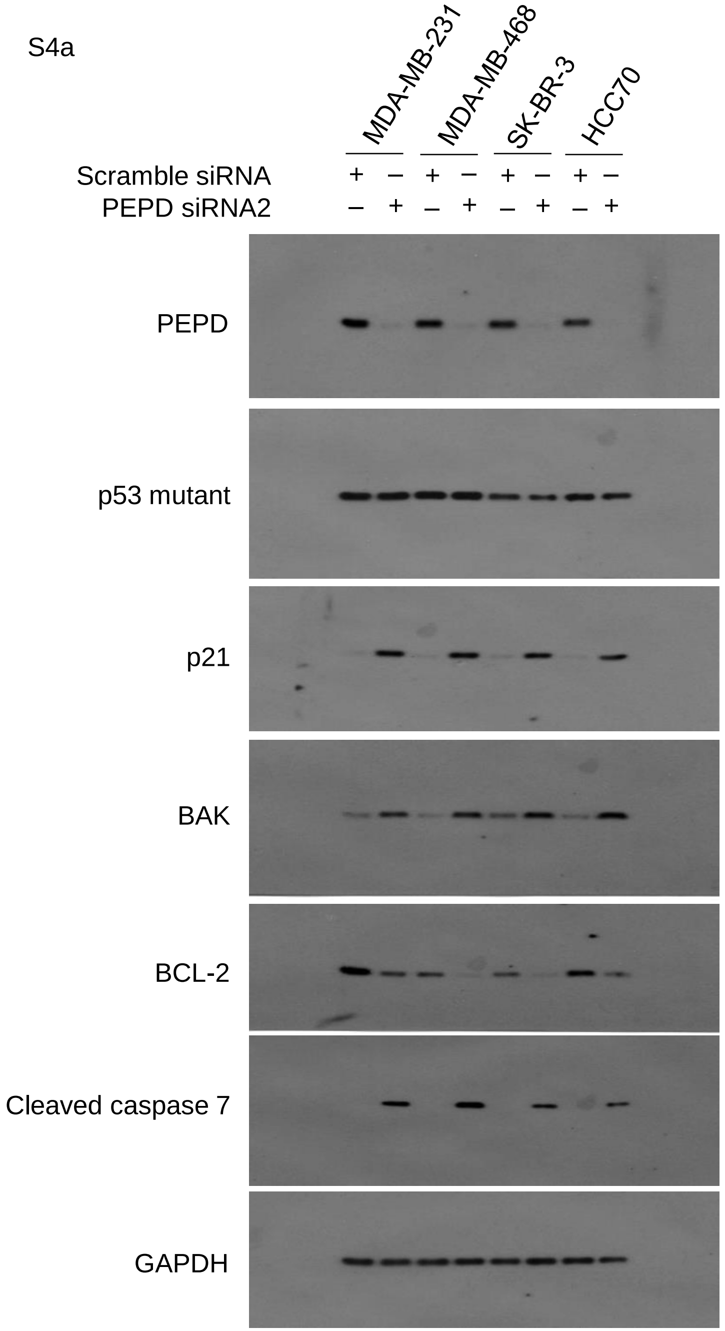

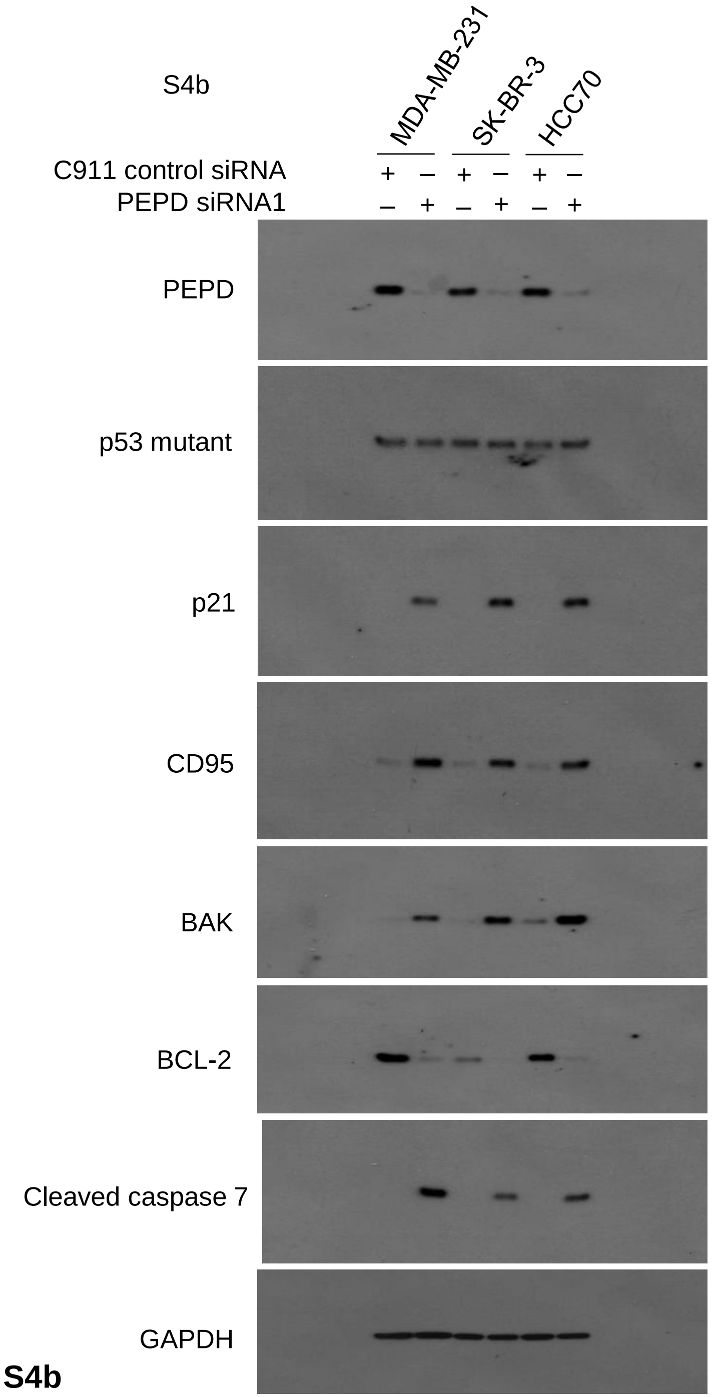

Supplementary Figure 41. Uncropped blots for Figure S4a and S4b

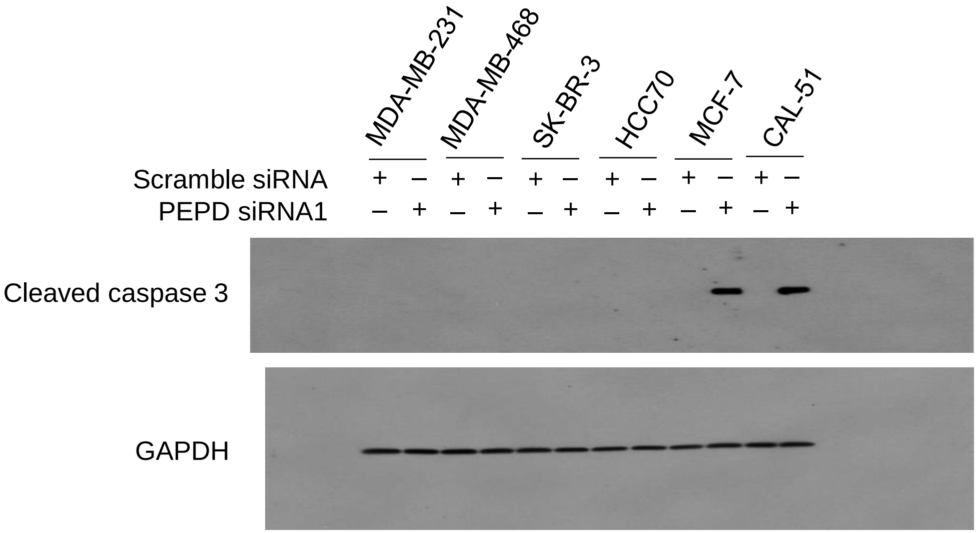

Supplementary Figure 42. Uncropped blots for Figure S4e

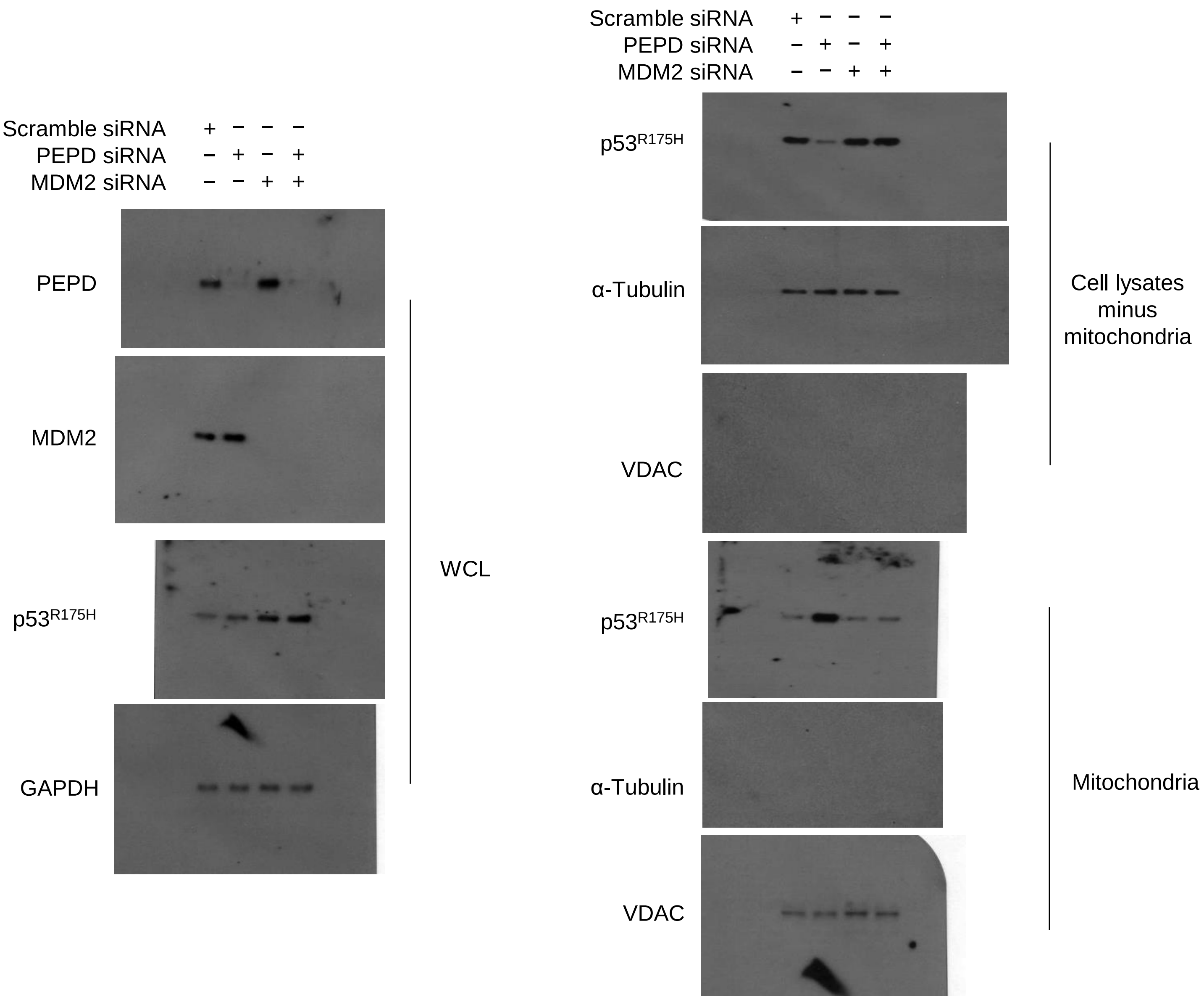

Supplementary Figure 43. Uncropped blots for Figure S6a

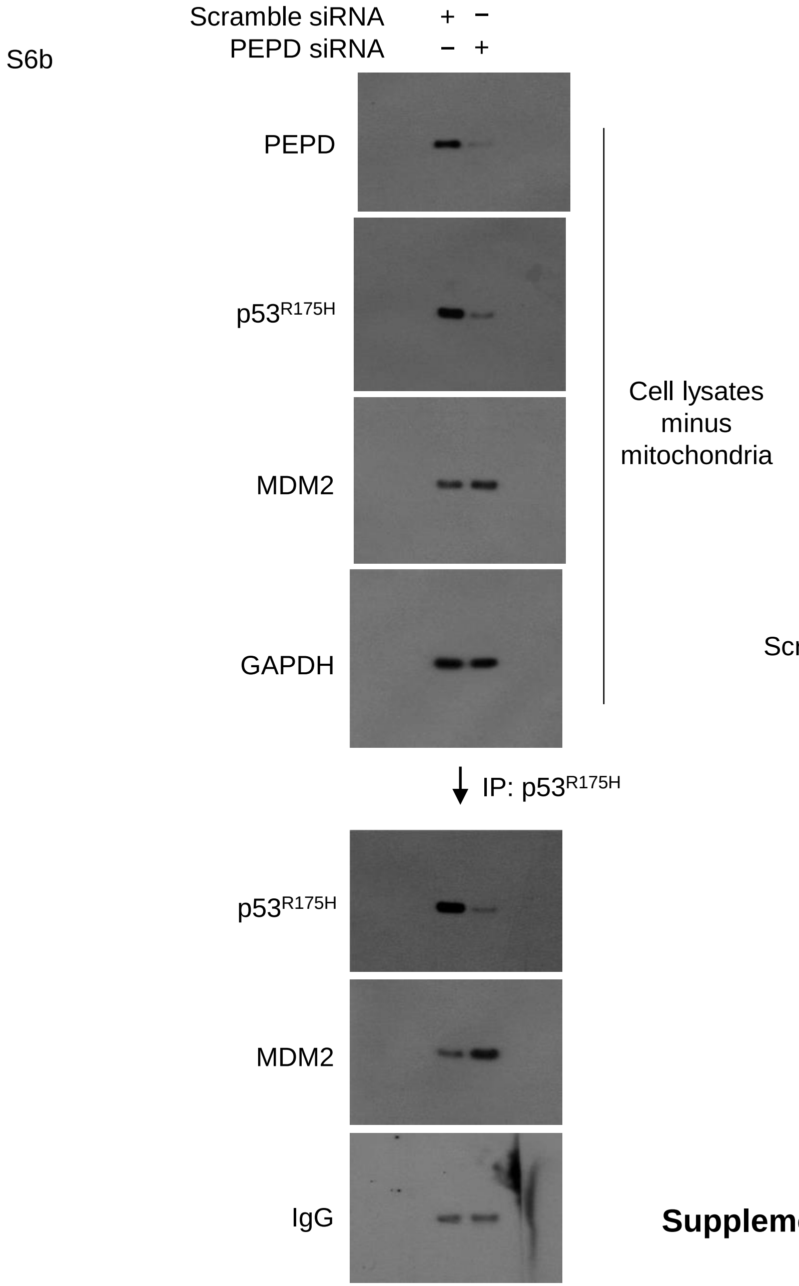

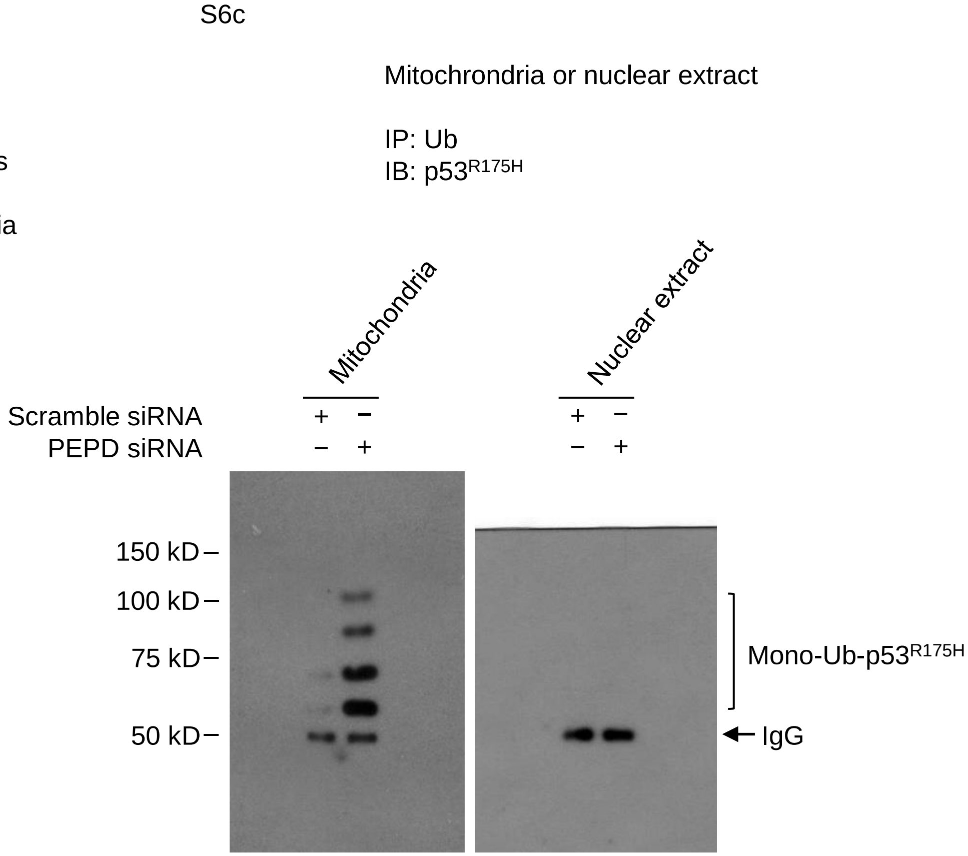

Supplementary Figure 44. Uncropped blots for Figure S6b and S6c

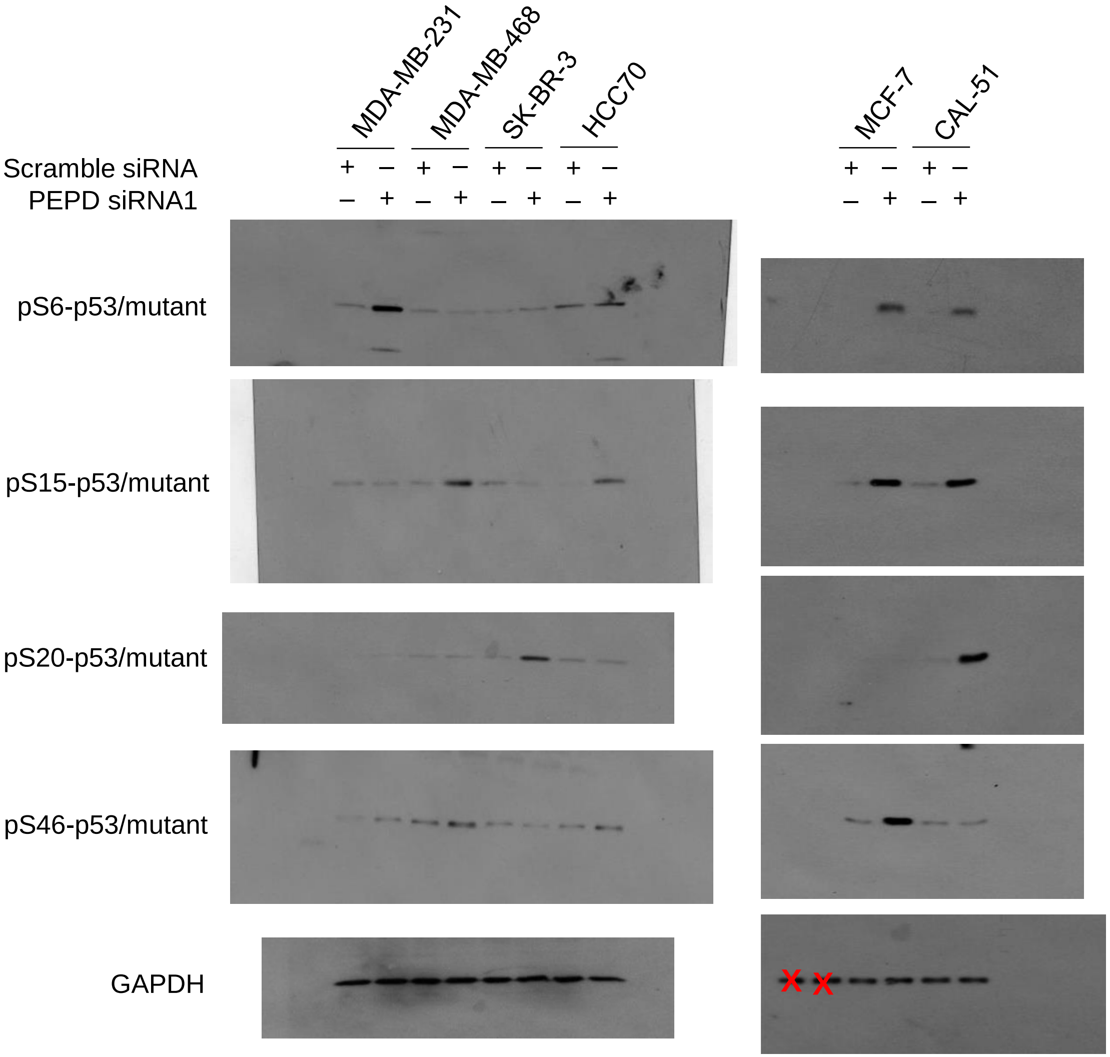

Supplementary Figure 45. Uncropped blots for Figure S7a

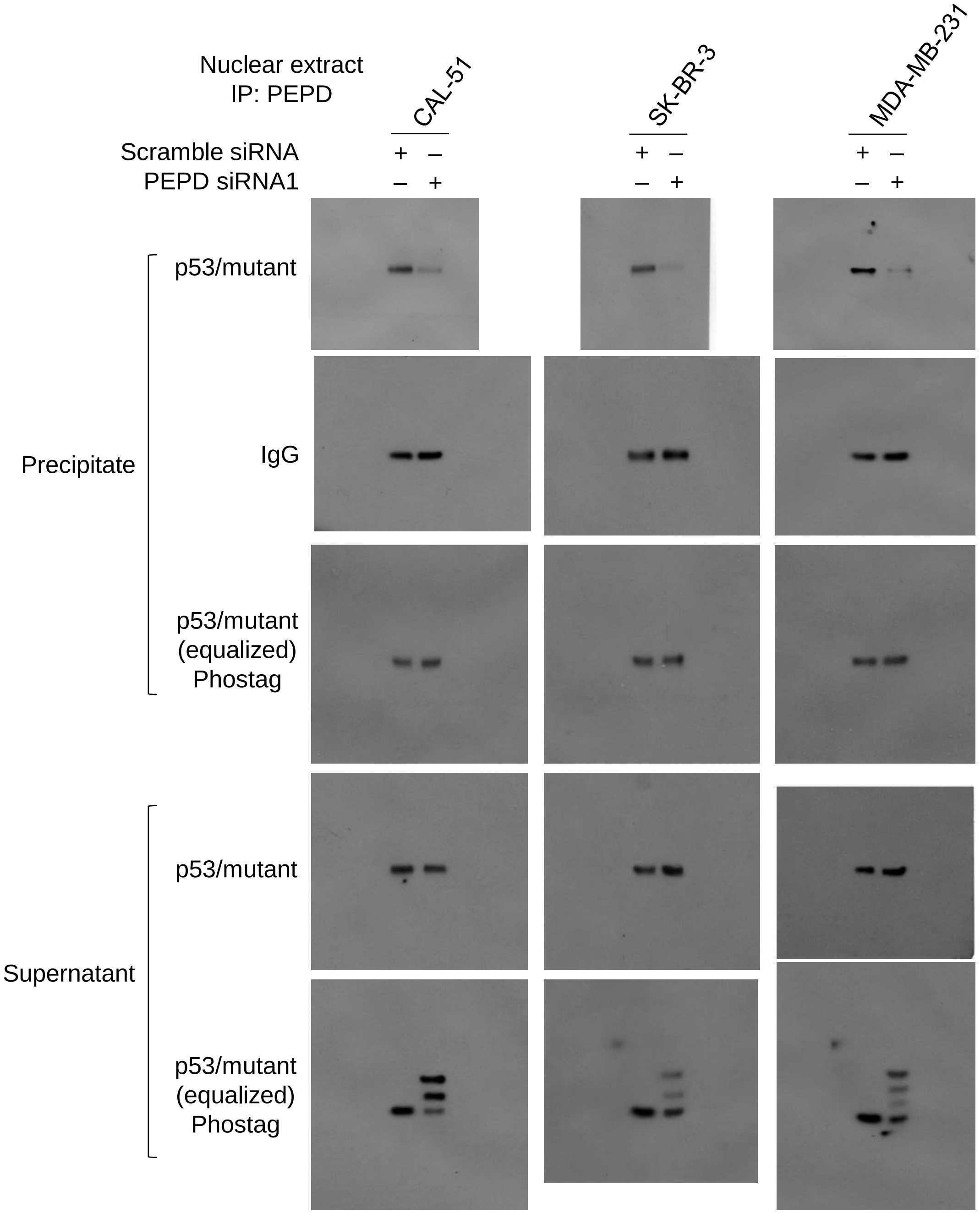

Supplementary Figure 46. Uncropped blots for Figure S7b

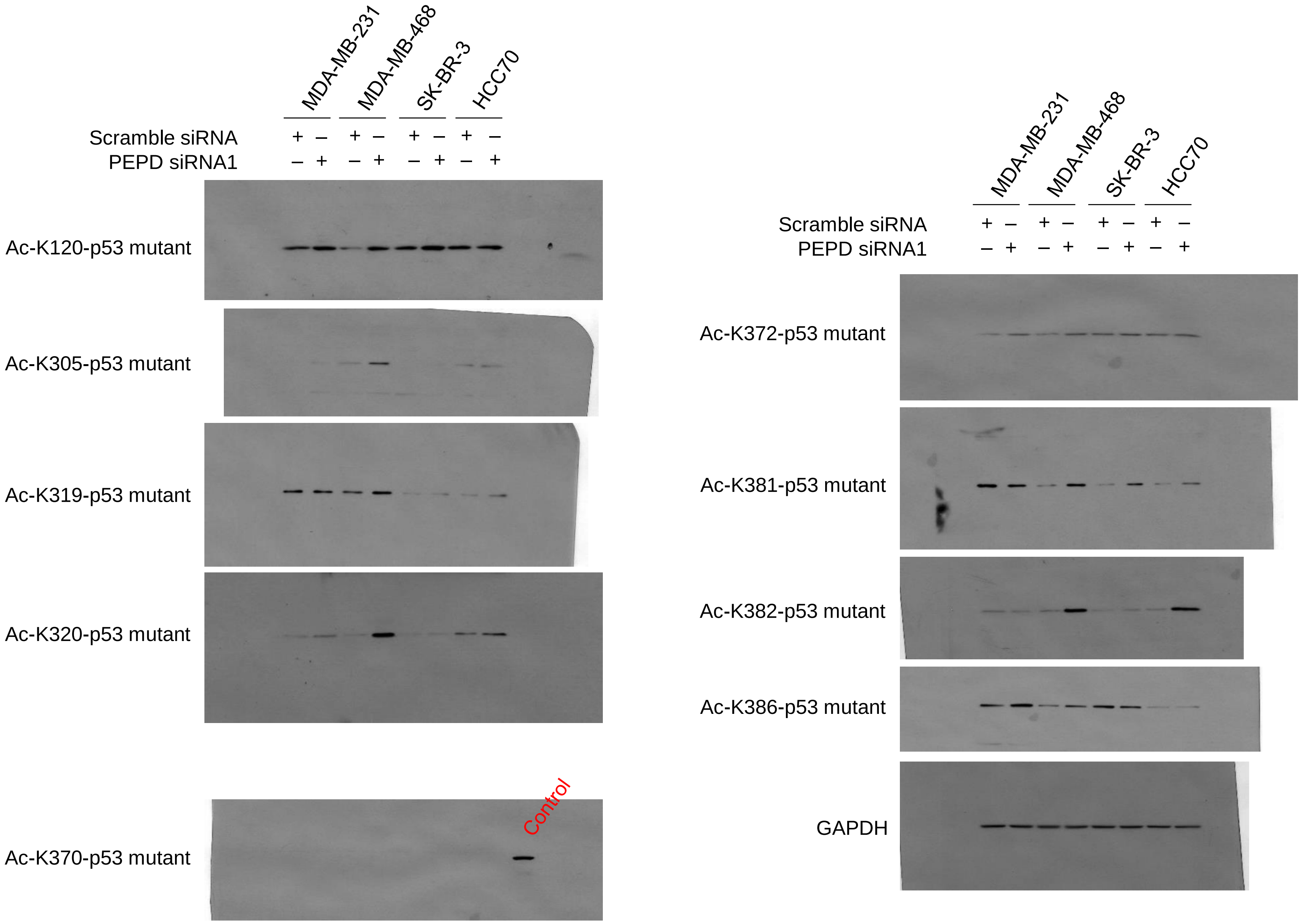

Supplementary Figure 47. Uncropped blots for Figure S8a

S8c

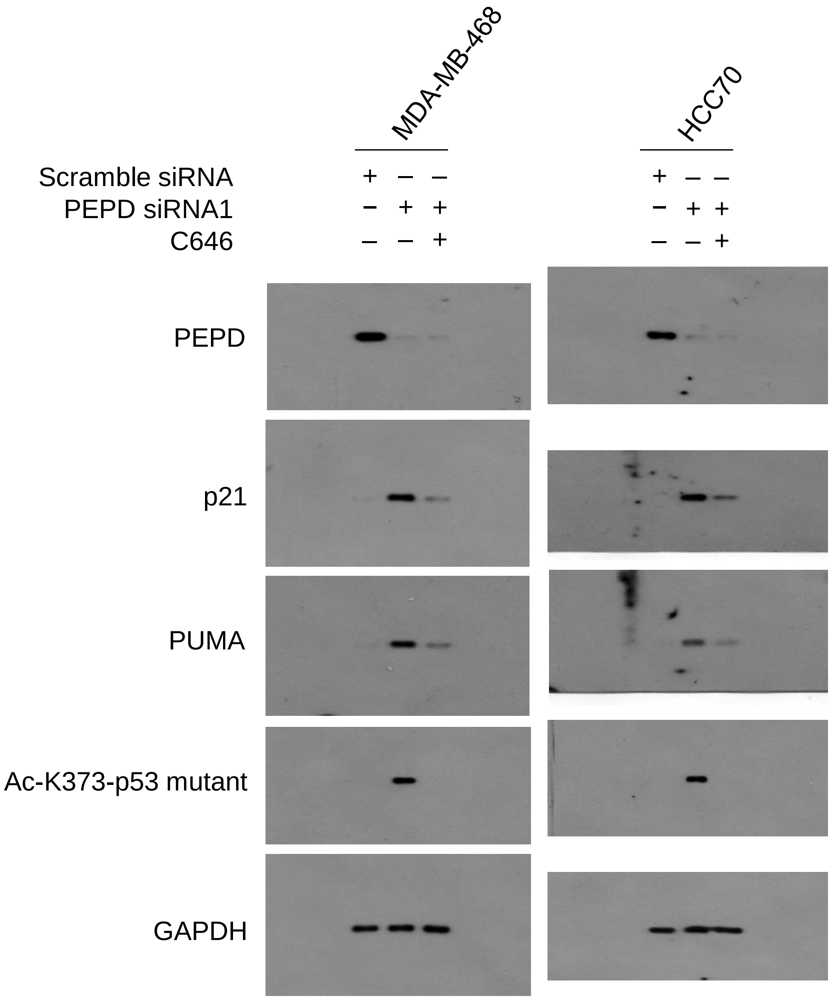

S8e

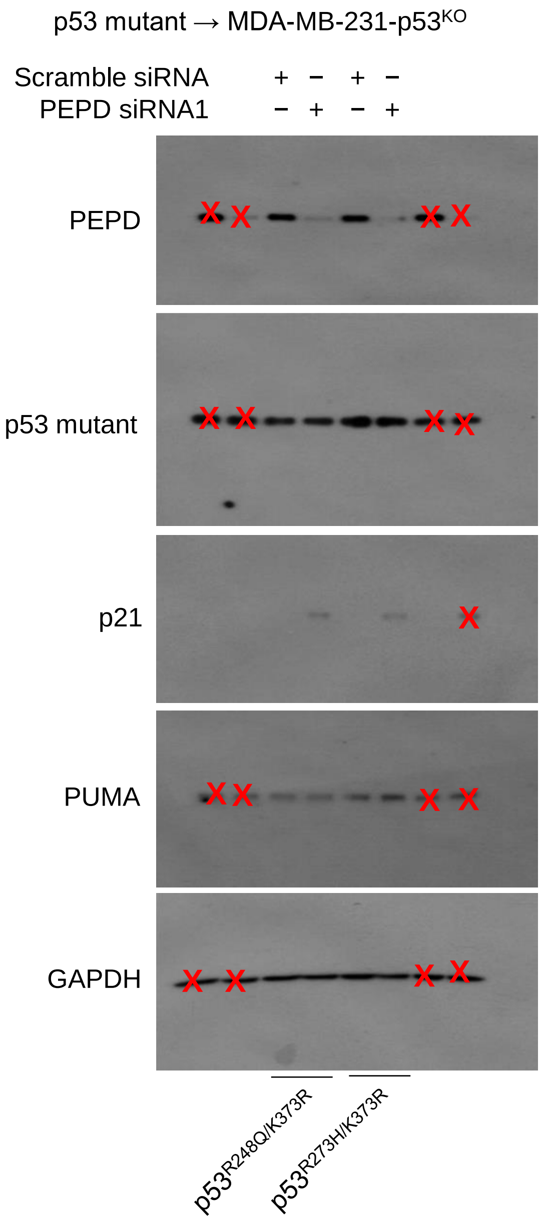

Supplementary Figure 48. Uncropped blots for Figure S8c and S8e

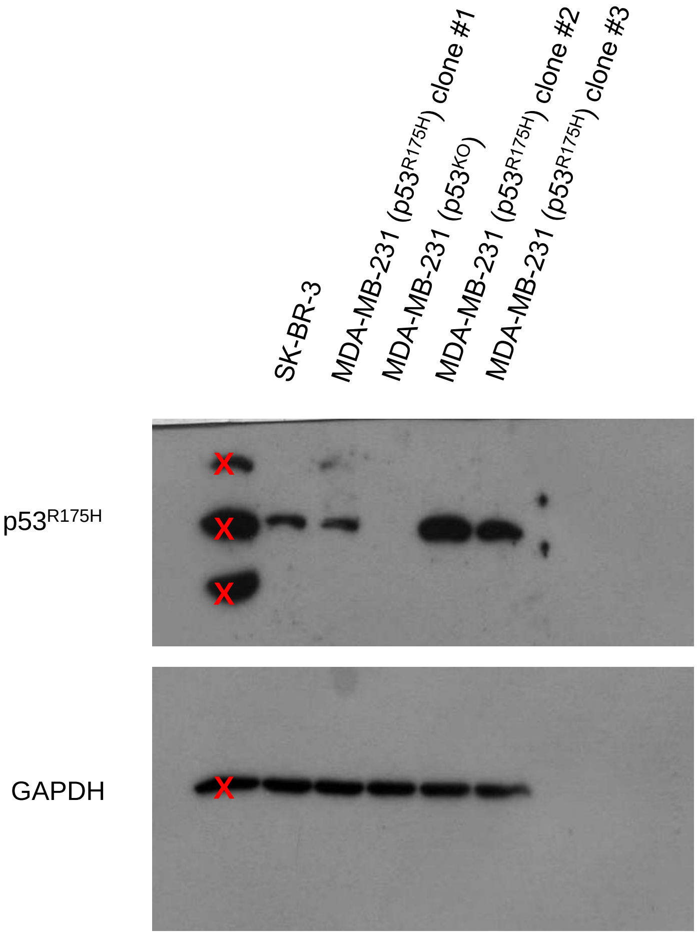

Supplementary Figure 49. Uncropped blots for Figure S9a
