# Supplementary material for: Loss of peptidase D binding restores the tumor suppressor functions of oncogenic p53 mutants
Source: Commun Biol. 2021 Dec 8;4:1373. doi: 10.1038/s42003-021-02880-x (PMC8655031; doi:10.1038/s42003-021-02880-x)
Supplement: Supplementary file 2 — Description of Additional Supplementary Files [file 42003_2021_2880_MOESM2_ESM.pdf]

Description of Additional Supplementary Files

File name: Supplementary Data.
Description:

1. Source data for Fig. 1a. Measurement of PEPD binding to p53 and p53 mutants by ELISA.
2. Source data for Fig 2b & 2c. Measurement of PEPD binding to p53 in WCL, nuclear extract, and cytosol.
3. Source data for Fig. 2d. Cellular concentrations of PEPD, p53 and p53 mutant, measured by ELISA.
4. Source data for Fig. 3a. The effect of PEPD siRNA on cell survival.
5. Source data for Fig. 3d. The effect of PEPD-G278D expression on cell death induced by PEPD siRNA.
6. Source data for Fig. 4b. The effect of PEPD siRNA on the survival of MDA-MB-231 (p53KO) cells transfected with a p53 mutant.
7. Source data for Fig. 4f. The effect of Dox withdrawal on the survival of MDA-MB-231 DKO cells transfected with Tet-on-PEPD and a p53 mutant.
8. Source data for Fig. 5b. The effect of PEPD siRNA on mitochondria membrane potential, measured by JC-1 fluorescence assay.
9. Source data for Fig. 5d. % TUNEL-positive cells induced by PEPD siRNA.
10. Source data for Fig. 6c. Effect of PEPD siRNA on transcriptional activity of p53 and p53 mutants.
11. Source data for Fig. 6d. The effect of PEPD siRNA on binding of p53 mutants to the p53 binding site in gene promoter.
12. Source data for Fig. 7d. The effect of C646 on cell growth inhibition induced by PEPD siRNA.
13. Source data for Fig. 7f and 7h. The effect of PEPD siRNA on the survival of cells expressing p53-R175H/K373R or p53-R280K/K373R
14. Source data for Fig. 10a. The effect of intratumor injection of PEPD siRNA on the growth of orthotopic MDA-MB-231 (p53-R280K) tumor.
15. Source data for Fig. 10b. The effect of intratumor injection of PEPD siRNA on the growth of orthotopic MDA-MB-231 (p53-R280K) tumor.
16. Source data for Fig. 10c. The effect of intratumor injection of PEPD siRNA on the growth of orthotopic MDA-MB-231 (p53KO) tumor.
17. Source data for Fig. 10d. The effect of intratumor injection of PEPD siRNA on the growth of orthotopic MDA-MB-231 (p53KO) tumor.
18. Source data for Fig. 10e. The effect of intratumor injection of PEPD siRNA on the growth of orthotopic HCC70 (p53-R248Q) tumor.
19. Source data for Fig. 10f. The effect of intratumor injection of PEPD siRNA on the growth of orthotopic HCC70 (p53-R248Q) tumor.
20. Source data for Fig. 10g. The effect of intratumor injection of PEPD siRNA on the growth of orthotopic MDA-MB-231 (p53-R175H) tumor.
21. Source data for Fig. 10h. The effect of intratumor injection of PEPD siRNA on the growth of orthotopic MDA-MB-231 (p53-R175H) tumor.
22. Source data for Fig. S4c. The effect of PEPD siRNA on cell survival.
23. Source data for Fig. S4d. The effect of PEPD siRNA on cell cycle progression.
24. Source data for Fig. S8b. The effect of C646 on cell growth inhibition induced by PEPD siRNA.
25. Source data for Fig. S8d. The effect of PEPD siRNA on the survival of cells expressing p53-R248Q/K373R or p53-R273H/K373R.
26. Source data for Fig. S9b. The effect of PEPD siRNA on the survival of cells expressing p53-R175H.
