# Supplementary material for: Loss of peptidase D binding restores the tumor suppressor functions of oncogenic p53 mutants
Source: Commun Biol. 2021 Dec 8;4:1373. doi: 10.1038/s42003-021-02880-x (PMC8655031; doi:10.1038/s42003-021-02880-x)
Supplement: Supplementary file 4 — Reporting Summary [file 42003_2021_2880_MOESM4_ESM.pdf]

nature research

Corresponding author(s): Yuesheng Zhang
Last updated by author(s): October 1, 2021

Reporting Summary

Nature Research wishes to improve the reproducibility of the work that we publish. This form provides structure for consistency and transparency in reporting. For further information on Nature Research policies, see our Editorial Policies and the Editorial Policy Checklist.

Statistics

For all statistical analyses, confirm that the following items are present in the figure legend, table legend, main text, or Methods section.

n/a	Confirmed	
☐	☒	The exact sample size (*n*) for each experimental group/condition, given as a discrete number and unit of measurement
☐	☒	A statement on whether measurements were taken from distinct samples or whether the same sample was measured repeatedly
☐	☒	The statistical test(s) used AND whether they are one- or two-sided<br>*Only common tests should be described solely by name; describe more complex techniques in the Methods section.*
☒	☐	A description of all covariates tested
☒	☐	A description of any assumptions or corrections, such as tests of normality and adjustment for multiple comparisons
☐	☒	A full description of the statistical parameters including central tendency (e.g. means) or other basic estimates (e.g. regression coefficient) AND variation (e.g. standard deviation) or associated estimates of uncertainty (e.g. confidence intervals)
☐	☒	For null hypothesis testing, the test statistic (e.g. *F*, *t*, *r*) with confidence intervals, effect sizes, degrees of freedom and *P* value noted<br>*Give P values as exact values whenever suitable.*
☒	☐	For Bayesian analysis, information on the choice of priors and Markov chain Monte Carlo settings
☒	☐	For hierarchical and complex designs, identification of the appropriate level for tests and full reporting of outcomes
☒	☐	Estimates of effect sizes (e.g. Cohen's *d*, Pearson's *r*), indicating how they were calculated

Our web collection on statistics for biologists contains articles on many of the points above.

Software and code

Policy information about availability of computer code

Data collection	N/A
Data analysis	N/A

For manuscripts utilizing custom algorithms or software that are central to the research but not yet described in published literature, software must be made available to editors and reviewers. We strongly encourage code deposition in a community repository (e.g. GitHub). See the Nature Research guidelines for submitting code & software for further information.

Data

Policy information about availability of data

All manuscripts must include a data availability statement. This statement should provide the following information, where applicable:

- Accession codes, unique identifiers, or web links for publicly available datasets
- A list of figures that have associated raw data
- A description of any restrictions on data availability

The authors declare that all the data supporting the findings of this study are available within the article and its supplementary information file and from the corresponding author upon reasonable request.

Field-specific reporting

Please select the one below that is the best fit for your research. If you are not sure, read the appropriate sections before making your selection.

☒ Life sciences ☐ Behavioural & social sciences ☐ Ecological, evolutionary & environmental sciences

For a reference copy of the document with all sections, see nature.com/documents/nr-reporting-summary-flat.pdf

Life sciences study design

All studies must disclose on these points even when the disclosure is negative.

Sample size	Each non-animal experiment involving statistical analysis was performed three times, to enable adequate statistical analysis. The information is provided in each figure legend. Sample size in the mouse experiments was estimated on the basis of our published study on tumors carrying p53. Sample size of 13-16 per group was used in each mouse experiment.
Data exclusions	No data were excluded.
Replication	All cell-based experiment were repeated at least twice. For experiments involving statistical analysis, each experiment was repeated three times. All attempts at replication were successful. Animal experiments were not repeated, but a large group sample size was used in each experiment.
Randomization	Randomization was used in all mouse experiments, using Research Randomizer (www.randomizer.org). Cell line experiments were not randomized but experimental treatments were assigned without any bias.
Blinding	Investigators were not blinded during experimental treatment and data collection. Only cell lines and mice were used in the study. Blinding was not feasible because usually one investigator was performing a particular experiment.

Reporting for specific materials, systems and methods

We require information from authors about some types of materials, experimental systems and methods used in many studies. Here, indicate whether each material, system or method listed is relevant to your study. If you are not sure if a list item applies to your research, read the appropriate section before selecting a response.

Materials & experimental systems

n/a	Involved in the study	
☐	☒	Antibodies
☐	☒	Eukaryotic cell lines
☒	☐	Palaeontology and archaeology
☐	☒	Animals and other organisms
☒	☐	Human research participants
☒	☐	Clinical data
☒	☐	Dual use research of concern

Methods

n/a	Involved in the study	
☒	☐	ChIP-seq
☐	☒	Flow cytometry
☒	☐	MRI-based neuroimaging

Antibodies

Antibodies used	Anti-acetyl-p53 (K120) (Cat# ab78316), anti-acetyl-p53 (K305) (Cat# ab109396), anti-acetyl-p53 (K386) (Cat# ab52172), and anti-PEPD (Cab# ab86507 and ab197890) were purchased from Abcam. Anti-acetyl-p53 (K372) (Cat# A96486) was purchased from Antibody.com. Anti-BAK (Cat# 3792), anti-BAX (Cat# 2772), anti-BCL-2 (Cat# 2870), anti-BCL-XL (Cat# 2764), anti-BID (Cat# 2002), anti-alpha-tubulin (cat#86298), anti-cleaved caspase-3 (Cat# 9661), anti-cleaved caspase-7 (Cat# 9491), anti-cleaved caspase-8 (Cat# 9496), anti-cleaved caspase-9 (Cat# 9501), anti-Cyto c (Cat# 4272), anti-EGFR (Cat# 2232), anti-EndoG (Cat# 4969), anti-MKK3 (Cat# 8535), anti-MYC (Cat# 9402), anti-p53 (Cat# 2524, 2527 and 18032), anti-phospho-p53 (S6) (Cat#9285), anti-phospho-p53 (S15) (Cat# 9284), anti-phospho-p53 (S20) (Cat# 9287), anti-phospho-p53 (S46) (Cat#2521), anti-p21 (Cat# 2946), and anti-VDAC (Cat# 4866) were purchased from Cell Signaling Technology. Anti-acetyl-p53 (K320) (Cat# 06-1283), anti-GAPDH (Cat# MAB374), PAb1620 (Cat# OP33-20UG), and PAb240 (Cat# OP29-100UG) were purchased from Millipore. Anti-AIF (Cat# sc-13116), anti-lamin B (Cat# sc-6216), anti-MDM2 (Cat# sc-965), anti-PUMA (sc-28226), and anti-ubiquitin (Cat# sc-8017) were purchased from Santa Cruz Biotechnology. Anti-acetyl-p53 (K319) (Cat# PA5-99334), anti-acetyl-p53 (K370) (Cat# MA5-32007), anti-acetyl-p53 (K373) (Cat# PA5-105109), anti-acetyl-p53 (K381) (Cat# PA5-105110), anti-acetyl-p53 (K382) (Cat# 710294), anti-CYPD (Cat# PA5-80923), and anti-6x-His tag (Cat# MA1-21315) were purchased from Thermo Fisher Scientific. Sheep anti-mouse IgG-HRP (Cat# NA931) and donkey anti-rabbit IgG-HRP (NA934) were purchased from GE Healthcare. Goat anti-rabbit IgG (Cat# 111-035-008) and goat anti-mouse IgG (Cat# 115-035-008) were purchased from Jackson ImmunoResearch Labs.
Validation	Each primary antibody was validated by the commercial supplier, and validation data were provided in the supplier's website.

Eukaryotic cell lines

Policy information about cell lines

Cell line source(s)	MDA-MB-231 (Cat# HTB-26), MDA-MB-468 (Cat# HTB-132), SK-BR-3 (Cat# HTB-30), HCC70 (Cat# CRL-2315), and MCF-7 (Cat# HTB-22) were from American Type Culture Collection. CAL-51 (Cat# ACC-302) was from Deutsche Sammlung von Mikroorganismen und Zellkulturen. MDA-MB-231 (p53 KO) cells were from Dr. Gokul Das' lab at Roswell Park Comprehensive Cancer Center, which were generated from MDA-MB-231 cells by CRISPR-Cas9. MDA-MB-231-DKO cells (p53 KO, and PEPD KO) were generated from MDA-MB-231 (p53 KO) cells in the present study by CRISPR-Cas9. MDA-MB-231 (p53 R175H) cells were generated by transfecting pCMV6-A-human p53 R175H-puro into MDA-MB-231 (p53 KO) cells and selection under puromycin in the present study.
Authentication	The cell lines were authenticated using short tandem repeat analysis in the Genomic Core of Roswell Park Comprehensive Cancer Center. TP53 genotypes in the cell lines were verified by Sanger sequencing.
Mycoplasma contamination	All cell lines test negative for mycoplasma contamination.
Commonly misidentified lines (See ICLAC register)	None.

Animals and other organisms

Policy information about studies involving animals; ARRIVE guidelines recommended for reporting animal research

Laboratory animals	SCID mice (C.B-17 SCID), female, 6-7 weeks of age.
Wild animals	N/A
Field-collected samples	N/A
Ethics oversight	All mouse experiments were approved by the Institutional Animal Care and Use Committee at Roswell Park Comprehensive Cancer Center.

Note that full information on the approval of the study protocol must also be provided in the manuscript.

Flow Cytometry

Plots

Confirm that:

- [ ] The axis labels state the marker and fluorochrome used (e.g. CD4-FITC).
- [ ] The axis scales are clearly visible. Include numbers along axes only for bottom left plot of group (a 'group' is an analysis of identical markers).
- [ ] All plots are contour plots with outliers or pseudocolor plots.
- [x] A numerical value for number of cells or percentage (with statistics) is provided.

Methodology

Sample preparation	Cell lines were cultured in 6-well plates at 200,000 cells per well, including MDA-MB-231 cells, MDA-MB-231 (p53 KO) cells, and CAL-51 cells, or 400,000 SK-BR-3 cells per well in 2 ml medium for 24 h and then treated with scramble siRNA or PEPD siRNA (10 nM) for 48 h. The cells were trypsinized, washed twice with ice-cold PBS, and pelleted by centrifugation at 2,000 rpm for 5 min at 4 oC. The cells were fixed in ice-cold 70% ethanol for at least 30 min at 4 oC. After removing the ethanol by centrifugation and washing the cells twice with ice-cold PBS, the cells from each well were resuspended in 500 µl PI staining buffer containing 20 µg/ml RNase and 50 µg/ml PI, incubated at RT in the dark for 30 min, and then analyzed by flow cytometry.
Instrument	BD LSRFortessa flow cytometer
Software	ModFit LT software.
Cell population abundance	Fifty thousand cells per sample.
Gating strategy	Single cells population was identified by measuring the forward scatter (FSC) and side scatter (SSC). Cell aggregates were excluded from the analysis by pulse processing using the area and width of the DNA content. Analysis of the cell cycle distributions was modeled using the Modfit LT software.

- [ ] Tick this box to confirm that a figure exemplifying the gating strategy is provided in the Supplementary Information.
